# Harnessing a Continuous‐Flow Persulfuric Acid Generator for Direct Oxidative Aldehyde Esterifications

**DOI:** 10.1002/cssc.202201868

**Published:** 2022-12-05

**Authors:** Bence S. Nagy, Gang Fu, Christopher A. Hone, C. Oliver Kappe, Sándor B. Ötvös

**Affiliations:** ^1^ Institute of Chemistry University of Graz NAWI Graz Heinrichstrasse 28 A-8010 Graz Austria; ^2^ Center for Continuous Flow Synthesis and Processing (CC FLOW) Research Center Pharmaceutical Engineering GmbH (RCPE) Inffeldgasse 13 A-8010 Graz Austria

**Keywords:** aldehydes, chemical generators, continuous flow, esters, persulfuric acid

## Abstract

Persulfuric acid is a well‐known oxidant in various industrial‐scale purification procedures. However, due to its tendency toward explosive decomposition, its usefulness in organic synthesis remained largely underexplored. Herein, a continuous in situ persulfuric acid generator was developed and applied for oxidative esterification of aldehydes under flow conditions. Sulfuric acid served as a readily available and benign precursor to form persulfuric acid in situ. By taking advantage of the continuous‐flow generator concept, safety hazards were significantly reduced, whilst a robust and effective approach was ensured for direct transformations of aldehydes to valuable esters. The process proved useful for the transformation of diverse aliphatic as well as aromatic aldehydes, while its preparative capability was verified by the multigram‐scale synthesis of a pharmaceutically relevant key intermediate. The present flow protocol demonstrates the safe, sustainable, and scalable application of persulfuric acid in a manner that would not be amenable to conventional batch processing.

## Graphical abstract

**Acid flow**: A continuous‐flow persulfuric acid generator and its application for direct oxidative esterification of a variety of aldehydes is presented herein. By forming the potentially dangerous oxidant in situ, the safety hazards are significantly reduced, and persulfuric acid is successfully utilized as a simple, robust, and easily scalable oxidant within a closed flow reactor environment.

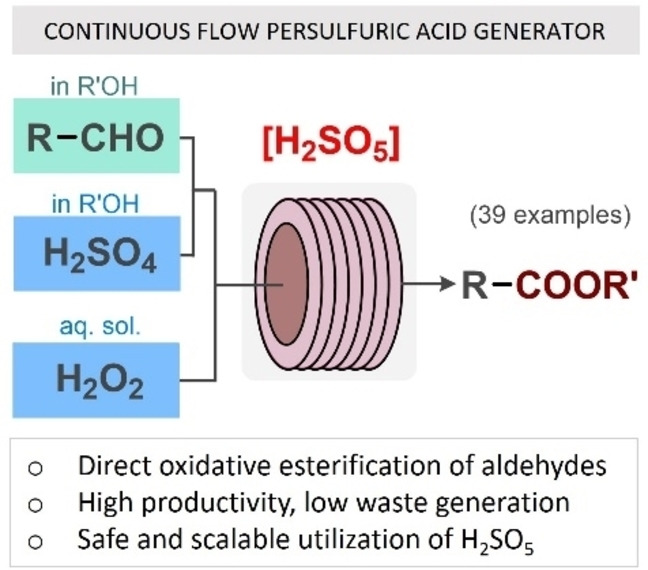

## Introduction

Esters are of fundamental importance in organic chemistry.^[1]^ The direct oxidative esterification of aldehydes has emerged as a sustainable method to access this important class of compounds without isolation of free carboxylic acid intermediates.^[2]^ This is particularly useful in multistep syntheses where incompatible functionalities or protecting groups are present in the substrate. Typical approaches for oxidative aldehyde esterifications rely on various transition metal catalysts in the presence of different oxidants, such as H_2_O_2_, *tert*‐butyl hydroperoxide (TBHP), or O_2_.^[3]^ Such processes generally necessitate costly ligands in combination with special homogeneous or heterogeneous catalysts. In the case of heterogeneous catalysts, uncontrollable leaching issues may significantly reduce practical applications, whilst most homogeneous catalysts employed are very difficult to recover and reuse. Metal‐free protocols employing N‐heterocyclic carbenes as catalysts have also been disclosed.^[4]^ However, similar to metal‐catalyzed strategies, high costs, long reaction times, and limited productivity render them impractical for large‐scale preparations. Noncatalytic processes for oxidative aldehyde esterifications are less explored. Recent approaches utilize, for example, *N*‐bromosuccinimide‐ and TsNBr_2_‐mediated oxidations or exploit trichloroisocyanuric acid to access esters via acyl chloride intermediates.^[5]^ The main drawbacks of these procedures are related to limited substrate scope or the high reagent excess required.

Peroxymonosulfuric acid (also known as persulfuric acid or Caro's acid) is known as an effective oxidant with numerous large‐scale applications in the field of wastewater treatment as well as in mining and hydrometallurgy industries.^[6]^ As its instability and tendency toward explosive decomposition exerts a significant safety hazard,^[7]^ persulfuric acid is generally not stored or transported but manufactured on site.^[8]^ Despite its beneficial features, such as low cost and ease of removal by aqueous extraction, applications of persulfuric acid in synthetic organic chemistry remained largely underexploited due to its hazardous nature.^[9]^ Although the shelf‐stable potassium salt of peroxymonosulfuric acid is also known as an oxidizing agent,^[10]^ its inadequate solubility in most organic solvents is a significant limitation for numerous synthetic transformations.^[11]^

Chemical processes can not only be advanced by new and improved transformations, but also via strategic utilization of enabling technologies.^[12]^ In this manner, the inherent benefits of continuous‐flow reactors enable a unique opportunity to handle highly reactive or short‐lived reagents, which are difficult to handle in conventional batch reactors.^[13]^ In particular, due to the excellent heat and mass transport, the comparatively small reactor volume, and the elimination of headspace, problematic reagents can be generated on‐site and on‐demand from readily available and benign precursors.^[14]^ The so‐called continuous‐flow generator concept has demonstrated exceptional potential to facilitate hazardous chemistries that are not amenable to traditional batch processing in a safe and scalable manner.^[15]^

We recently communicated a three‐step flow process for the enantioselective synthesis of the phosphodiesterase IV inhibitor rolipram, where a chiral γ‐nitroester key intermediate was obtained by means of persulfuric acid‐mediated direct aldehyde esterification.^[16]^ Based on this preliminary finding as well as the well‐established capability of flow reactors for in situ generation and concomitant utilization of highly reactive or other hazardous reagents,^[15]^ we speculated that the synthetic applicability of persulfuric acid can largely be advanced by exploiting the continuous‐flow generator concept. In this manner, by forming the potentially dangerous oxidant in situ, the safety hazards can be minimized and persulfuric acid can be harnessed as a simple and easily scalable oxidant within a closed continuous‐flow reactor environment. Taking the robustness and efficacy of persulfuric acid as oxidizing agent into consideration, we anticipated that such a continuous‐flow protocol would enable a highly productive and general approach for direct oxidative aldehyde esterifications. Our findings are presented herein.

## Results and Discussion

**Figure 1:**
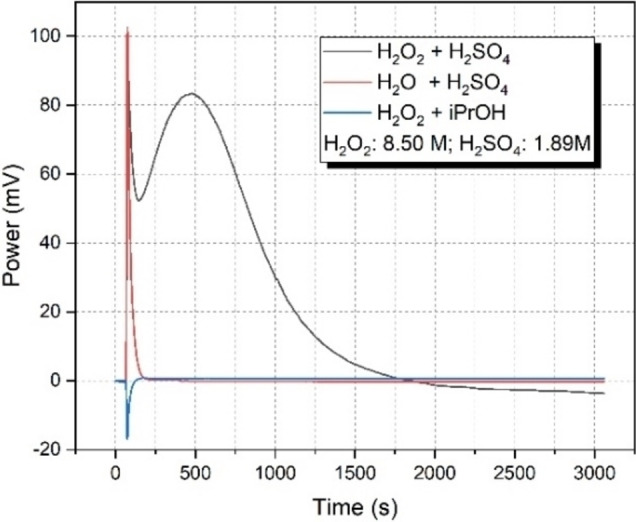
Calorimetric titration test at 80 °C. 30 μL H_2_O_2_ or H_2_O was injected into 270 μL H_2_SO_4_ or *i*PrOH.

Sulfuric acid served as a benign precursor in the presence of H_2_O_2_ to generate persulfuric acid in situ.^[8]^ Before continuous‐flow process development, the thermal behavior of the H_2_SO_4_−H_2_O_2_ reaction system was explored using a micro reaction calorimeter in order to characterize the formation and decomposition of persulfuric acid as well as to evaluate potential safety hazards. Calorimetric titration tests at different temperatures revealed that a temperature above 70 °C was necessary for persulfuric acid formation to occur (Figure S1). A scan test from 25 to 150 °C also demonstrated that the reaction started at around 75 °C (Figure S2). To determine the reaction heat, further titration tests were conducted at 80 °C. Before measuring the actual reaction heat, the mixing heat was assessed by dosing H_2_O to H_2_SO_4_. Subsequently, a H_2_O_2_ solution was injected into *i*PrOH under the same conditions to corroborate that any heat formation was not related to the decomposition of H_2_O_2_. As can be seen in Figure 1, mixing took place at the beginning of the injection and H_2_O_2_ did not decompose as a negative power was observed. The negative power was caused by the temperature difference between H_2_O_2_ in the syringe and *i*PrOH in the sample vial. Hence, the positive power after injection of H_2_O_2_ to H_2_SO_4_ undoubtedly included the formation heat of persulfuric acid. After excluding the mixing heat, the reaction heat was determined to be −271.5±10.1 kJ mol^−1^ (see also Table S1). Considering that only one exothermic peak was observed either in scan or titration mode, the subsequent decomposition of persulfuric acid cannot be ascertained by using such calorimetry experiments. In order to assess this, the total peroxide concentration in the reaction mixture was measured by classical iodometric titration. Controlled persulfuric acid formation was repeated by using the scan mode of the calorimeter from 60 to 80 °C. After the experiment, the remaining peroxide concentration was determined as 7.5×10^−3^ m (see also Table S2), which corresponded to 1.5 % of the total amount of detectable H_2_O_2_. Although the recorded amount is very low and represents all peroxide species, it is still recommended to quench the flow reactions to avoid buildup of any hazardous peroxide and/or peracid. It can be concluded that under the investigated reaction conditions, the degradation of persulfuric acid takes place directly after its formation and that the determined reaction enthalpy (−271.5±10.1 kJ mol^−1^) included both the formation and the decomposition of persulfuric acid. This corresponds to an approximate adiabatic temperature rise of 110 °C, which demonstrates that careful temperature control of the process is necessary.

**Scheme 1:**
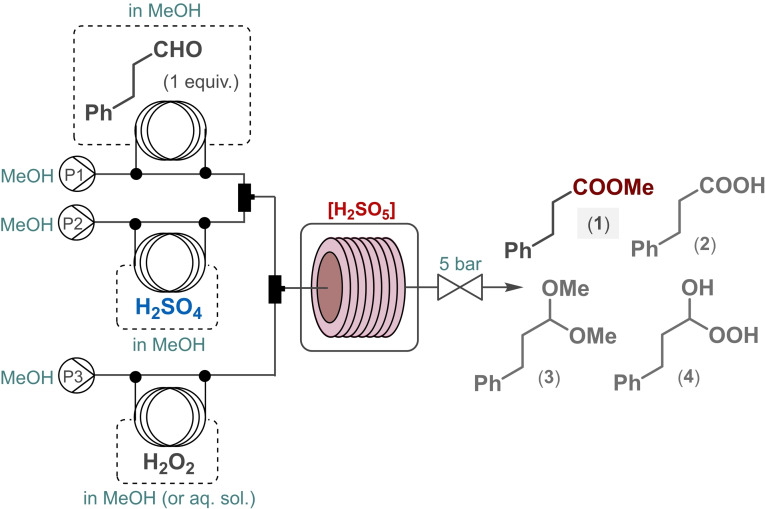
Continuous‐flow setup for the direct oxidative esterification of hydrocinnamaldehyde using in situ‐generated persulfuric acid.

Having acquired sufficient thermal data, a simple flow setup was next constructed for persulfuric acid formation and concomitant oxidative aldehyde esterification in the presence of MeOH as alcohol component. A MeOH solution of H_2_SO_4_ was continuously mixed with a stream of H_2_O_2_ solution in the presence of a constant stream of hydrocinnamaldehyde as model substrate dissolved in MeOH. Each of the component streams were pumped separately, and the combined mixture was passed through a heated reaction coil where controlled generation and utilization of persulfuric acid took place simultaneously (Scheme 1). Oxidative aldehyde esterification in the presence of persulfuric acid is most likely to proceed via successive hemiacetal and acetal intermediates.[^2^, ^17^] Even though persulfuric acid behaves as a strong acid in its first dissociation step,^[18]^ it seemed practical to combine first the substrate and H_2_SO_4_ streams to facilitate hemiacetal/acetal formation. In order to eliminate evolution of gas arising from solvent boil‐over and/or from persulfuric acid decomposition,^[19]^ a 5 bar backpressure regulator (BPR) was also installed. Based on the calorimetric data, incidental peroxide and/or peracid traces had to be quenched continuously. The reactor outlet was therefore directed into a flask containing a stirred mixture of MnO_2_ in saturated aqueous NaHCO_3_ solution.

Investigating the in situ‐generated persulfuric acid‐mediated continuous‐flow oxidative esterification of hydrocinnamaldehyde at 50 °C in the presence of a relatively large excess of H_2_SO_4_ and H_2_O_2_ (4 equiv. of both), only 30 % conversion and 52 % chemical selectivity was detected with the corresponding dimethyl acetal (**3**) as major side product (Table 1, entry 1). This agrees well with the calorimetric data collected earlier and can be explained by incomplete persulfuric acid formation at lower temperatures. Unlike in a traditional batch equipment, the reaction temperature could safely be increased under controlled flow conditions to enhance persulfuric acid formation and thus to improve esterification. Despite reducing the excess of H_2_SO_4_ and H_2_O_2_ to 2 equiv., respectively, the reaction became quantitative at 100 °C with a small amount of hydrocinnamic acid (**2**) detected as the only side product (Table 1, entry 2). Gratifyingly, the oxidative esterification worked well even in case of further reduction of the amount of both reagents to 1.2 equiv., respectively (Table 1, entry 3). According to the thermal data collected at 80 °C, the decomposition of the in situ formed persulfuric acid takes place directly after its formation, and these two concomitant procedures take approximately 20–25 min to complete under the conditions applied for the calorimetric titration test as shown on Figure 1. At 100 °C, the formation and decomposition of persulfuric acid should be even faster (see also Figures S1 and S2) suggesting that the oxidative esterification can possibly be intensified by reduction of the residence time. In fact, ≥95 % conversion and 93 % chemoselectivity were observed utilizing either 20 or 10 min residence time (Table 1, entries 3 and 4), and a further decrease to 2 min still resulted in an acceptable conversion of 72 % and a selectivity towards ester **1** of 71 % (Table 1, entry 5). It was also found that the application of an excess of H_2_SO_4_ with respect to H_2_O_2_ is beneficial for the reaction possibly by shifting the equilibrium of persulfuric acid formation and also because of potential enhancement of dimethyl acetal formation (Table 1, entries 6 and 7).

**Table 1:** Optimization of the in situ‐generated persulfuric acid‐mediated direct oxidative esterification of hydrocinnamaldehyde (see also Scheme 1).

Entry^[a]^	*c* [m]			H_2_SO_4_ [equiv.]	H_2_O_2_ [equiv.]	*t_r_* [min]	*T* [°C]	Conv.^[g]^ [%]	Chemoselectivity^[g]^ [%]				
	Substrate	H_2_SO_4_^[b]^	H_2_O_2_						1	2	3	4	Unid.^[h]^
1	1.0	4.0	2.0^[c]^	4.0	4.0	20	50	30	52	15	26	7	0
2	1.0	4.0	2.0^[c]^	2.0	2.0	20	100	100	95	5	0	0	0
3	1.0	4.0	2.0^[c]^	1.2	1.2	20	100	98	93	4	3	0	0
4	1.0	4.0	2.0^[c]^	1.2	1.2	10	100	95	93	3	5	0	0
5	1.0	4.0	2.0^[c]^	1.2	1.2	2	100	72	71	11	13	0	5
6	1.0	4.0	2.0^[c]^	3.6	1.2	20	100	100	96	4	0	0	0
7	1.0	4.0	2.0^[c]^	1.2	3.6	20	100	96	91	4	1	4	0
8	1.0	8.0	11.6^[d]^	1.2	1.2	2	100	81	76	12	7	1	4
9	1.0	8.0	11.6^[d]^	2.4	1.2	2	100	94	92	7	1	0	0
10	1.0	8.0	11.6^[d]^	2.4	1.2	2	120	100	97	3	0	0	0
11	2.0	8.0	11.6^[d]^	2.4	1.2	2	120	100	87	6	0	5	2
12	3.0	8.0	11.6^[d]^	2.4	1.2	2	120	100	74	7	0	8	11
13	1.0	8.0	11.6^[d]^	2.4	1.2	1	120	96	88	2	10	0	0
14	1.0	8.0	17.6^[e]^	2.4	1.2	2	120	100	97	3	0	0	0
15	1.0	8.0	2.0^[f]^	2.4	1.2	2	120	100	95	3	2	0	0
16	1.0	–	11.6^[d]^	0	1.2	2	100	73	0	2	0	91	7

[a] For the corresponding flow rates, see Table S5 in the Supporting Information. [b] H_2_SO_4_ stock solutions were made using cc. H_2_SO_4_. [c] Stock solutions were made using 35 wt % aq. H_2_O_2_. [d] 35 wt % aq. H_2_O_2_ was streamed. [e] 50 wt % aq. H_2_O_2_ was streamed. [f] UHP was used instead of aq. H_2_O_2_. [g] Determined by high‐performance liquid chromatography (HPLC) area%. [h] Unidentified side products.

With the aim to improve process efficiency and sustainability, the effects of higher reagent concentrations were explored next. The concentration of the H_2_SO_4_ feed was increased from 4.0 to 8.0 m and H_2_O_2_ was streamed directly as a 35 wt % aq. solution without any MeOH, whilst the content of the hydrocinnamaldehyde feed was not changed and was pumped as a 1.0 m solution in MeOH. These modifications were found beneficial and resulted an improvement in conversion and in selectivity towards ester **1** (Table 1, entries 5 vs. 8). Importantly, by increasing the amount of H_2_SO_4_ to 2.4 equiv. and by increasing the reactor temperature to 120 °C, quantitative transformation and an excellent chemical selectivity of 97 % was attained with only a minor amount of hydrocinnamic acid (**2**) formed as side product (Table 1, entries 9 and 10). With these parameters in hand, we next investigated the effects of the concentration of the substrate stream. The best results were obtained utilizing 1.0 m, as any higher concentration led to a significant decrease in conversion and also in selectivity of the transformation (Table 1, entries 10 vs. 11 and 12). Regarding the residence time, 2 min was found as an optimum value, as further decrease led to incomplete esterification as verified by decrease in conversion and also by the appearance of a significant amount of dimethyl acetal **3** (Table 1, entry 13).

**Scheme 2:**
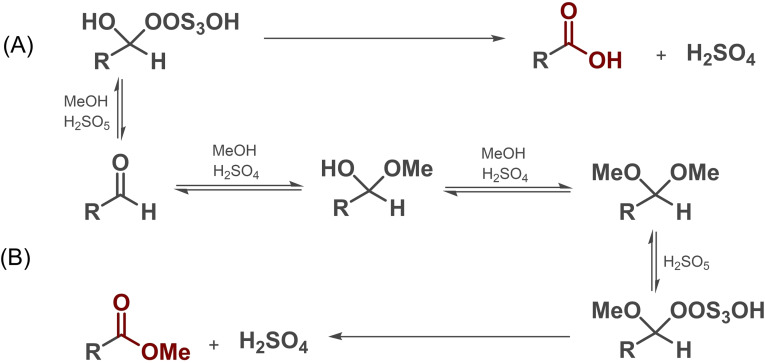
Possible reaction mechanism of persulfuric acid‐mediated oxidative esterification of aldehydes.[^15e^, ^17^]

As an attempt to eliminate the formation of the carboxylic acid side product, the reaction was repeated under the previously optimized conditions but using 50 wt % aq. H_2_O_2_. Compared to the experiment with 35 wt % aq. H_2_O_2_, no change in product distribution was observed (Table 1, entries 10 vs. 14), which suggested that **2** is formed in a competitive Baeyer–Villiger‐like aldehyde to carboxylic acid oxidation rather than in acid‐catalyzed hydrolysis of methyl ester **1** (Scheme 2A).[^15e^, ^17^] In addition to the use of aq. H_2_O_2_ solutions, the application of solid urea hydrogen peroxide (UHP) in MeOH solution also provided very good results for persulfuric acid generation and concomitant aldehyde esterification (Table 1, entry 15). However, it should be noted that the utilization of UHP is less atom effective than that of H_2_O_2_.

Although during parameter optimization the formation of dimethyl acetal **3** was frequently detected as an intermediate indicating incomplete transformation (Scheme 2B), the appropriate hemiacetal was not observed, which is possibly due to its rapid transformation to acetal under strongly acidic conditions. In some of the reactions (Table 1, entries 1, 7, 8, 11, and 12), perhydrate **4** was also detected as a side product. As corroborated by a control experiment conducted without any H_2_SO_4_ present (Table 1, entry 16), compound **4** was formed directly in reaction with small amounts of unconverted H_2_O_2_.

In addition to the in situ‐generated persulfuric acid‐mediated flow process, further approaches for direct oxidative aldehyde esterifications were attempted and evaluated. Our earlier findings on continuous‐flow oxidation of aldehydes to carboxylic acids by using in situ‐formed performic acid as the oxidant were found ineffective for oxidative esterifications due to very low ester selectivity in the presence of MeOH as alcohol component.[^15e^, ^15g^, ^16^] During our preliminary investigations, *p*‐toluenesulfonic peracid generated in situ from TsOH also showed potential as an oxidizing agent in oxidative ester formations.^[16]^ However, for quantitative removal of residual TsOH, chromatographic purification was required. In contrast, in case of the persulfuric acid mediated process, the pure esters could be isolated by simple extractive workup without chromatography. On the basis of earlier reports on hypochlorite mediated aldehyde oxidations,^[20]^ the H_2_O_2_/HCl system was also explored. In this manner, hypochlorous acid, a potential oxidant for direct aldehyde esterifications,^[21]^ was generated and utilized in situ under continuous‐flow conditions. However, due to competing α‐chlorination and subsequent hydroxylation,^[22]^ the corresponding α‐hydroxylated acetal was formed in considerable amounts in addition to the desired ester. Despite attempts to optimize the reaction conditions (Table S3), the best ester selectivity achieved was <60 %, rendering the process impractical for further consideration. We speculated that immobilized sulfonic peracids may have some potential for oxidative aldehyde esterifications. Sulfonic acid‐based solid acids Amberlyst 15 and Dowex 50WX8 were therefore also investigated in the presence of various oxidants using hydrocinnamaldehyde as a model substrate. After parameter screening in a simple packed‐bed flow system, high yielding esterification (up to 96 % conversion and 96 % selectivity) was observed with Dowex 50WX8 in the presence of H_2_O_2_ as oxidant (Table S4). However, the relatively long residence time of 20 min and the relatively large H_2_O_2_ excess of 3 equiv., along with the rapid deactivation of such solid acids should be considered as significant disadvantages limiting continuous and effectively scalable synthetic applications.

With an optimal set of reaction conditions in hand, the scope and generality of the persulfuric acid mediated oxidative esterification was investigated next. The esterification of hydrocinnamaldehyde worked well not only with MeOH, but also with EtOH and *i*PrOH, even though some decrease of conversion and/or ester selectivity was observed with these bulkier alcohols (Table 2, entries 1–3). Besides hydrocinnamaldehyde, its β‐methyl‐ and *p*‐methoxy‐substituted derivatives, as well as 4‐phenylbutyraldehyde were also tolerated by the optimized reaction conditions furnishing quantitative conversion and a chemoselectivity of ≥90 % in each reaction (Table 2, entries 4–6). Aldehydes bearing the benzyl moiety were transformed smoothly to the corresponding methyl esters; however, the formation of some unidentified side products was detected in these cases (Table 2, entries 7 and 8). The oxidative esterification of cinnamaldehyde proved to be a limitation, which was mainly due to side reactions of the conjugated double bond (Table 2, entry 9). In contrast, phenylpropargylaldehyde, a substrate containing a conjugated C≡C triple bond, furnished a reasonable chemoselectivity of 79 % towards the desired methyl ester (Table 2, entry 10). The flow process proved very effective for transforming various aliphatic aldehydes to the corresponding methyl esters. In all reactions, excellent conversion (≥99 %) and high selectivity (up to >99 %) were detected (Table 2, entries 11–18). In cases of α‐branched aliphatic aldehydes, oxidative aldehyde deformylation resulted small amounts of the corresponding alcohol as side product (Table 2, entries 11–14).^[23]^ In reactions of open chain and β‐branched aldehydes (Table 2, entries 15–18), no alcohol formation was detected. Gratifyingly, in oxidative esterification of undecylenic aldehyde, the C=C double bond remained intact, and the desired methyl ester was achieved with a selectivity of 95 % (Table 2, entry 18).

**Table 2:** Investigation of the in situ‐generated persulfuric acid‐mediated direct oxidative esterification of various of aliphatic aldehydes.

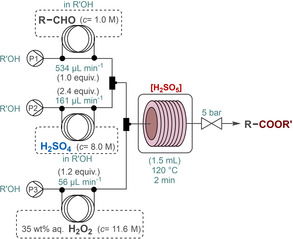				
Entry^[a]^	Substrate	Alcohol	Conv.^[b]^ [%]	Chemosel.^[b,d]^ [%]
1	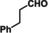	MeOH	100 (95)^[c]^	98
2		EtOH	100	86
3		*i*PrOH	88	73^[e]^
4	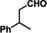	MeOH	100 (89)^[c]^	92
5	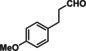	MeOH	100	91
6	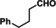	MeOH	100 (85)^[c]^	90^[f]^
7	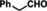	MeOH	99	84^[g]^
8	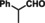	MeOH	98	73^[h]^
9	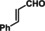	MeOH	85	29^[f]^
10	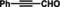	MeOH	94	79^[i]^
11	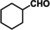	MeOH	100	91^[j]^
12	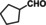	MeOH	100	95^[j]^
13	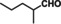	MeOH	99	92^[j]^
14	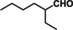	MeOH	100	84^[j]^
15	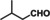	MeOH	100 (97)^[c]^	>99
16	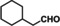	MeOH	100	98
17	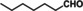	MeOH	100	98
18	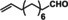	MeOH	99	95

[a] H_2_SO_4_ stock solutions were made using cc. H_2_SO_4_. [b] Determined by HPLC or GC‐FID area%. [c] Yield of isolated product. [d] Unless otherwise stated, the corresponding carboxylic acid was formed as typical side product. [e] 19 % carboxylic acid, 8 % unidentified side product. [f] Unidentified side products. [g] 5 % carboxylic acid, 11 % unidentified side product. [h] 3 % carboxylic acid, 24 % unidentified side product. [i] 8 % carboxylic acid, 13 % unidentified side product. [j] The corresponding alcohol from oxidative aldehyde deformylation was formed as side product.

Diversely substituted aromatic aldehydes were explored next as substrates for the persulfuric acid‐mediated oxidative esterification. In reaction of benzaldehyde, it was observed that under the conditions employed earlier for aliphatic aldehydes relatively high amount of carboxylic acid side product was formed (Table 3, entry 1). In contrast, in cases of benzaldehyde derivatives decorated with strongly electron‐withdrawing substituents, such as trifluoromethyl, nitrile and fluorine, the conditions introduced earlier furnished the corresponding esters with only minor amounts of carboxylic acid side product formed (Table 3, entries 3–8). For example, *p*‐(trifluoromethyl)benzaldehyde was effectively transformed to the corresponding methyl ester without any side product detected (Table 3, entry 3). Also, fluorine‐substituted benzaldehydes furnished the desired esters with chemical selectivities up to 96 % regardless of the position of the substituent on the aromatic ring (Table 3, entries 5–7). Even in the case of the sterically hindered 2,6‐difluorobenzaldehyde, 92 % conversion and 81 % ester selectivity was obtained (Table 3, entry 8). In order to minimize carboxylic acid formation in reactions of electron rich aromatic aldehydes, additional parameter optimization was performed using benzaldehyde as substrate (Table S6). Selectivity of ester formation was found to be independent of typical reaction parameters, such as residence time and temperature. However, by exchanging the aq. H_2_O_2_ solution, required for in situ persulfuric acid generation, to 2.0 m UHP in MeOH without modifying any other reaction parameters, a considerable increase was attained in selectivity of ester formation (83 vs. 91 %; Table 3, entries 1 vs. 2). With this modification in hand, several reactions involving more electron rich aromatic aldehydes were attempted. Good results (83–100 % conversion and 44–98 % selectivity) were achieved with a diverse set of methoxy‐ or methyl‐substituted benzaldehydes as well as with 2‐naphthaldehyde (Table 3, entries 9–13), even though in some instances the residence time had to be increased from 2 to 4 min and substrate concentration had to be decreased from 1.0 to 0.5 m due to clogging issues. Various chloro‐ and bromo‐substituted benzaldehydes were also benefiting from the modified conditions utilizing UHP and furnished conversions up to 99 % and chemical selectivities between 73 and 97 % (Table 3, entries 14–16). Due to precipitation issues, nitro‐substituted benzaldehydes were also reacted in the presence of UHP in MeOH rather than using aq. H_2_O_2_ solution (Table 3, entries 17 and 18). Of heteroaromatic aldehydes, nicotinaldehyde and 6‐bromopicolinaldehyde furnished high conversions and good selectivities (Table 3, entries 19 and 20); whereas, in case of thiophene‐2‐carbaldehyde, selectivity was lower due to carboxylic acid formation and also due to further side reactions related to possible thiophene ring opening (Table 3, entry 21). For selected examples of the substrate scope, ester products were isolated in yields of 85–97 % (Table 2, entries 1, 4, 6, and 15; Table 3 entries 2, 3, 6, and 16).

**Table 3:** Investigation of in the in situ‐generated persulfuric acid‐mediated direct oxidative esterification of various of aromatic aldehydes.

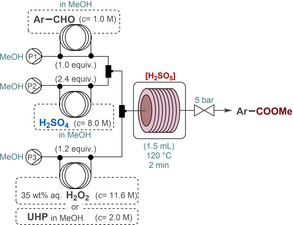				
Entry^[a]^	Substrate	Oxidant	Conv.^[d]^ [%]	Chemosel.^[d,f]^ [%]
1	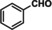	H_2_O_2_	100	83
2		UHP	99 (87)^[e]^	91
3	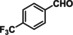	H_2_O_2_	100 (97)^[e]^	>99
4	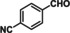	H_2_O_2_	100	94
5	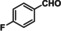	H_2_O_2_	100	90
6	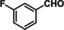	H_2_O_2_	100 (92)^[e]^	96
7	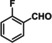	H_2_O_2_	98	91^[g]^
8	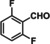	H_2_O_2_	92	81^[g]^
9^[b]^	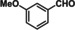	UHP	83	98
10	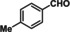	UHP	100	44
11	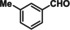	UHP	98	73
12	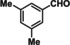	UHP	94	66^[h]^
13^[b,c]^	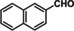	UHP	99	51
14	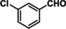	UHP	93	97
15^[b]^	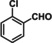	UHP	84	73^[g]^
16	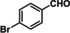	UHP	99 (89)^[e]^	93
17^[b,c]^	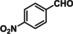	UHP	100	88^[i]^
18^[c]^	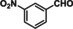	UHP	97	91^[j]^
19	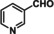	H_2_O_2_	94	75^[k]^
20	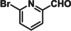	H_2_O_2_	100	86^[l]^
21	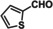	UHP	100	39^[m]^

[a] H_2_SO_4_ stock solutions were made using cc. H_2_SO_4_. For the corresponding flow rates, see Table S7 in the Supporting Information. [b] 4 min residence time. [c] 0.5 m substrate concentration, 4.0 m H_2_SO_4_ concentration. [d] Determined by HPLC area%. [e] Yield of isolated product. [f] Unless otherwise stated, the corresponding carboxylic acid was formed as typical side product. [g] Unidentified side products. [h] 27 % carboxylic acid, 7 % unidentified side product. [i] 2 % carboxylic acid, 10 % unidentified side product. [j] 3 % carboxylic acid,6 % unidentified side product. [k] 18 % carboxylic acid, 7 % unidentified side product. [l] 9 % carboxylic acid, 5 % unidentified side product. [m] 46 % carboxylic acid, 15 % unidentified side product.

**Scheme 3:**
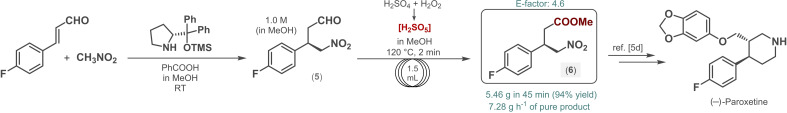
Continuous‐flow gram‐scale synthesis of the key γ‐nitroester intermediate of (−)‐paroxetine by means of in situ‐generated persulfuric acid‐mediated direct oxidative aldehyde esterification. Substrate feed: 534 μL min^−1^, H_2_SO_4_ feed (8.0 m, in MeOH): 161 μL min^−1^ (corresponds to 2.4 equiv.), H_2_O_2_ feed (35 wt % aq. solution): 56 μL min^−1^ (corresponds to 1.2 equiv.). See also section 3.3.4 in the Supporting Information.

To demonstrate the practical utility of the persulfuric acid‐mediated flow process, the multigram‐scale synthesis of a key intermediate of a well‐known active pharmaceutical ingredient (API), (−)‐paroxetine was attempted. (−)‐Paroxetine is a selective serotonin reuptake inhibitor, which is prescribed widely for the treatment of depression and panic disorder.^[24]^ There are different approaches reported for the enantioselective synthesis of (−)‐paroxetine.^[25]^ We targeted the preparation of chiral γ‐nitroester **6**, which can readily be transformed to the actual API according to literature procedures.^[5d]^ First, γ‐nitroaldehyde **5** was prepared by means of Michael‐type addition of nitromethane to 4‐fluorocinnamaldehyde in the presence of a diphenylprolinol trimethylsilyl (TMS) ether as a chiral organocatalyst. Aldehyde **5** was next transformed directly to methyl ester **6** by means of persulfuric acid mediated oxidative esterification utilizing the flow conditions developed earlier for the transformation of hydrocinnamaldehyde (Scheme 3). A 45 min run was performed under steady‐state conditions. Gratifyingly, the flow system proved robust during the experiment, no clogging or any other issues were observed. The flow process furnished quantitative and selective esterification, thus analytically pure product was obtained after simple extractive work‐up. 5.46 g of pure γ‐nitroester **6** was prepared which correlates to a space‐time‐yield (STY) of 4.85 kg L^−1^ h^−1^ and a productivity of 7.28 g h^−1^.

In order to assess process sustainability and environmental impacts, quantitative green metrics for the persulfuric acid‐mediated continuous flow oxidative esterification of **5** were calculated and compared directly to existing batch data (Table 4). In fact, we found only one literature precedent for the oxidative esterification of γ‐nitroaldehyde **5** to γ‐nitroester **6** utilizing *N*‐bromosuccinimide (NBS)‐based oxidation (Scheme S2);^[5d]^ the data reported there was used for the analysis. E‐factor, process mass intensity (PMI), reaction mass efficiency (RME), atom economy (AE), and optimum efficiency (OE) were calculated using equations reported in the literature (see also section 3.3.5 in the Supporting Information).^[26]^ The E‐factor as well as the PMI is over 2‐fold lower for the H_2_SO_5_‐mediated flow process when compared to the NBS‐mediated batch procedure. In addition, the flow process performs better in terms of RME and AE, therefore it also has a higher OE. These results demonstrate that the flow process (i) is environmentally more effective, (ii) generates less waste, and thus (iii) it is more sustainable.

**Table 4:** Comparison of green metrics for the persulfuric acid‐mediated continuous‐flow oxidative esterification of **5** to literature batch data.

Metric^[a]^	H_2_SO_5_‐mediated flow process^[b]^	NBS‐mediated batch procedure^[c]^
conversion [%]	>99	>99
yield [%]	94	63
E‐factor	4.6	12.6
PMI	6.0	13.6
RME	46.6	31.8
AE	70.3	62.0
OE	66.3	51.3

[a] The values used for the calculations are shown in Tables S8 and S9 in the Supporting Information. [b] Calculations are based on data reported herein. [c] Calculations are based on data taken from Ref. [5d].

## Conclusion

A flow process was developed and generalized for direct oxidative esterification of aldehydes utilizing persulfuric acid as an effective oxidant. The safety hazards associated with the handling of persulfuric acid were minimized by exploiting a continuous‐flow generator. The improvement in terms of safety management compared to previously reported approaches for production of persulfuric acid lies in the fully continuous nature of the present process where formation and chemical consumption of the potentially dangerous oxidant takes place in the same space simultaneously within a well‐defined and short residence time (typically 2 min).^[8]^

In order to assess potential safety hazards during formation and decomposition of persulfuric acid, calorimetric measurements as well as classical analytical titrations were performed. It was verified that despite the higher temperatures utilized, small peracid traces may still exit the reactor thereby necessitating a proper quenching procedure. The effects of the most important reaction conditions of the oxidative esterification were explored carefully in order to minimize waste formation, whilst maximizing productivity and sustainability. Notably, the continuous‐flow protocol did not require any special flow equipment but only a simple coil reactor from commercially available components and no additional solvent was required apart from the alcohol constituent of the esterification. The generality of the process was verified through oxidative esterifications of a series of aliphatic and aromatic model substrates. In most of the examples, excellent conversions and highly selective transformations were achieved where the targeted esters could typically be accessed without chromatographic purification. The preparative usefulness of the process was demonstrated by the highly productive synthesis of an advanced chiral intermediate of the antidepressant (−)‐paroxetine.

In comparison with earlier methodologies, this process represents a cost‐effective and easily scalable entry into direct aldehyde esterifications, whilst at the same time, being efficient from environmental aspects. We believe that the continuous‐flow generator concept renders persulfuric acid as a simple yet effective oxidant that will enable future synthetic applications at various scales.

## Experimental Section

Equipment for the continuous‐flow reactions was assembled using commercially available components. Liquid streams were pumped by using Syrris® Asia syringe pumps. Flow systems were pressurized by using an adjustable BPR from Zaiput. Reaction coils were heated by means of a conventional oil bath. Reagent feeds were either streamed directly or by using injection valves and sample loops. Sample loops and reactor coils were made by using perfluoroalkoxy alkane (PFA) tubing (1/16” OD, 0.80 mm ID). A micro reaction calorimeter (μRC) from Thermal Hazard Technology was used to study the thermal behavior of the H_2_SO_4_−H_2_O_2_ reaction system. Details on instrumentation, reaction setups as well as general experimental procedures can be found in the Supporting Information.

CAUTION: Sulfuric acid is highly corrosive causing rapid tissue destruction and serious chemical burns. Persulfuric acid is one of the strongest oxidants known. It is unstable and potentially explosive, especially in mixtures with organic substances. Extreme care must therefore be taken when handling these substances! All equipment must be set up in a well‐ventilated fume hood and personal protective equipment must be worn during experimentation. A thorough safety assessment should be made before conducting any experiments.

## Conflict of interest

The authors declare no conflict of interest.

1

## Supporting information

As a service to our authors and readers, this journal provides supporting information supplied by the authors. Such materials are peer reviewed and may be re‐organized for online delivery, but are not copy‐edited or typeset. Technical support issues arising from supporting information (other than missing files) should be addressed to the authors.

Supporting Information

## Data Availability

The data that support the findings of this study are available in the supplementary material of this article.

## References

[1] J. Otera , J. Nishikido , in Esterification: methods, reactions, and applications, John Wiley & Sons, 2009.

[2a] S. Gaspa , A. Porcheddu , L. De Luca , Tetrahedron Lett. 2016, 57, 3433–3440;

[2b] S. Tang , J. Yuan , C. Liu , A. Lei , Dalton Trans. 2014, 43, 13460–13470; 25058479 10.1039/c4dt01133c

[2c] K. Ekoue-Kovi , C. Wolf , Chem. Eur. J. 2008, 14, 6302–6315. 18523938 10.1002/chem.200800353

[3] For selected examples, see:

[3a] J. Li , S. Wang , H. Li , Y. Tan , Y. Ding , Int. J. Mol. Sci. 2021, 22, 8668;34445372

[3b] R. Wang , H. Liu , C. Fan , J. Gao , C. Chen , Z. Zheng , J. Mol. Catal. 2020, 484, 110687;

[3c] Y.-F. Guo , B.-H. Xu , T. Li , L. Wang , S.-J. Zhang , Org. Chem. Front. 2016, 3, 47–52;

[3d] S. Dey , S. K. Gadakh , A. Sudalai , Org. Biomol. Chem. 2015, 13, 10631–10640; 26340404 10.1039/c5ob01586c

[3e] M. Caporaso , G. Cravotto , S. Georgakopoulos , G. Heropoulos , K. Martina , S. Tagliapietra , Beilstein J. Org. Chem. 2014, 10, 1454–1461; 24991300 10.3762/bjoc.10.149 PMC4077537

[3f] K. Suzuki , T. Yamaguchi , K. Matsushita , C. Iitsuka , J. Miura , T. Akaogi , H. Ishida , ACS Catal. 2013, 3, 1845–1849;

[3g] Y. Zhu , H. Yan , L. Lu , D. Liu , G. Rong , J. Mao , J. Org. Chem. 2013, 78, 9898–9905; 23998269 10.1021/jo4016387

[3h] B. A. Tschaen , J. R. Schmink , G. A. Molander , Org. Lett. 2013, 15, 500–503; 23320580 10.1021/ol303298g PMC3568929

[3i] D. Talukdar , K. Sharma , S. K. Bharadwaj , A. J. Thakur , Synlett 2013, 24, 963–966;

[3j] C. Liu , S. Tang , L. Zheng , D. Liu , H. Zhang , A. Lei , Angew. Chem. Int. Ed. 2012, 51, 5662–5666; 10.1002/anie.201201960 22539112

[3k] X.-F. Wu , C. Darcel , Eur. J. Org. Chem. 2009, 2009, 1144–1147.

[4a] W. Harnying , P. Sudkaow , A. Biswas , A. Berkessel , Angew. Chem. Int. Ed. 2021, 60, 19631–19636; 10.1002/anie.202104712 PMC8457137 34010504

[4b] M. T. Berry , D. Castrejon , J. E. Hein , Org. Lett. 2014, 16, 3676–3679; 24988367 10.1021/ol501458p

[4c] E. E. Finney , K. A. Ogawa , A. J. Boydston , J. Am. Chem. Soc. 2012, 134, 12374–12377; 22768916 10.1021/ja304716r

[4d] S. De Sarkar , S. Grimme , A. Studer , J. Am. Chem. Soc. 2010, 132, 1190–1191; 20055393 10.1021/ja910540j

[4e] B. E. Maki , K. A. Scheidt , Org. Lett. 2008, 10, 4331–4334. 18759433 10.1021/ol8018488 PMC2657179

[5a] S. Gaspa , A. Porcheddu , L. De Luca , Org. Lett. 2015, 17, 3666–3669; 26161512 10.1021/acs.orglett.5b01579

[5b] K. K. Rajbongshi , M. J. Sarma , P. Phukan , Tetrahedron Lett. 2014, 55, 5358–5360;

[5c] C. B. Kelly , M. A. Mercadante , R. J. Wiles , N. E. Leadbeater , Org. Lett. 2013, 15, 2222–2225; 23614873 10.1021/ol400785d

[5d] K. L. Jensen , P. H. Poulsen , B. S. Donslund , F. Morana , K. A. Jørgensen , Org. Lett. 2012, 14, 1516–1519. 22376002 10.1021/ol3002514

[6a] D. M. Hewitt , A. M. Simons , P. L. Breuer , Can. Metall. Q. 2015, 54, 261–268;

[6b] L. A. C. Teixeira , J. P. M. Andia , L. Yokoyama , F. V. da Fonseca Araújo , C. M. Sarmiento , Miner. Eng. 2013, 45, 81–87.

[7] H. G. Schmidt , ACS Chem. Health Saf. 2022, 29, 54–61.

[8a] H. M. Castrantas, J. L. Manganaro, R. J. Mikida, W. Johnson (FMC Corporation, Philadelphia, PA), US5879653, **1999**;

[8b] I. R. Dilber (FMC Corporation, Philadelphia, PA), WO1997026215 A1, **1997**;

[8c] J. R. G. Lane, C. F. Mcdonogh, S. E. Woods (Solvay Interox Ltd, Warrington, UK), WO1992007791 A1, **1992**;

[8d] E. Jourdan-Laforte (Air Liquide S.A., Paris, France), US3900555, **1975**, https://patents.google.com/patent/US3900555A/en?oq=US3900555.

[9a] G. Y. Ishmuratov , V. A. Vydrina , M. P. Yakovleva , Y. A. Galkina , I. F. Lobko , R. R. Muslukhov , E. M. Vyrypaev , A. G. Tolstikov , Russ. J. Org. Chem. 2012, 48, 1210–1215;

[9b] H. A. Oskooie , M. M. Heravi , N. Karimi , M. E. Zadeh , Synth. Commun. 2011, 41, 436–440;

[9c] B. Movassagh , M. M. Lakouraj , K. Ghodrati , Synth. Commun. 1999, 29, 3597–3603;

[9d] A. E. Hauck , C. S. Giam , Synth. Commun. 1978, 8, 109–115;

[9e] A. Nishihara , I. Kubota , J. Org. Chem. 1968, 33, 2525–2526;

[9f] R. Robinson , L. H. Smith , J. Chem. Soc. 1937, 371–374.

[10] Usually, potassium peroxymonosulfate refers to the triple salt known as Oxone.

[11] H. Hussain , I. R. Green , I. Ahmed , Chem. Rev. 2013, 113, 3329–3371. 23451713 10.1021/cr3004373

[12a] M. Baumann , T. S. Moody , M. Smyth , S. Wharry , Org. Process Res. Dev. 2020, 24, 1802–1813; 10.1021/acs.oprd.4c00213 PMC11334174 39171129

[12b] S. Sharma , J. Das , W. M. Braje , A. K. Dash , S. Handa , ChemSusChem 2020, 13, 2859–2875. 32212245 10.1002/cssc.202000317

[13a] A. Bonner , A. Loftus , A. C. Padgham , M. Baumann , Org. Biomol. Chem. 2021, 19, 7737–7753; 34549240 10.1039/d1ob01452h

[13b] N. Kockmann , P. Thenée , C. Fleischer-Trebes , G. Laudadio , T. Noël , React. Chem. Eng. 2017, 2, 258–280;

[13c] M. Movsisyan , E. I. P. Delbeke , J. K. E. T. Berton , C. Battilocchio , S. V. Ley , C. V. Stevens , Chem. Soc. Rev. 2016, 45, 4892–4928; 27453961 10.1039/c5cs00902b

[13d] J.-i. Yoshida , Y. Takahashi , A. Nagaki , Chem. Commun. 2013, 49, 9896–9904. 10.1039/c3cc44709j 24042967

[14a] S. B. Ötvös , C. O. Kappe , Green Chem. 2021, 23, 6117–6138; 34671222 10.1039/d1gc01615f PMC8447942

[14b] S. Govaerts , A. Nyuchev , T. Noel , J. Flow Chem. 2020, 10, 13–71;

[14c] F. Ferlin , D. Lanari , L. Vaccaro , Green Chem. 2020, 22, 5937–5955;

[14d] R. Gérardy , N. Emmanuel , T. Toupy , V.-E. Kassin , N. N. Tshibalonza , M. Schmitz , J.-C. M. Monbaliu , Eur. J. Org. Chem. 2018, 2018, 2301–2351;

[14e] R. Porta , M. Benaglia , A. Puglisi , Org. Process Res. Dev. 2016, 20, 2–25;

[14f] B. Gutmann , D. Cantillo , C. O. Kappe , Angew. Chem. Int. Ed. 2015, 54, 6688–6728; 10.1002/anie.201409318 25989203

[14g] M. Baumann , I. R. Baxendale , Beilstein J. Org. Chem. 2015, 11, 1194–1219. 26425178 10.3762/bjoc.11.134 PMC4578405

[15a] For a recent review, see: D. Dallinger , B. Gutmann , C. O. Kappe , Acc. Chem. Res. 2020, 53, 1330–1341. For selected examples, see:32543830 10.1021/acs.accounts.0c00199 PMC7467564

[15b] Y. Chen , S. Renson , J.-C. M. Monbaliu , Angew. Chem. Int. Ed. 2022, 61, e2022101; 10.1002/anie.202210146 PMC9825874 35971898

[15c] V.-E. H. Kassin , D. V. Silva-Brenes , T. Bernard , J. Legros , J.-C. M. Monbaliu , Green Chem. 2022, 24, 3167–3179;

[15d] D. V. Silva-Brenes , N. Emmanuel , V. López Mejías , J. Duconge , C. Vlaar , T. Stelzer , J.-C. M. Monbaliu , Green Chem. 2022, 24, 2094–2103;

[15e] M. Prieschl , S. B. Ötvös , C. O. Kappe , ACS Sustainable Chem. Eng. 2021, 9, 5519–5525;

[15f] A. Steiner , J. D. Williams , O. de Frutos , J. A. Rincón , C. Mateos , C. O. Kappe , Green Chem. 2020, 22, 448–454;

[15g] S. B. Ötvös , P. Llanes , M. A. Pericàs , C. O. Kappe , Org. Lett. 2020, 22, 8122–8126; 33026815 10.1021/acs.orglett.0c03100 PMC7573919

[15h] T. von Keutz , D. Cantillo , C. O. Kappe , Org. Lett. 2019, 21, 10094–10098; 31794232 10.1021/acs.orglett.9b04072

[15i] M. Köckinger , C. A. Hone , C. O. Kappe , Org. Lett. 2019, 21, 5326–5330; 31247792 10.1021/acs.orglett.9b01941

[15j] H. Yang , B. Martin , B. Schenkel , Org. Process Res. Dev. 2018, 22, 446–456;

[15k] G. Glotz , R. Lebl , D. Dallinger , C. O. Kappe , Angew. Chem. Int. Ed. 2017, 56, 13786–13789; 10.1002/anie.201708533 28877406

[15l] D. Dallinger , C. O. Kappe , Nat. Protoc. 2017, 12, 2138–2147; 28906494 10.1038/nprot.2017.046

[15m] M. A. Ganiek , M. R. Becker , G. Berionni , H. Zipse , P. Knochel , Chem. Eur. J. 2017, 23, 10280–10284; 28590518 10.1002/chem.201702593

[15n] H. Lehmann , Green Chem. 2017, 19, 1449–1453;

[15o] D. Dallinger , V. D. Pinho , B. Gutmann , C. O. Kappe , J. Org. Chem. 2016, 81, 5814–5823; 27359257 10.1021/acs.joc.6b01190

[15p] S. T. R. Müller , T. Hokamp , S. Ehrmann , P. Hellier , T. Wirth , Chem. Eur. J. 2016, 22, 11940–11942. 27339757 10.1002/chem.201602133

[16] B. S. Nagy , P. Llanes , M. A. Pericas , C. O. Kappe , S. B. Ötvös , Org. Lett. 2022, 24, 1066–1071. 35050638 10.1021/acs.orglett.1c04300 PMC8822492

[17a] M. Curini , F. Epifano , M. C. Marcotullio , O. Rosati , Synlett 1999, 6, 777–779;

[17b] J. C. Craig , E. Horning , J. Org. Chem. 1960, 25, 2098–2102;

[17c] B. R. Travis , M. Sivakumar , G. O. Hollist , B. Borhan , Org. Lett. 2003, 5, 1031–1034. 12659566 10.1021/ol0340078

[18] G. Lente , J. Kalmár , Z. Baranyai , A. Kun , I. Kék , D. Bajusz , M. Takács , L. Veres , I. Fábián , Inorg. Chem. 2009, 48, 1763–1773. 19159267 10.1021/ic801569k

[19] A. Lange , H.-D. Brauer , J. Chem. Soc. Perkin Trans. 2 1996, 805–811.

[20a] Y. Zhang , S. C. Born , K. F. Jensen , Org. Process Res. Dev. 2014, 18, 1476–1481;

[20b] A. B. Leduc , T. F. Jamison , Org. Process Res. Dev. 2012, 16, 1082–1089;

[20c] K. Bahrami , M. M. Khodaei , S. Kamali , Chin. J. Chem. 2008, 26, 1119–1121;

[20d] E. Dalcanale , F. Montanari , J. Org. Chem. 1986, 51, 567–569;

[20e] S. Sugai , T. Kodama , S. Akaboshi , S. Ikegami , Chem. Pharm. Bull. 1984, 32, 99–105;

[20f] R. V. Stevens , K. T. Chapman , C. A. Stubbs , W. W. Tam , K. F. Albizati , Tetrahedron Lett. 1982, 23, 4647–4650.

[21] T. Takeda , H. Watanabe , T. Kitahara , Synlett 1997, 1997, 1149–1150.

[22a] M. J. Zacuto , D. Cai , Tetrahedron Lett. 2005, 46, 447–450;

[22b] M. Boni , L. Forti , F. Ghelfi , U. M. Pagnoni , Tetrahedron 1994, 50, 7897–7902.

[23a] Q. Zhang , A. Bell-Taylor , F. M. Bronston , J. D. Gorden , C. R. Goldsmith , Inorg. Chem. 2017, 56, 773–782; 28004924 10.1021/acs.inorgchem.6b02127

[23b] A. Kipke , K.-U. Schöning , M. Yusubov , A. Kirschning , Eur. J. Org. Chem. 2017, 2017, 6906–6913.

[24] B. Green , Curr. Med. Res. Opin. 2003, 19, 13–21. 12661775 10.1185/030079902125001353

[25] C. De Risi , G. Fanton , G. P. Pollini , C. Trapella , F. Valente , V. Zanirato , Tetrahedron: Asymmetry 2008, 19, 131–155.

[26] C. R. McElroy , A. Constantinou , L. C. Jones , L. Summerton , J. H. Clark , Green Chem. 2015, 17, 3111–3121.

