# Supplementary material for: Harnessing a Continuous‐Flow Persulfuric Acid Generator for Direct Oxidative Aldehyde Esterifications
Source: ChemSusChem. 2022 Dec 5;16(2):e202201868. doi: 10.1002/cssc.202201868 (PMC10107610; doi:10.1002/cssc.202201868)
Supplement: Supplementary file 1 — Supporting Information [file CSSC-16-0-s001.pdf]

ChemSusChem

Supporting Information

# Harnessing a Continuous-Flow Persulfuric Acid Generator for Direct Oxidative Aldehyde Esterifications

Bence S. Nagy, Gang Fu, Christopher A. Hone, C. Oliver Kappe,* and Sándor B. Ötvös*© 2022 The Authors. ChemSusChem published by Wiley-VCH GmbH. This is an open access article under the terms of the Creative Commons Attribution License, which permits use, distribution and reproduction in any medium, provided the original work is properly cited.

# Table of Contents

1. General information ........ S2
2. Reaction calorimetry experiments ........ S2
  2.1. Titration tests at different temperatures ........ S2
  2.2. Scan test ........ S3
  2.3. Titration test at 80 °C for reaction heat determination ........ S4
  2.4. Iodometric peroxide determination ........ S4
3. Description of continuous flow experiments ........ S5
  3.1. Oxidative esterifications with $H_2O_2$/HCl system ........ S5
  3.2. Oxidative esterifications in the presence of various solid acids and oxidants ........ S6
  3.3. Oxidative esterifications with *in situ*-generated $H_2SO_5$ ........ S6
    3.3.1 Parameter screening with hydrocinnamaldehyde ........ S6
    3.3.2 Additional parameter screening with benzaldehyde ........ S7
    3.3.3 Investigation of the reaction scope ........ S9
    3.3.4 Gram-scale synthesis of a key (–)-paroxetine intermediate ........ S9
    3.3.5 Calculation of green metrics ........ S10
4. Characterization data ........ S12
5. Collection of NMR spectra ........ S19
6. References ........ S58

# 1. General information

All solvents and chemicals were obtained from typical commercial vendors and were used as received, without any further purification.

Column chromatographic purification was performed by using a Biotage Isolera automated flash chromatography system with cartridges packed with KP-SIL, 60 Å (32–63 µm particle size). Analytical thin-layer chromatography (TLC) was carried out using Merck silica gel 60 GF254 plates. Compounds were visualized by means of UV or by using $KMnO_4$.

$^1H$- and $^{13}C$-NMR spectra were recorded on a Bruker Avance III 300 MHz instrument at room temperature, in $CDCl_3$ as solvent, at 300 MHz and 75 MHz, respectively. Chemical shifts (δ) are reported in ppm using TMS as internal standard. Coupling constants are given in Hz units.

Analytical HPLC measurements were carried out on a C18 reversed-phase column (150 × 4.6 mm, particle size 5 mm) at 37 °C using mobile phases A [$H_2O/CH_3CN$ 90:10 (v/v) + 0.1% TFA] and B ($CH_3CN$ + 0.1% TFA) at a flow rate of 1.5 mL $min^{-1}$. The gradient applied was as follows: linear increase from 30% solution B to 100% B in 8 min, hold at 100% solution B for 2 min.

GC-FID analysis was performed on a Shimadzu GCFID 2030 instrument equipped with a flame ionization detector, using an RTX-5MS column (30 m × 0.25 mm ID × 0.25 µm) and helium as carrier gas (40 cm $s^{-1}$ linear velocity). The injector temperature was set to 280 °C. After 1 min at 50 °C, the temperature was increased by 25 °C $min^{-1}$ to reach 300 °C and then kept constant at 300 °C for 3 min. The detector gases used for flame ionization were hydrogen and synthetic air (5.0 purity).

Optical rotation was measured in $CHCl_3$ (HPLC-grade) at 25 °C against the sodium D-line (λ= 589 nm) on a Perkin Elmer Polarimeter 341 using a 10-cm pathlength cell.

Equipment for the continuous flow reactions was assembled using commercially available components. Liquid streams were pumped by using Syrris® Asia syringe pumps. Flow systems were pressurized by using an adjustable backpressure regulator (BPR) from Zaiput. Reaction coils were heated by means of a conventional oil bath. Reagent feeds were either streamed directly or by using injection valves and sample loops. Sample loops and reactor coils were made by using perfluoroalkoxy alkane (PFA) tubings (1/16" OD, 0.80 mm ID).

A micro reaction calorimeter (µRC) from Thermal Hazard Technology was used to study the thermal behavior of the $H_2SO_4$–$H_2O_2$ reaction system.

Quantitative green metrics were calculated according to the literature.[S1]

CAUTION: Sulfuric acid is highly corrosive causing rapid tissue destruction and serious chemical burns. Persulfuric acid is one of the strongest oxidants known. It is unstable and potentially explosive, especially in mixtures with organic substances. Extreme care must therefore be taken when handling these substances! All equipment must be set up in a well-ventilated fume hood and personal protective equipment must be worn during experimentation. A thorough safety assessment should be made before conducting any experiments.

# 2. Reaction calorimetry experiments

## *2.1. Titration tests at different temperatures*

*Solution preparation:*

1.0 M $H_2O_2$: 0.486 g 35 wt% aq. $H_2O_2$ was diluted to 5 mL with MeOH or *i*PrOH.
1.0 M $H_2SO_4$: 0.516 g 95% $H_2SO_4$ was diluted to 5 mL with MeOH or *i*PrOH.

NOTE: these concentrations were different from the ones used for the flow reactions.

*Procedure:*

The titration mode was selected and the official procedures for the calorimeter were followed. MeOH was used as the solvent for titration at 25 °C, 50 °C and 70 °C while *i*PrOH was used for titration at 80 °C. 300 µL $H_2SO_4$

solution was added to the sample vial and reference vial, respectively. A stirring bar was placed into the sample vial and the stirring speed was set to 200 rpm. $H_2O_2$ solution was loaded into a 100 µL syringe and used as titrant. After the baseline stabilized, 10 µL $H_2O_2$ was injected, and the power was recorded. The injection was repeated 9 times and the interval time between two injections was specified as 300 s. MeOH/*i*PrOH was also used as titrant to demonstrate the mixing process.

*Results:*

For the titrations at 25 °C, 50 °C and 70 °C, the detected heat was the same for the two processes, which meant that the reaction between $H_2O_2$ and $H_2SO_4$ did not happen and only the mixing heat was recorded. For the titration at 80 °C, the heat was much larger when $H_2O_2$ was used as titrant. This indicated the formation of persulfuric acid.

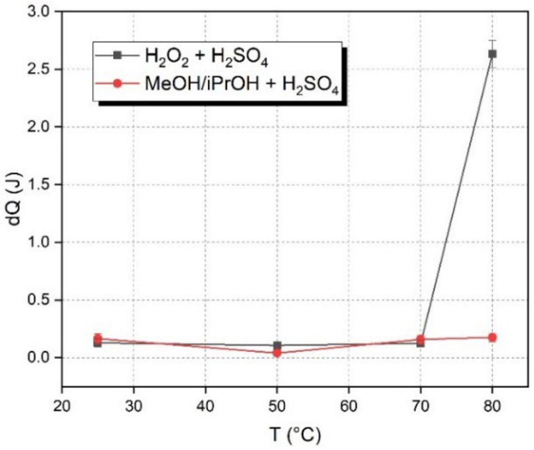


**Figure S1.** Detected heat of titrations at different temperatures.

## 2.2. Scan test

*Solution preparation:*

1.70 M $H_2O_2$: 0.826 g 35 wt% aq. $H_2O_2$ was diluted to 5 mL with *i*PrOH.
3.40 M $H_2SO_4$: 1.755 g 95% $H_2SO_4$ was diluted to 5 mL with *i*PrOH.

*Procedure:*

The scan mode was selected. 50 µL $H_2O_2$ solution and 50 µL $H_2SO_4$ solution were premixed in the sample vial at room temperature. To the reference vial, 100 µL *i*PrOH was added. A stirring bar was placed into both vials and the stirring speed was set to 200 rpm. Then the two vials were placed into the calorimeter. After the baseline stabilized, the sample was scanned from 25 °C to 150 °C at 1 °C min$^{-1}$ and held at 150 °C for 30 min.

*Results:*

Only one exothermic peak, which started from around 75 °C, was observed during the scan test. As the formation of persulfuric acid cannot happen at room temperature, this peak undoubtedly included the formation heat.

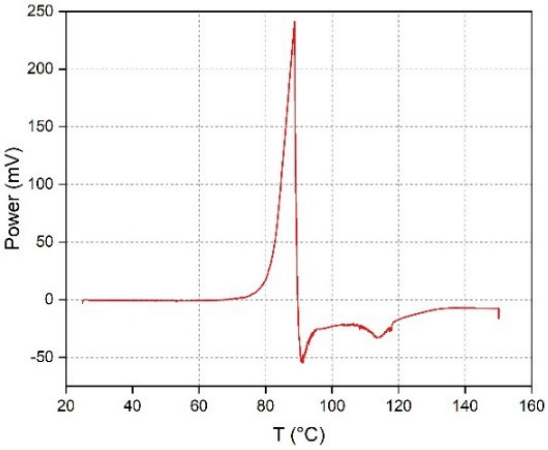


**Figure S2.** Sample scan from 25 °C to 150 °C at 1°C min$^{-1}$.

## 2.3. Titration test at 80 °C for reaction heat determination

*Solution preparation:*

8.50 M $H_2O_2$: 4.130 g 35 wt% aq. $H_2O_2$ was diluted to 5 mL with *i*PrOH.
1.89 M $H_2SO_4$: 0.976 g 95% $H_2SO_4$ was diluted to 5 mL with *i*PrOH.

*Procedure:*

The titration mode was selected, and the temperature was set to 80 °C. For the reaction, either 180, 270 or 360 μL $H_2SO_4$ solution was added to the sample vial and reference vial, respectively. A stirring bar was placed into the sample vials and the stirring speed was set to 200 rpm. $H_2O_2$ solution was loaded into a 100 μL syringe and used as titrant. After the baseline stabilized, either 20, 30, or 40 μL $H_2O_2$ was injected at once and the power was recorded. For each condition, the experiment was repeated twice in order to check the consistency. As $H_2SO_4$ was in excess, the calculation of reaction enthalpy was based on $H_2O_2$. For the mixing test, $H_2O$ was used as the titrant. For the blank test, *i*PrOH was used as a replacement for $H_2SO_4$.

NOTE: The long-term exposure to high temperature does slowly cause damage to the syringe.

*Results:*

**Table S1.** Summary of reaction enthalpy.

| Entry | Substrate | Titrant | dQ (J) | ΔHr (kJ $mol^{-1}$) | Ave. ± SD (kJ $mol^{-1}$) |
|---|---|---|---|---|---|
| 1 | 180 μL $H_2SO_4$ | 20 μL $H_2O_2$ | 47.2 | 266.5 | 271.5 ± 10.1 |
| 2 | | 20 μL $H_2O$ | 1.4 | / | |
| 3 | 270 μL $H_2SO_4$ | 30 μL $H_2O_2$ | 71.0 | 264.9 | |
| 4 | | 30 μL $H_2O$ | 2.7 | / | |
| 5 | 360 μL $H_2SO_4$ | 40 μL $H_2O_2$ | 99.8 | 283.2 | |
| 6 | | 40 μL $H_2O$ | 3.7 | / | |

## 2.4. Iodometric peroxide determination

Total peroxide concentration was measured by iodometric titration. This method was based on two major steps, as follows.

$$H_2O_2 + 2KI + H_2SO_4 \rightarrow I_2 + K_2SO_4 + 2H_2O$$

$$I_2 + 2Na_2S_2O_3 \rightarrow Na_2S_4O_6 + 2NaI$$

*Solution preparation:*

2 wt% KI in $H_2O$: 2 g KI was dissolved and diluted with $H_2O$ to 100 mL.
$(NH_4)_6Mo_7O_{24}$×$4H_2O$ solution: 9 g $(NH_4)_6Mo_7O24$×$4H_2O$ was dissolved in 10 mL 25 wt% $NH_4OH$ in $H_2O$. 24 g $NH_4NO_3$ was added, and the solution was diluted to 100 mL with $H_2O$.
Diluted $H_2SO_4$: 20 mL concentrated $H_2SO_4$ was diluted to 100 mL with $H_2O$.
0.1 M $Na_2S_2O_3$: 2.493 g $Na_2S_2O_3$×$5H_2O$ was dissolved and diluted to 100 mL with $H_2O$.
1 wt% starch indicator: 0.1 g soluble starch was added to 100 mL hot $H_2O$.

*Procedure:*

$H_2O_2$ (1.70 M) and $H_2SO_4$ (3.40 M) solutions in the batch calorimetry scan test were used to prepare the reaction mixture. The scan mode of the calorimeter was adopted to provide the temperature for the formation of persulfuric acid. 100 μL of reaction solution was diluted with 1 mL of $H_2O$. 200 μL of diluted $H_2SO_4$ solution, then 200 μL of the KI solution were added. One drop of the $(NH_4)_6Mo_7O_{24}$ solution was added. Titration with thiosulfate (0.1 M) was performed until a pale-yellow color was reached. 20 μL of starch indicator solution was added. Further titration with 0.1 M thiosulfate was performed until a colorless solution was observed.

*Results:*

**Table S2.** Summary of iodometric titrations.

| Entry | Mixture | Scan process of the calorimeter | $n_{(H2O2)}$ (mmol) |
|---|---|---|---|
| 1[a] | 50 µL $H_2O_2$ + 50 µL *i*PrOH | None | 0.04900 |
| 2[a] | 50 µL $H_2O_2$ + 50 µL $H_2SO_4$ | Scan from 25 °C to 60 °C at 1 °C min$^{-1}$, held for 10 min | 0.04650 |
| 3[b] | 50 µL $H_2O_2$ + 50 µL $H_2SO_4$ | Scan from 60 °C to 80 °C at 1 °C min$^{-1}$, held for 40 min | 0.00075 |
| 4[a,c] | 50 µL $H_2O_2$ + 50 µL *i*PrOH | Scan from 60 °C to 80 °C at 1 °C min$^{-1}$, held for 40 min | 0.04710 |

[a]Persulfuric acid was not formed. [b]Persulfuric acid was formed. [c]Blank test to prove $H_2O_2$ did not evaporate or decompose.

## 3. Description of continuous flow experiments

### *3.1. Oxidative esterifications with $H_2O_2$/HCl system*

Substrate solution containing 1 equiv. of hydrocinnamaldehyde and 1.1–3.0 equiv. of HCl (added as 6 M aq. solution) in MeOH and a solution of $H_2O_2$ (prepared from 35 wt% aq. solution) in MeOH were pumped as separate streams (P1 and P2) by using Syrris® Asia syringe pumps equipped with two injection valves and two sample loops (5 or 8 mL, each). With MeOH serving as carrier solvent, the feeds were combined in a Y-mixer. The resulting stream was directed through a 15-mL reaction coil (15–60 min residence time) which was heated at 50, 100 or 120 °C and pressurized at 5 bar. The reactor outlet was directed into a flask containing a stirred mixture of saturated aq. $NaHCO_3$ and some $MnO_2$ in order to quench any excess acid and/or oxidant. After reaching steady state, approx. 20 µL aliquots of crude material were collected. The samples were diluted with 1 mL of $CH_3CN/H_2O$ 9:1 and was analyzed by analytical HPLC directly after the flow experiments.

**Table S3.** Oxidative esterifications with $H_2O_2$/HCl system.

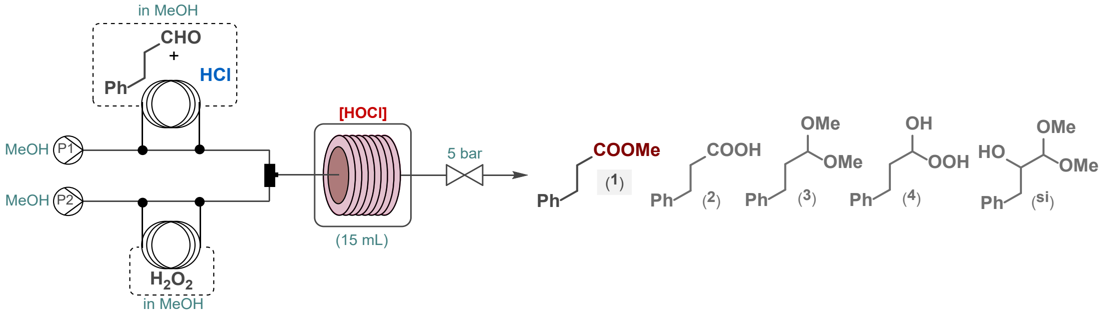


| # | $c_{substr.}$ (M) | HCl[a] (equiv.) | $H_2O_2$[b] (equiv.) | *T* (°C) | Flow rates (µL min$^{-1}$) | $t_r$ (min) | Conv. (%)[c] | Chemoselectivity (%)[c] | | | | | |
|---|---|---|---|---|---|---|---|---|---|---|---|---|---|
| | | | | | | | | **1** | **2** | **3** | **4** | **si** | Unid.[d] |
| 1 | 0.25 | 1.1 | 1.1 | 100 | 2 × 250 | 30 | 100 | 42 | 0 | 14 | 0 | 44 | 0 |
| 2 | 0.25 | 1.5 | 1.5 | 100 | 2 × 250 | 30 | 100 | 47 | 0 | 3 | 0 | 50 | 0 |
| 3 | 0.25 | 3.0 | 3.0 | 100 | 2 × 250 | 30 | 100 | 55 | 4 | 0 | 0 | 41 | 0 |
| 4 | 0.25 | 2.0 | 3.0 | 100 | 2 × 250 | 30 | 100 | 54 | 4 | 0 | 0 | 42 | 0 |
| 5 | 0.25 | 3.0 | 2.0 | 100 | 2 × 250 | 30 | 100 | 54 | 3 | 0 | 0 | 43 | 0 |
| 6 | 0.25 | 2.0 | 2.0 | 50 | 2 × 250 | 30 | 48 | 38 | 0 | 62 | 0 | 0 | 0 |
| 7 | 0.25 | 2.0 | 2.0 | 50 | 2 × 125 | 60 | 59 | 54 | 0 | 46 | 0 | 0 | 0 |
| 8 | 0.25 | 2.0 | 2.0 | 100 | 2 × 500 | 15 | 100 | 53 | 0 | 3 | 0 | 44 | 0 |
| 9 | 0.25 | 2.0 | 2.0 | 120 | 2 × 250 | 20[e] | 100 | 36 | 0 | 3 | 0 | 55 | 6 |
| 10 | 0.75 | 2.0 | 2.0 | 100 | 2 × 250 | 30 | 100 | 58 | 8 | 4 | 0 | 24 | 6 |

[a]Stock solution was made using 6 M aq. HCl. [b]Stock solution was made using 35 wt% aq. $H_2O_2$. [c]Determined by HPLC area%. [d]Unidentified side products. [e]Gas formation occurred in the reaction coil.

In order to assign HPLC signals of side product **3** and **4**, reference samples were prepared according to a literature procedure.[S2] Hydrocinnamic acid, **2** is commercially available. Side product **si** was isolated chromatographically and characterized by $^1$H NMR. NMR data of the compound matched the reported literature.[S3]

## 3.2. *Oxidative esterifications in the presence of various solid acids and oxidants*

Substrate solution containing 0.5 M of hydrocinnamaldehyde in MeOH and a solution of UHP or TBHP or $H_2O_2$ (TBHP was added as 5.5 M decane solution, $H_2O_2$ was added as 35 wt% aq. solution) in MeOH were pumped as separate streams (P1 and P2) by using Syrris® Asia syringe pumps equipped with two injection valves and two sample loops (6 mL, each). With MeOH serving as carrier solvent, the feeds were combined in a Y-mixer. The resulting stream was directed through an Omnifit® glass column (10 mm ID, adjustable height) containing 1.5 g of Amberlyst 15 or 5.0 g of Dowex 50WX8 as solid acid. The column was heated at 80 or 100 °C by using a Syrris® Asia column heater and was pressurized at 5 bar. The reactor outlet was directed into a flask containing a stirred mixture of some $MnO_2$ in MeOH in order to quench any excess oxidant. After reaching steady state, approx. 20 µL aliquots of crude material were collected. The samples were diluted with 1 mL of $CH_3CN/H_2O$ 9:1 and was analyzed by analytical HPLC directly after the flow experiments.

**Table S4.** Oxidative esterifications in the presence of various solid acids and oxidants.

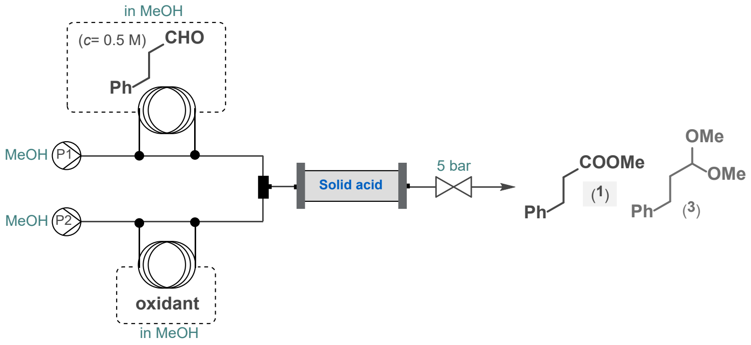


| # | Solid acid | Oxidant | | | Flow rates (µL min$^{-1}$) | | $T$ (°C) | $t_r$ (min) | Conv. (%)[d] | Chemoselectivity (%)[d] | | |
|---|---|---|---|---|---|---|---|---|---|---|---|---|
| | | Name | $c_{stock. sol.}$ (M) | (equiv.) | P1 | P2 | | | | **1** | **3** | Unid.[e] |
| 1 | Amberlyst 15 | UHP | 0.5 | 1.5 | 80 | 120 | 80 | 10 | 68 | 52 | 48 | 0 |
| 2 | Dowex 50WX8 | UHP | 0.5 | 1.5 | 80 | 120 | 80 | 20 | 92 | 85 | 15 | 0 |
| 3 | Dowex 50WX8 | TBHP | 0.5[a] | 1.5 | 80 | 120 | 80 | 20 | 100 | 4 | 62 | 34 |
| 4 | Dowex 50WX8 | $H_2O_2$ | 0.5[b] | 1.5 | 80 | 120 | 80 | 20 | 94 | 86 | 14 | 0 |
| 5 | Dowex 50WX8 | $H_2O_2$ | 11.6[c] | 1.5 | 188 | 12 | 80 | 20 | 80 | 73 | 17 | 0 |
| 6 | Dowex 50WX8 | $H_2O_2$ | 0.5[b] | 1.5 | 80 | 120 | 100 | 20 | 95 | 88 | 12 | 0 |
| 7 | Dowex 50WX8 | $H_2O_2$ | 1.5[b] | 3.0 | 100 | 100 | 100 | 20 | 96 | 96 | 4 | 0 |
| 8 | Dowex 50WX8 | - | | - | 200 | 0 | 80 | 20 | 90 | 0 | 100 | 0 |

[a]Stock solution was made using 5.5 M TBHP in decane. [b]Stock solution was made using 35 wt% aq. $H_2O_2$. [c]35 wt% aq. $H_2O_2$ was pumped. [d]Determined by HPLC area%. [e]Unidentified side products.

## 3.3. *Oxidative esterifications with in situ-generated $H_2SO_5$*

### 3.3.1 *Parameter screening with hydrocinnamaldehyde*

Substrate solution containing 1.0, 2.0 or 3.0 M of hydrocinnamaldehyde and a 4.0 or 8.0 M solution of $H_2SO_4$ (prepared from cc $H_2SO_4$) were pumped as separate streams (P1 and P2) by using Syrris® Asia syringe pumps equipped with two injection valves and two sample loops (3, 5 or 10 mL, each). As a third stream, a 2.0 M solution of $H_2O_2$ in MeOH or 35 or 50 wt% aq. $H_2O_2$ solution (11.6 or 17.6 M) or 2.0 M UHP solution in MeOH or 2.0 M

TBHP solution in MeOH (added as 5.5 M decane solution) was pumped by using a Syrris® Asia syringe pump (P3) equipped with an injection valve and a sample loop (3 or 6 mL). With MeOH serving as carrier solvent, the substrate and $H_2SO_4$ feeds were combined in a Y-mixer, then the resulting stream was mixed up with the $H_2O_2$ feed through a second Y-mixer. The combined liquid stream was next directed through a heated reaction coil (15 or 1.5 mL, 1–20 min residence time) which was pressurized at 5 bar. The reactor outlet was directed into a flask containing a stirred mixture of saturated aq. $NaHCO_3$ and some $MnO_2$ in order to quench any excess acid and/or oxidant. After reaching steady state, approx. 20 µL aliquots of crude material were collected. The samples were diluted with 1 mL of $CH_3CN/H_2O$ 9:1 and was analyzed by analytical HPLC directly after the flow experiments.

See Table 1 in the manuscript for the corresponding reaction data and also Scheme 1 for the setup.

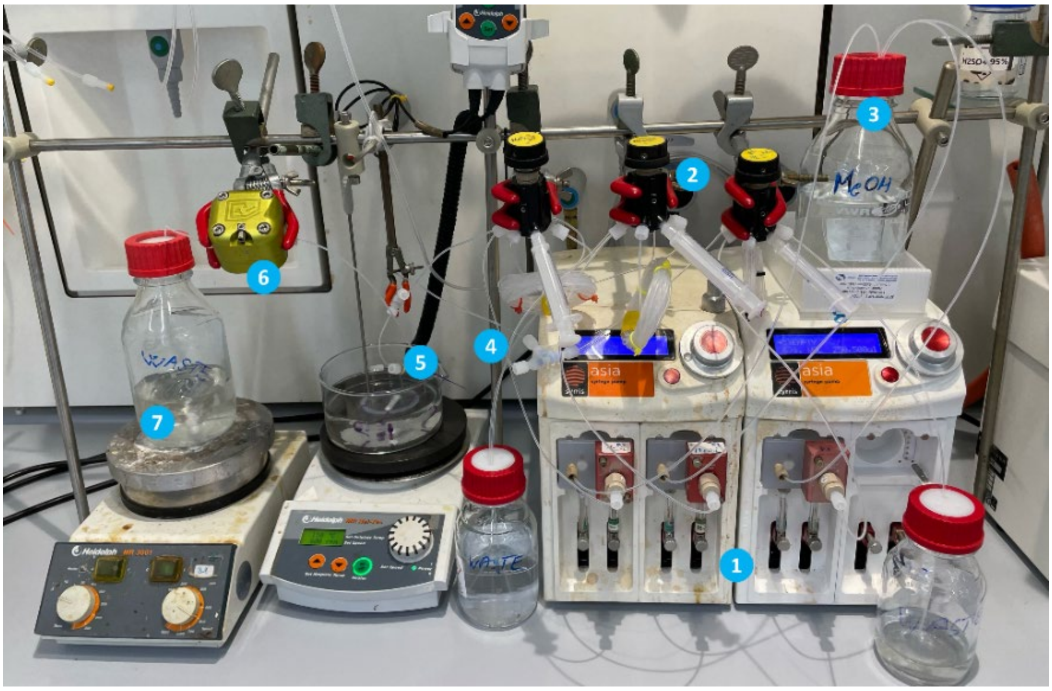


**Figure S3.** Photograph of the flow setup. Legend: (1) Syringe pumps (P1, P2 and P3), (2) reagent feeds with injection valves and two sample loops, (3) carrier solvent, (4) Y-mixers, (5) reaction coil in a heated oil bath, (6) adjustable BPR, (7) reactor outlet.

**Table S5.** Flow rates for Table 1 in the manuscript.

| Entry in Table 1 | Flow rates (µL min$^{-1}$) | | |
|---|---|---|---|
| | P1 | P2 | P3 |
| 1 | 187 | 187 | 375 |
| 2 | 300 | 150 | 300 |
| 3 | 395 | 119 | 237 |
| 4 | 790 | 237 | 474 |
| 5 | 395 | 237 | 119 |
| 6 | 300 | 270 | 180 |
| 7 | 242 | 73 | 436 |
| 8 | 599 | 90 | 62 |
| 9 | 535 | 161 | 55 |
| 10 | 535 | 161 | 55 |
| 11 | 415 | 249 | 86 |
| 12 | 339 | 305 | 105 |
| 13 | 1069 | 321 | 110 |
| 14 | 548 | 165 | 38 |
| 15 | 394 | 119 | 237 |
| 16 | 680 | 0 | 70 |

### *3.3.2 Additional parameter screening with benzaldehyde*

Substrate solution containing 1.0 or 0.5 M of benzaldehyde and a 4.0 or 8.0 M solution of $H_2SO_4$ (prepared from cc $H_2SO_4$) were pumped as separate streams (P1 and P2) by using Syrris® Asia syringe pumps equipped with

two injection valves and two sample loops (5 or 10 mL, each). As a third stream, 35 wt% aq. $H_2O_2$ solution (11.6 M) or 2.0 M UHP solution in MeOH was pumped by using a Syrris® Asia syringe pump (P3) equipped with an injection valve and a sample loop (3 or 5 mL). With MeOH serving as carrier solvent, the substrate and $H_2SO_4$ feeds were combined in a Y-mixer, then the resulting stream was mixed up with the oxidant feed through a second Y-mixer. The combined liquid stream was next directed through a 1.5-mL reaction coil (1, 2 or 4 min residence time) which was heated at 80–140 °C and pressurized at 5 bar. The reactor outlet was directed into a flask containing a stirred mixture of saturated aq. $NaHCO_3$ and some $MnO_2$ in order to quench any excess acid and/or oxidant. The reactor outlet was directed into a flask containing a stirred mixture of saturated aq. $NaHCO_3$ and some $MnO_2$ in order to quench any excess acid and/or oxidant. After reaching steady state, approx. 20 µL aliquots of crude material were collected. The samples were diluted with 1 mL of $CH_3CN/H_2O$ 9:1 and was analyzed by analytical HPLC directly after the flow experiments.

**Table S6.** Results of parameter screening with benzaldehyde.

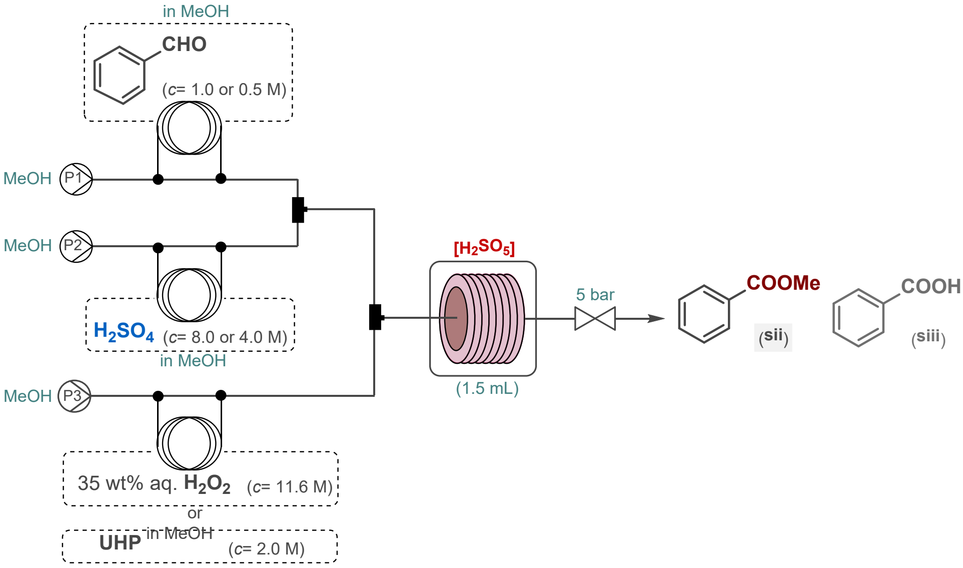


| #[a] | $c_{substr.}$ (M) | $c_{H2SO4}$ (M) | Oxidant | Flow rates (µL min$^{-1}$) | | | $H_2SO_4$ (equiv.) | $H_2O_2$ (equiv.) | $t_r$ (min) | *T* (°C) | Conv. (%)[b] | Chemoselectivity (%)[b] | | |
|---|---|---|---|---|---|---|---|---|---|---|---|---|---|---|
| | | | | P1 | P2 | P3 | | | | | | **sii** | **siii** | Unid.[c] |
| 1 | 1 | 8 | $H_2O_2$ | 534 | 161 | 56 | 2.4 | 1.2 | 2 | 120 | 100 | 83 | 17 | 0 |
| 2 | 1 | 8 | $H_2O_2$ | 534 | 161 | 56 | 2.4 | 1.2 | 2 | 80 | 89 | 82 | 11 | 7 |
| 3 | 1 | 8 | $H_2O_2$ | 1068 | 322 | 112 | 2.4 | 1.2 | 1 | 120 | 99 | 84 | 16 | 0 |
| 4 | 1 | 8 | Hó | 267 | 81 | 28 | 2.4 | 1.2 | 4 | 120 | 100 | 81 | 19 | 0 |
| 5 | 1 | 8 | $H_2O_2$ | 534 | 161 | 56 | 2.4 | 1.2 | 2 | 140 | 95 | 79 | 21 | 0 |
| 6 | 1 | 8 | $H_2O_2$ | 466 | 211 | 73 | 3.6 | 1.8 | 2 | 120 | 100 | 82 | 18 | 0 |
| 7 | 1 | 8 | $H_2O_2$ | 466 | 211 | 73 | 3.6 | 1.8 | 2 | 90 | 99 | 88 | 12 | 0 |
| 8 | 1 | 8 | $H_2O_2$ | 425 | 192 | 133 | 3.6 | 3.6 | 2 | 90 | 98 | 63 | 22 | 15 |
| 9 | 1 | 8 | $H_2O_2$ | 563 | 128 | 59 | 1.8 | 1.2 | 2 | 120 | 100 | 85 | 15 | 0 |
| 10 | 1 | 8 | $H_2O_2$ | 531 | 150 | 70 | 2.25 | 1.5 | 2 | 100 | 97 | 87 | 13 | 0 |
| 11 | 1 | 8 | UHP | 394 | 119 | 237 | 2.4 | 1.2 | 2 | 120 | 99 | 91 | 9 | 0 |
| 12 | 1 | 8 | UHP | 410 | 93 | 247 | 1.8 | 1.2 | 2 | 120 | 97 | 91 | 9 | 0 |
| 13 | 1 | 8 | UHP | 319 | 144 | 287 | 3.6 | 1.8 | 2 | 100 | 98 | 91 | 9 | 0 |
| 14 | 0.5 | 4 | UHP | 468 | 141 | 141 | 2.4 | 1.2 | 2 | 120 | 95 | 92 | 8 | 0 |
| 15 | 0.5 | 8 | UHP | 516 | 78 | 156 | 2.4 | 1.2 | 2 | 120 | 93 | 92 | 8 | 0 |

[a]$H_2SO_4$ stock solutions were made using cc. $H_2SO_4$. [b]Determined by HPLC area%. [c]Unidentified side products.

### 3.3.3 Investigation of the reaction scope

Substrate solution containing 1.0 or 0.5 M of the corresponding aldehyde and a 4.0 or 8.0 M solution of $H_2SO_4$ (prepared from cc $H_2SO_4$) were pumped as separate streams (P1 and P2) by using Syrris® Asia syringe pumps equipped with two injection valves and two sample loops (10 mL, each). As a third stream, 35 wt% aq. $H_2O_2$ solution (11.6 M) or 2.0 M UHP solution in MeOH was pumped by using a Syrris® Asia syringe pump (P3) equipped with an injection valve and a sample loop (3 or 6 mL). With MeOH serving as carrier solvent, the substrate and $H_2SO_4$ feeds were combined in a Y-mixer, then the resulting stream was mixed up with the oxidant feed through a second Y-mixer. (For some of the reactions, EtOH or *i*PrOH was used as solvent and also as carrier solvent.) The combined liquid stream was next directed through a 1.5-mL reaction coil (2 or 4 min residence time) which was heated at 120 °C and pressurized at 5 bar. The reactor outlet was directed into a flask containing a stirred mixture of saturated aq. $NaHCO_3$ and some $MnO_2$ in order to quench any excess acid and/or oxidant. After reaching steady state, the crude reactor outlet was collected for 2–10 min. A 20 µL aliquot of the crude material was diluted with 1 mL of $CH_3CN/H_2O$ 9:1 and was immediately analyzed by analytical HPLC or GC-FID. The collected mixture was extracted with $CH_2Cl_2$, washed with brine and dried over $MgSO_4$. After evaporation, the obtained material was analyzed by $^1H$ and $^{13}C$ NMR spectroscopy. When necessary, column chromatographic purification was performed using mixtures of cyclohexane and EtOAc as eluent.

See Tables 2 and 3 in the manuscript for the corresponding reaction data and also for representation of the setup.

**Table S7.** Flow rates for Table 3 in the manuscript.

| Entry in Table 3 | Flow rates (µL min$^{-1}$) | | |
|---|---|---|---|
| | P1 | P2 | P3 |
| 1 | 534 | 161 | 56 |
| 2 | 394 | 119 | 237 |
| 3 | 534 | 161 | 56 |
| 4 | 534 | 161 | 56 |
| 5 | 534 | 161 | 56 |
| 6 | 534 | 161 | 56 |
| 7 | 534 | 161 | 56 |
| 8 | 534 | 161 | 56 |
| 9 | 197 | 60 | 119 |
| 10 | 394 | 119 | 237 |
| 11 | 394 | 119 | 237 |
| 12 | 394 | 119 | 237 |
| 13 | 234 | 71 | 71 |
| 14 | 394 | 119 | 237 |
| 15 | 197 | 60 | 119 |
| 16 | 394 | 119 | 237 |
| 17 | 234 | 71 | 71 |
| 18 | 468 | 141 | 141 |
| 19 | 534 | 161 | 56 |
| 20 | 534 | 161 | 56 |
| 21 | 394 | 119 | 237 |

### 3.3.4 Gram-scale synthesis of a key (–)-paroxetine intermediate

Preparation of γ-nitroaldehyde **5**:

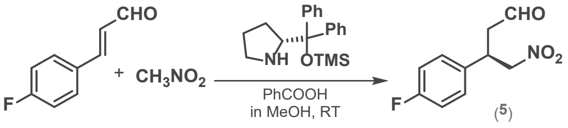


**Scheme S1.** Synthesis of **5**.

A mixture containing 4-fluorocinnamaldehyde (1 equiv., 0.5 M), nitromethane (3 equiv.), AcOH (0.3 equiv.) and 10 mol% of (*R*)-α,α-diphenyl-2-pyrrolidinemethanol trimethylsilyl ether as organocatalyst was stirred for 24 h at RT in MeOH as solvent. After then, the mixture was filtrated through a pad of celite, washed with $CH_2Cl_2$ and evaporated. The crude product was purified by means of column chromatography using a mixture of ethyl acetate/40-60 petroleum ether as eluent.

NMR data of the isolated compound matched the reported literature.[S4] $[\alpha]_D^{25}$= +15.8 (c= 1.0, $CHCl_3$).

Continuous flow oxidative esterification of **5**:

Substrate solution containing 1.0 M of γ-nitroaldehyde **5** and an 8.0 M solution of $H_2SO_4$ (prepared from cc $H_2SO_4$) were pumped directly as separate streams (P1 and P2) by using Syrris® Asia syringe pumps. As a third stream, 35 wt% aq. $H_2O_2$ solution (11.6 M) in MeOH was pumped by using a Syrris® Asia syringe pump (P3) equipped with an injection valve and a sample loop (15 mL). With MeOH serving as carrier solvent for the $H_2O_2$ stream, the substrate and $H_2SO_4$ feeds were combined in a Y-mixer, then the resulting stream was mixed up with the oxidant feed through a second Y-mixer. The combined liquid stream was next directed through a 1.5-mL reaction coil (2 min residence time) which was heated at 120 °C and pressurized at 5 bar. The reactor outlet was directed into a flask containing a stirred mixture of saturated aq. $NaHCO_3$ and some $MnO_2$ in order to quench any excess acid and/or oxidant. After reaching steady state, the crude reactor outlet was collected for 45 min. 20 μL aliquots of the crude material was diluted with 1 mL of $CH_3CN/H_2O$ 9:1 and was immediately analyzed by analytical HPLC. The collected mixture was extracted with $CH_2Cl_2$, washed with brine and dried over $MgSO_4$. The obtained material was analyzed by $^1H$ and $^{13}C$ NMR spectroscopy.

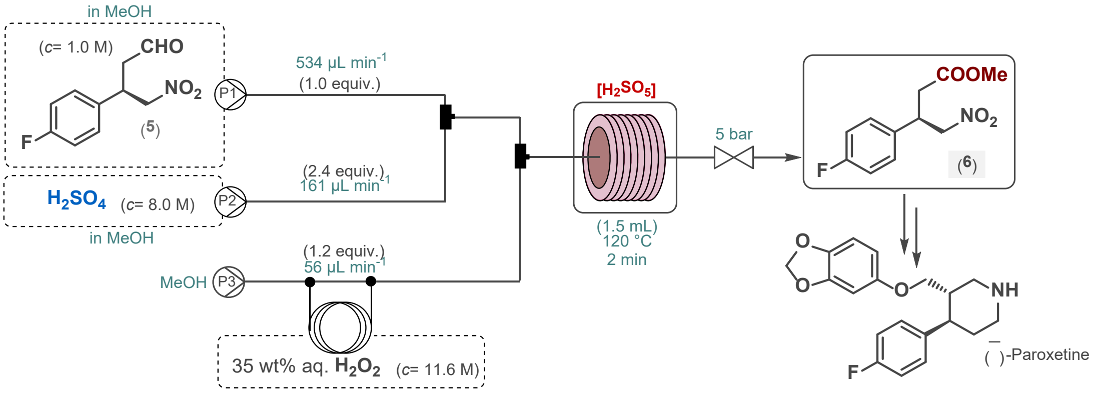


**Scheme S2.** Flow synthesis of **6**.

### 3.3.5 Calculation of green metrics

E factor, process mass intensity (PMI), reaction mass efficiency (RME), atom economy (AE) and optimum efficiency (OE) were calculated using the following equations.[S1]

$$E\ factor = \frac{total\ mass\ of\ waste}{mass\ of\ product}$$

$$PMI = \frac{total\ mass\ of\ raw\ materials\ used}{mass\ of\ product}$$

$$RME = \frac{mass\ of\ isolated\ product}{total\ mass\ of\ reactants} \times 100$$

$$AE = \frac{molecular\ weight\ of\ product}{total\ molecular\ weight\ of\ reactants} \times 100$$

$$OE = \frac{RME}{AE} \times 100$$

Green metrics were calculated for the oxidative esterification of γ-nitroaldehyde **5** yielding γ-nitroester **6**. The *in situ*-generated persulfuric acid-mediated flow process (described in section 3.3.4 and also in the manuscript) was directly compared to a literature batch process utilizing *N*-bromosuccinimide (NBS)-based oxidation (Scheme S2). (See: K. L. Jensen, P. H. Poulsen, B. S. Donslund, F. Morana and K. A. Jørgensen, *Org. Lett.* **2012**, *14*, 1516-1519; *ref. 5d* in the manuscript.)

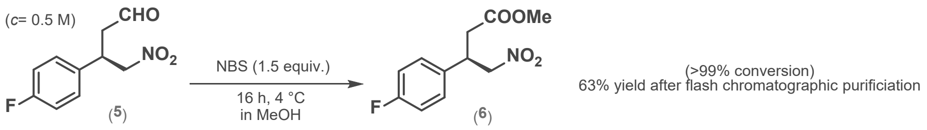


**Scheme S2.** NBS-mediated literature batch procedure for the oxidative esterification of **5**.

The NBS-mediated reaction was reported as a one-pot/two-step process. In the first step, Michael addition between 4-fluorocinnamaldehyde and nitromethane in the presence of a diphenylprolinol-type organocatalyst resulted γ-nitroaldehyde **5** (similarly to the reaction shown in Scheme 1). This step was reported to be quantitative and selective, thus calculation of the green metrics exclusively for the subsequent oxidative esterification step was possible.

In the NBS-mediated process, reaction conditions and yield were specified for the preparation of compound *ent*-**6** only ((*S*)-3-(4-fluorophenyl)-4-nitrobutanoate). The (*R*)-isomer (compound **6**) was prepared under identical conditions but using the opposite enantiomer of the chiral catalyst. For the calculation of green metrics, the same yield and reaction conditions were thus assumed for compound **6** as those reported for compound *ent*-**6**.

According to the NBS-mediated process, γ-nitroester **6** was isolated by means flash chromatographic purification. The exact amounts of solvents used for the purification was not reported by the authors. In case of the persulfuric acid-mediated flow process, **6** was obtained in a pure form after extractive work-up. In order that the data can be compared directly, chemicals and solvents used for work-up and purification have been excluded from the calculations.

For the calculation of the E factor, the mass of $H_2O$ was excluded. For the calculation of PMI, the total mass used for the calculation included $H_2O$ as well.

**Table S8.** Values used for the assessment of green metrics of the *in situ*-generated persulfuric acid-mediated continuous flow oxidative esterification of γ-nitroaldehyde **5**.

| Role | Chemical | Equiv. | *n* (mmol) | Mass (g) | *V* (mL) | Mw (g mol$^{-1}$) | *ρ* (g mL$^{-1}$) |
|---|---|---|---|---|---|---|---|
| REACTION | | | | | | | |
| Reactant | Compound **5** | 1.0 | 24.030 | 5.075 | | 211.190 | |
| Reactant | $H_2SO_4$ | 2.4 | 57.672 | 5.656 | 3.074 | 98.079 | 1.8400 |
| Reactant | $H_2O_2$ | 1.2 | 28.836 | 0.981 | | 34.015 | |
| Solvent | $H_2O$[a] | | | 1.822 | | | |
| Solvent | MeOH | | | 15.836<br>3.246 | 20.0[b]<br>4.1[c] | 32.040 | 0.7918 |
| PRODUCT | | | | | | | |
| Product | Compound **6** | 0.942 | 22.635 | 5.46 | | 241.22 | |

[a]$H_2O$ content of 35 wt% aq. $H_2O_2$ solution. ($H_2O$ content of cc. $H_2SO_4$ was omitted.) [b]MeOH used for the substrate solution, approximate volume. [c]MeOH used for the $H_2SO_4$ solution, approximate volume.

**Table S9.** Values used for the assessment of green metrics of the NBS-mediated literature batch process for the oxidative esterification of γ-nitroaldehyde **5**.

| Role | Chemical | Equiv. | *n* (mmol) | Mass (mg) | *V* (mL) | Mw (g mol$^{-1}$) | *ρ* (g mL$^{-1}$) |
|---|---|---|---|---|---|---|---|
| REACTION | | | | | | | |
| Reactant | Compound **5** | 1.0 | 0.2 | 42.238 | | 211.190 | |
| Reactant | NBS | 1.5 | 0.3 | 53.394 | | 177.980 | |
| Solvent | MeOH | | | 316.72 | 0.4 | 32.040 | 0.7918 |
| PRODUCT | | | | | | | |
| Product | Compound **6** | 0.63 | 0.126 | 30.394 | | 241.22 | |

# 4. Characterization data

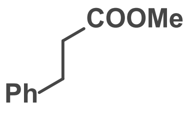


Methyl 3-phenylpropanoate

830.4 mg (95%) was isolated after extractive work-up.

$^{1}H$ and $^{13}C$ NMR data of the compound matches the reported literature.[S5]

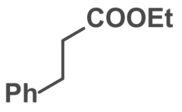


Ethyl 3-phenylpropanoate

$^{1}H$ and $^{13}C$ NMR data of the compound matches the reported literature.[S5]

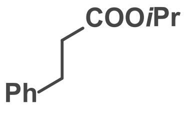


Ethyl 3-phenylpropanoate

$^{1}H$ and $^{13}C$ NMR data of the compound matches the reported literature.[S5]

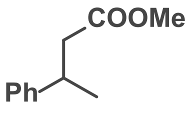


Methyl 3-phenylbutanoate

255.0 mg (89%) was isolated after extractive work-up.

$^{1}H$ and $^{13}C$ NMR data of the compound matches the reported literature.[S6]

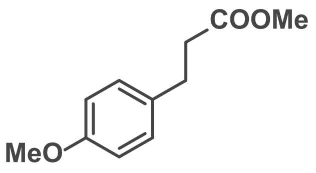


Methyl 3-(4-methoxyphenyl)propanoate

$^{1}H$ and $^{13}C$ NMR data of the compound matches the reported literature.[S7]

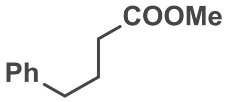

Methyl 4-phenylbutanoate

403.3 mg (85%) was isolated after column chromatographic purification (EtOAc/cyclohexane 0–50% as eluent).

$^{1}H$ and $^{13}C$ NMR data of the compound matches the reported literature.[S6]

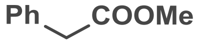

Methyl 2-phenylacetate

$^{1}H$ and $^{13}C$ NMR data of the compound matches the reported literature.[S8]

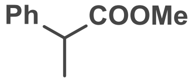

Methyl 2-phenylpropanoate

$^{1}H$ and $^{13}C$ NMR data of the compound matches the reported literature.[S9]

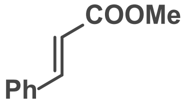

Methyl cinnamate

$^{1}H$ and $^{13}C$ NMR data of the compound matches the reported literature.[S8]

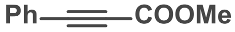

Methyl 3-phenylpropiolate

$^{1}H$ and $^{13}C$ NMR data of the compound matches the reported literature.[S10]

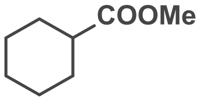

Methyl cyclohexanecarboxylate

$^{1}H$ and $^{13}C$ NMR data of the compound matches the reported literature.[S8]

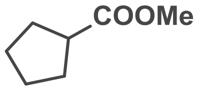

Methyl cyclopentanecarboxylate

$^{1}H$ and $^{13}C$ NMR data of the compound matches the reported literature.[S11]

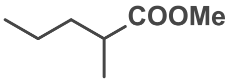

Methyl 2-methylpentanoate

$^{1}H$ and $^{13}C$ NMR data of the compound matches the reported literature.[S12]

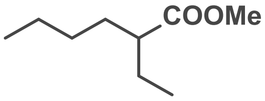

Methyl 2-ethylhexanoate

$^{1}H$ and $^{13}C$ NMR data of the compound matches the reported literature.[S13]

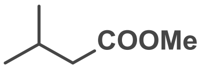

Methyl 3-methylbutanoate

301.2 mg (97%) was isolated after extractive work-up.

$^{1}H$ and $^{13}C$ NMR data of the compound matches the reported literature.[S14]

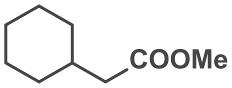

Methyl 2-cyclohexylacetate

$^{1}H$ and $^{13}C$ NMR data of the compound matches the reported literature.[S15]

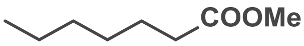

Methyl heptanoate

$^{1}H$ and $^{13}C$ NMR data of the compound matches the reported literature.[S16]

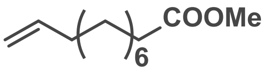

Methyl undec-10-enoate

$^{1}H$ and $^{13}C$ NMR data of the compound matches the reported literature.[S17]

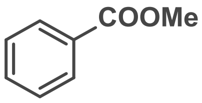

Methyl benzoate

234.2 mg (87%) was isolated after column chromatographic purification (EtOAc/cyclohexane 0–50% as eluent).

$^{1}H$ and $^{13}C$ NMR data of the compound matches the reported literature.[S8]

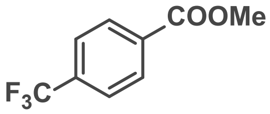


Methyl 4-(trifluoromethyl)benzoate

527.3 mg (97%) was isolated after extractive work-up.

$^{1}H$ and $^{13}C$ NMR data of the compound matches the reported literature.[S18]

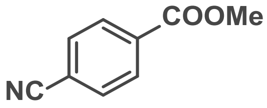


Methyl 4-cyanobenzoate

$^{1}H$ and $^{13}C$ NMR data of the compound matches the reported literature.[S8]

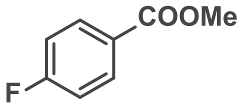


Methyl 4-fluorobenzoate

$^{1}H$ and $^{13}C$ NMR data of the compound matches the reported literature.[S18]

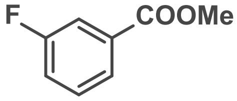


Methyl 3-fluorobenzoate

379.1 mg (92%) was isolated after extractive work-up.

$^{1}H$ and $^{13}C$ NMR data of the compound matches the reported literature.[S19]

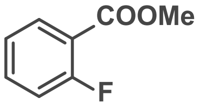


Methyl 2-fluorobenzoate

$^{1}H$ and $^{13}C$ NMR data of the compound matches the reported literature.[S20]

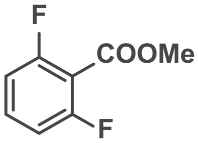


Methyl 2,6-difluorobenzoate

$^{1}H$ and $^{13}C$ NMR data of the compound matches the reported literature.[S21]

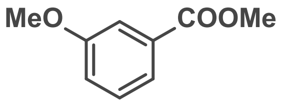

Methyl 3-methoxybenzoate

$^{1}H$ and $^{13}C$ NMR data of the compound matches the reported literature.[S18]

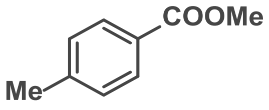

Methyl 4-methylbenzoate

$^{1}H$ and $^{13}C$ NMR data of the compound matches the reported literature.[S8]

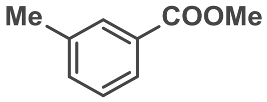

Methyl 3-methylbenzoate

$^{1}H$ and $^{13}C$ NMR data of the compound matches the reported literature.[S18]

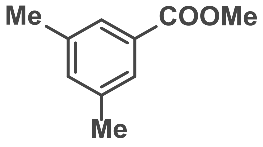

Methyl 3,5-dimethylbenzoate

$^{1}H$ and $^{13}C$ NMR data of the compound matches the reported literature.[S22]

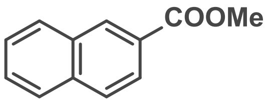

Methyl 2-naphthoate

$^{1}H$ and $^{13}C$ NMR data of the compound matches the reported literature.[S8]

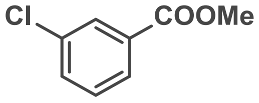

Methyl 3-chlorobenzoate

$^{1}H$ and $^{13}C$ NMR data of the compound matches the reported literature.[S23]

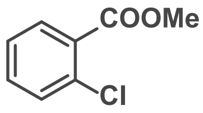


Methyl 2-chlorobenzoate

$^{1}H$ and $^{13}C$ NMR data of the compound matches the reported literature.[S24]

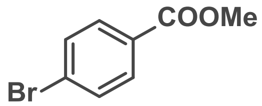


Methyl 4-bromobenzoate

376.5 mg (89%) was isolated after extractive work-up.

$^{1}H$ and $^{13}C$ NMR data of the compound matches the reported literature.[S8]

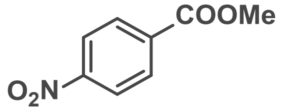


Methyl 4-nitrobenzoate

$^{1}H$ and $^{13}C$ NMR data of the compound matches the reported literature.[S8]

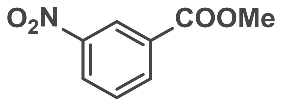


Methyl 3-nitrobenzoate

$^{1}H$ and $^{13}C$ NMR data of the compound matches the reported literature.[S25]

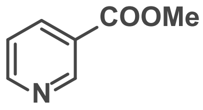


Methyl nicotinate

$^{1}H$ and $^{13}C$ NMR data of the compound matches the reported literature.[S24]

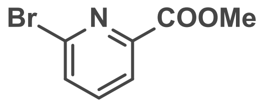


Methyl 6-bromopicolinate

$^{1}H$ and $^{13}C$ NMR data of the compound matches the reported literature.[S26]

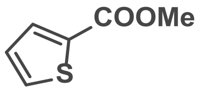


Methyl thiophene-2-carboxylate

$^{1}H$ and $^{13}C$ NMR data of the compound matches the reported literature.[S18]

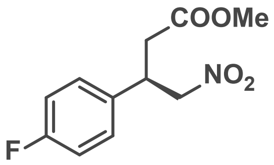


Methyl (*R*)-3-(4-fluorophenyl)-4-nitrobutanoate

5.46 g (94%) was isolated after extractive work-up.

$^{1}H$ and $^{13}C$ NMR data of the compound matches the reported literature.[S27] $[\alpha]_D^{25}$= +17.4 (c= 0.5, $CH_2Cl_2$).

## 5. Collection of NMR spectra

300 MHz, $CDCl_3$

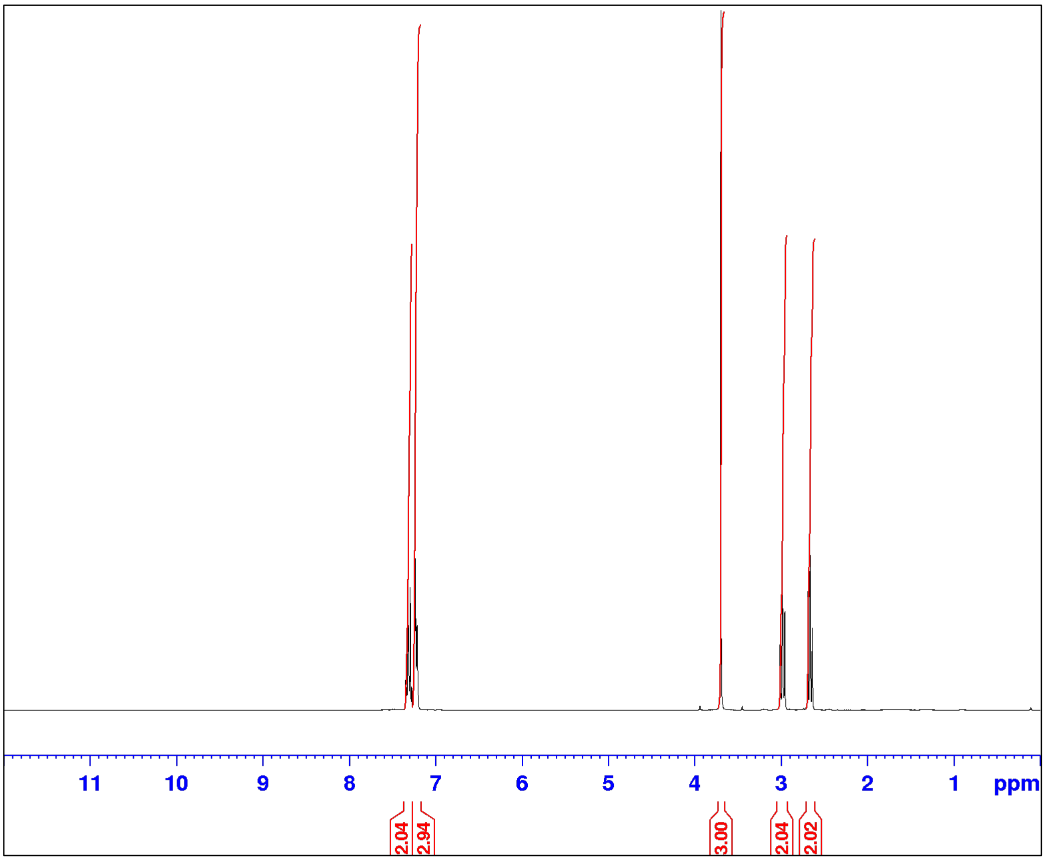


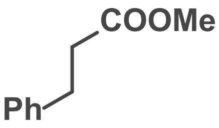


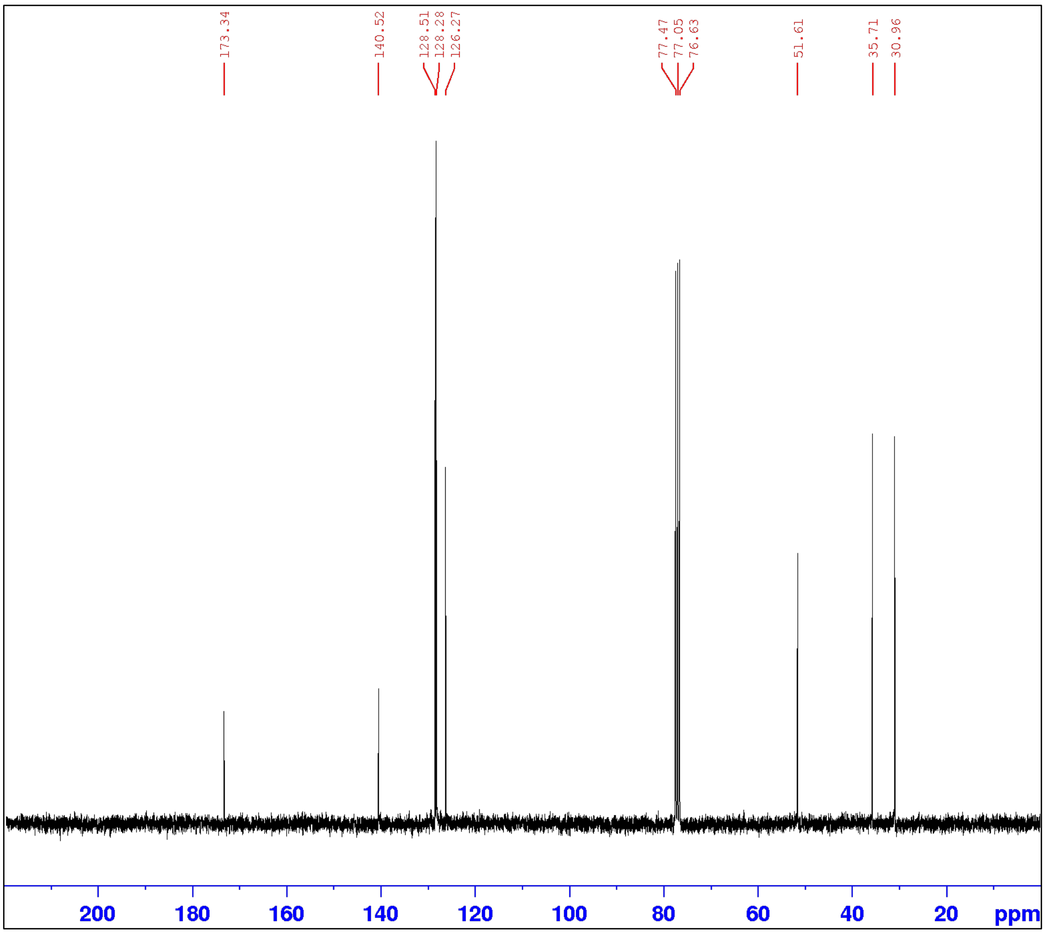


75 MHz, $CDCl_3$

300 MHz, $CDCl_3$

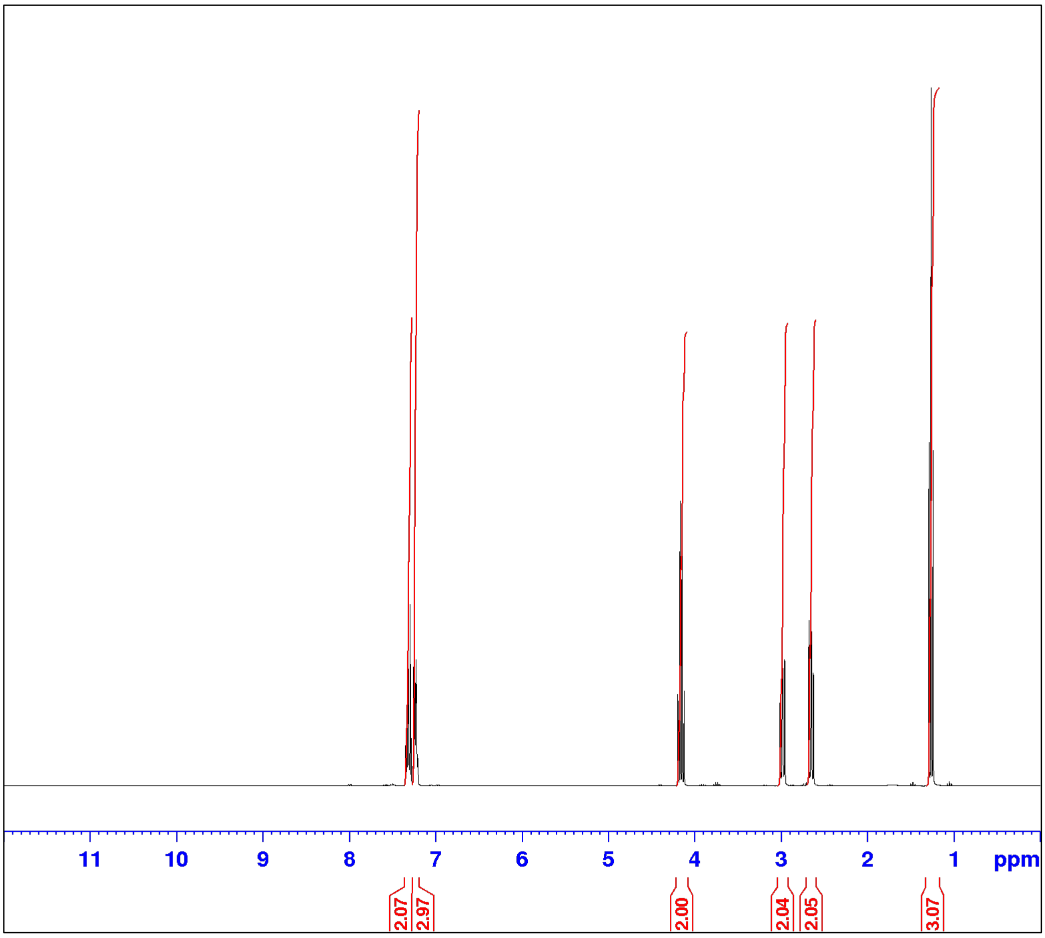


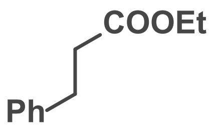


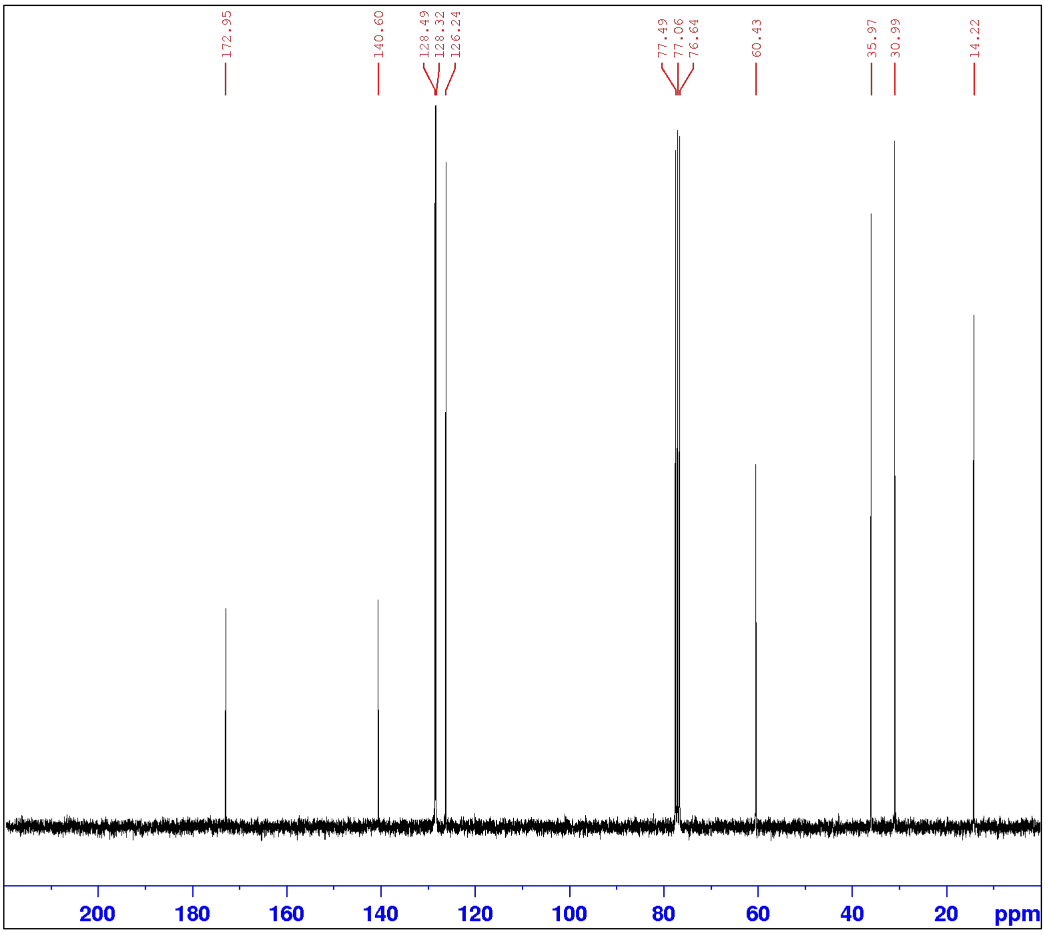


75 MHz, $CDCl_3$

300 MHz, $CDCl_3$

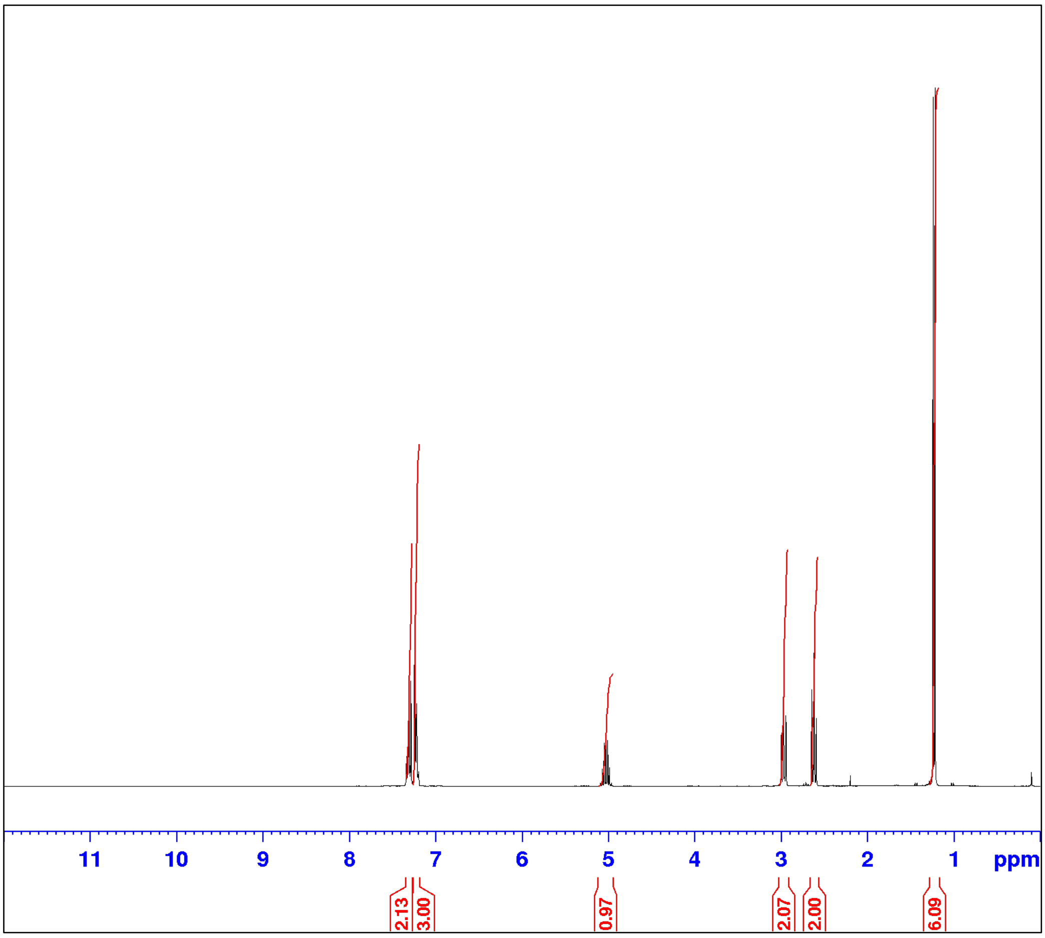


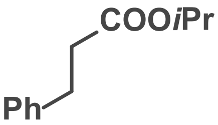


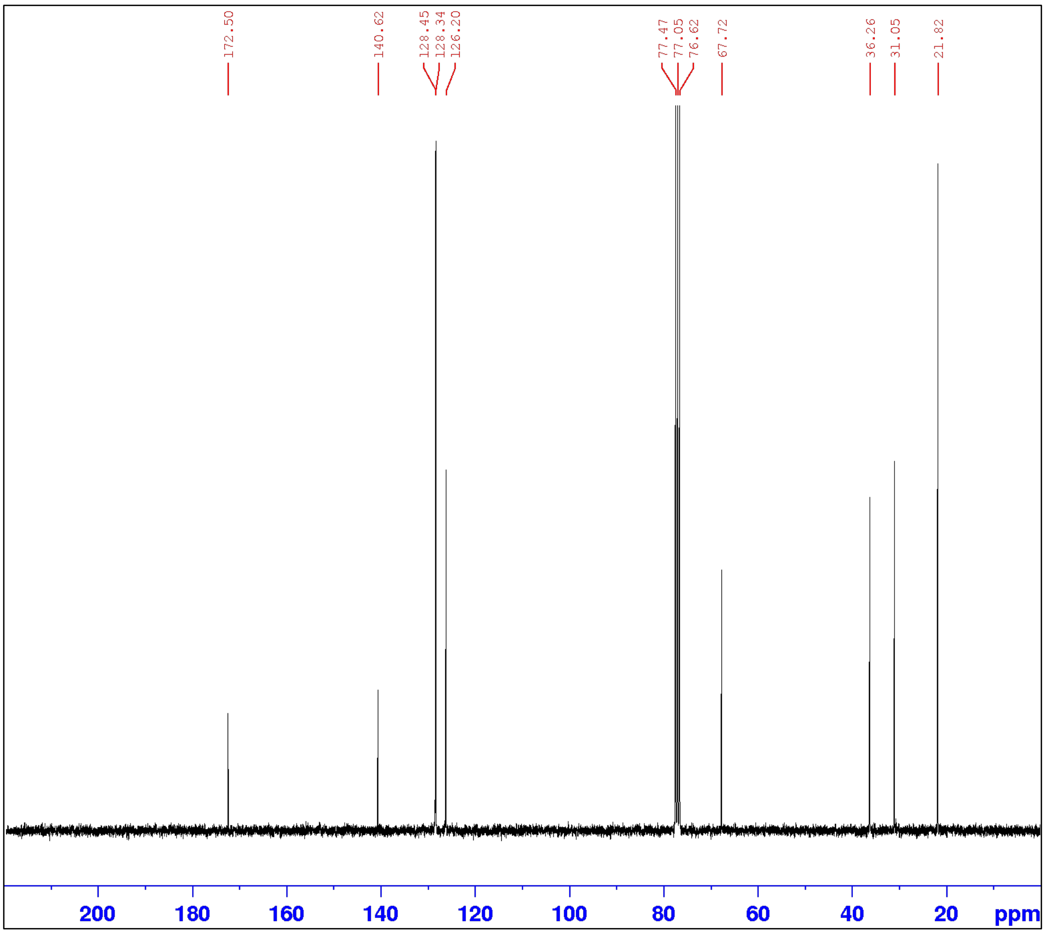


75 MHz, $CDCl_3$

300 MHz, $CDCl_3$

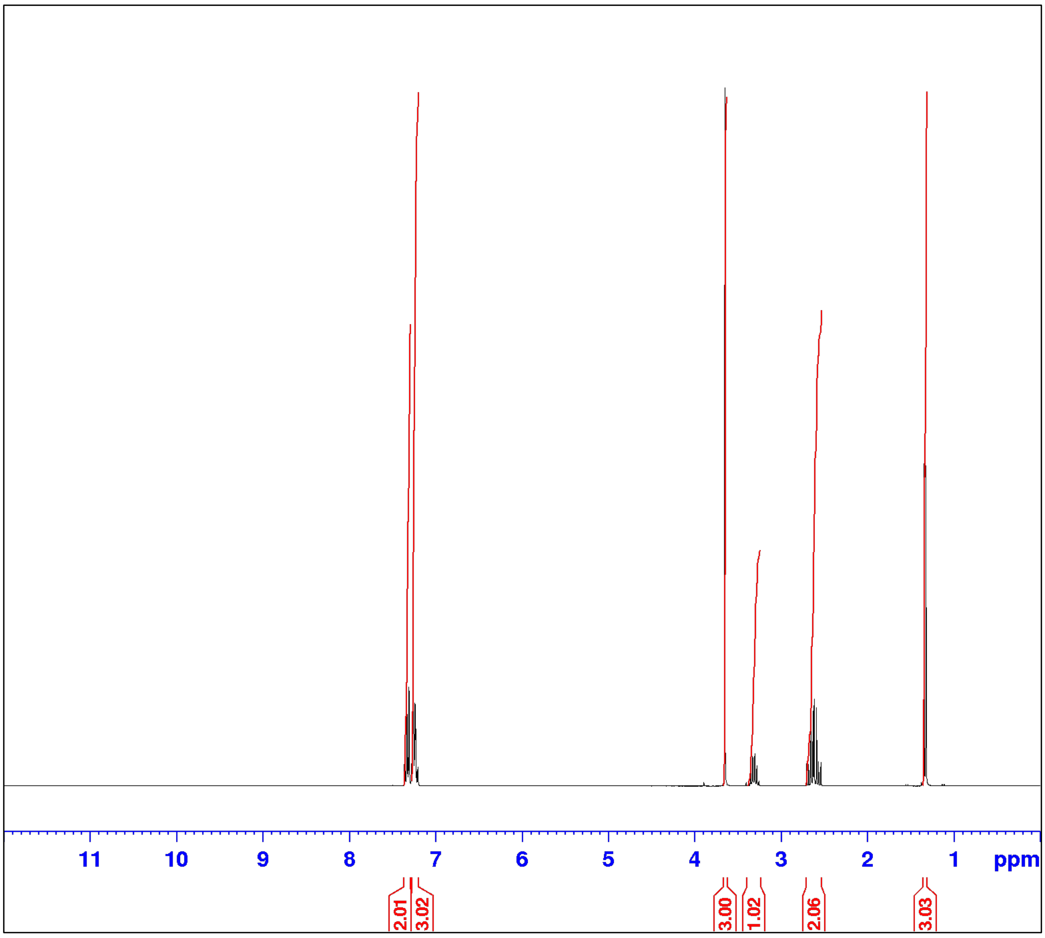


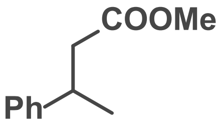


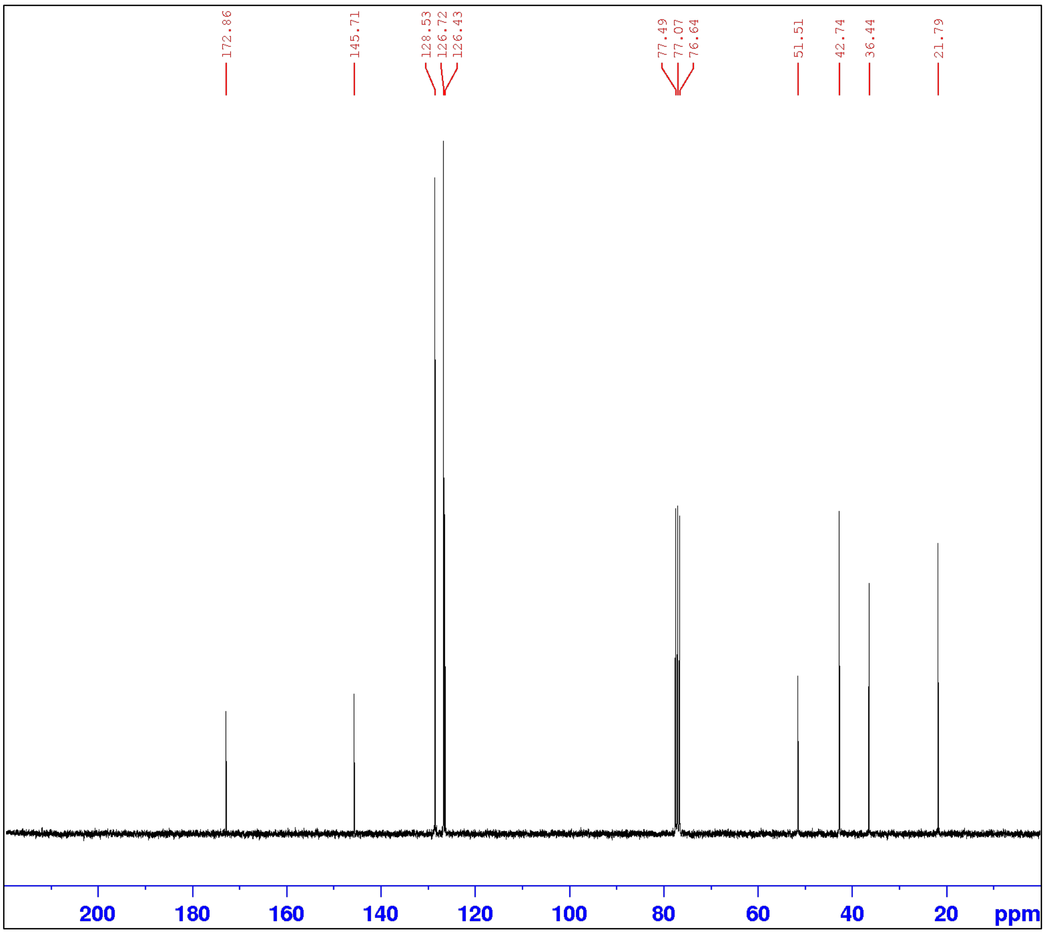


75 MHz, $CDCl_3$

300 MHz, $CDCl_3$

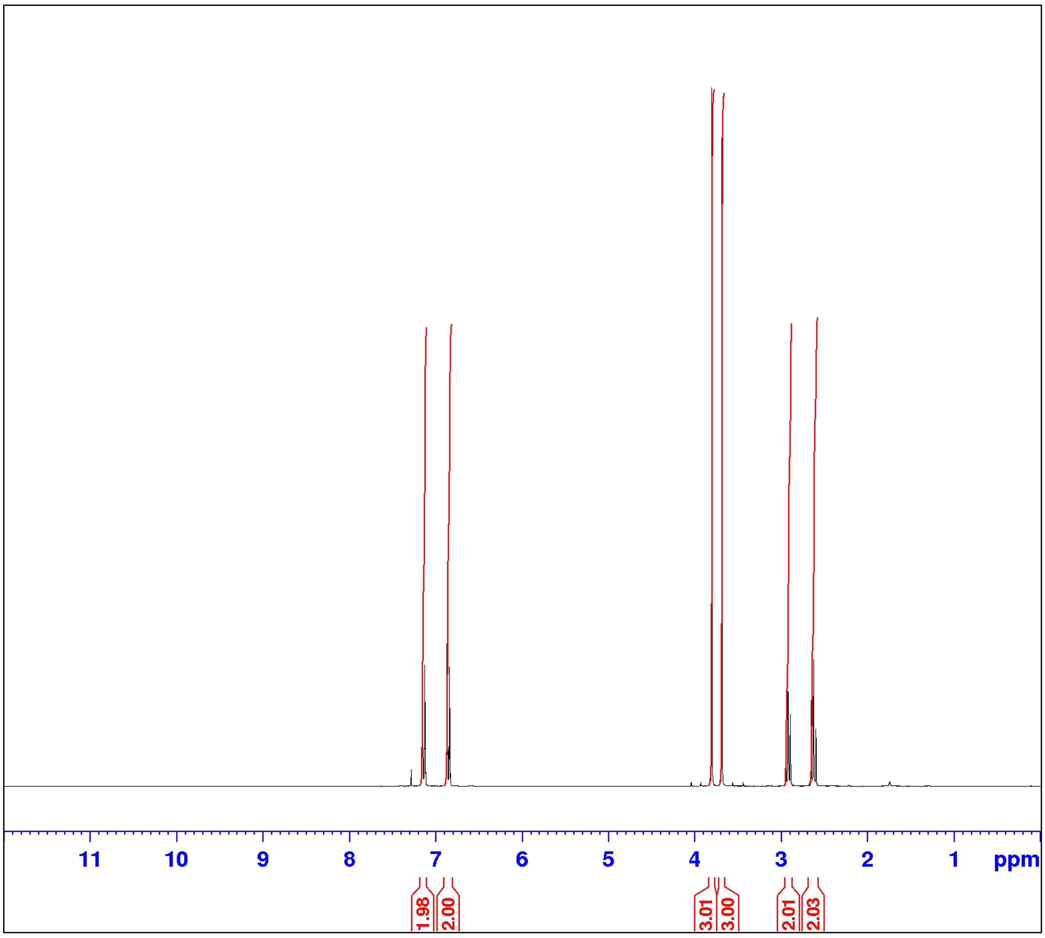


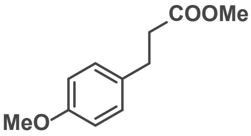


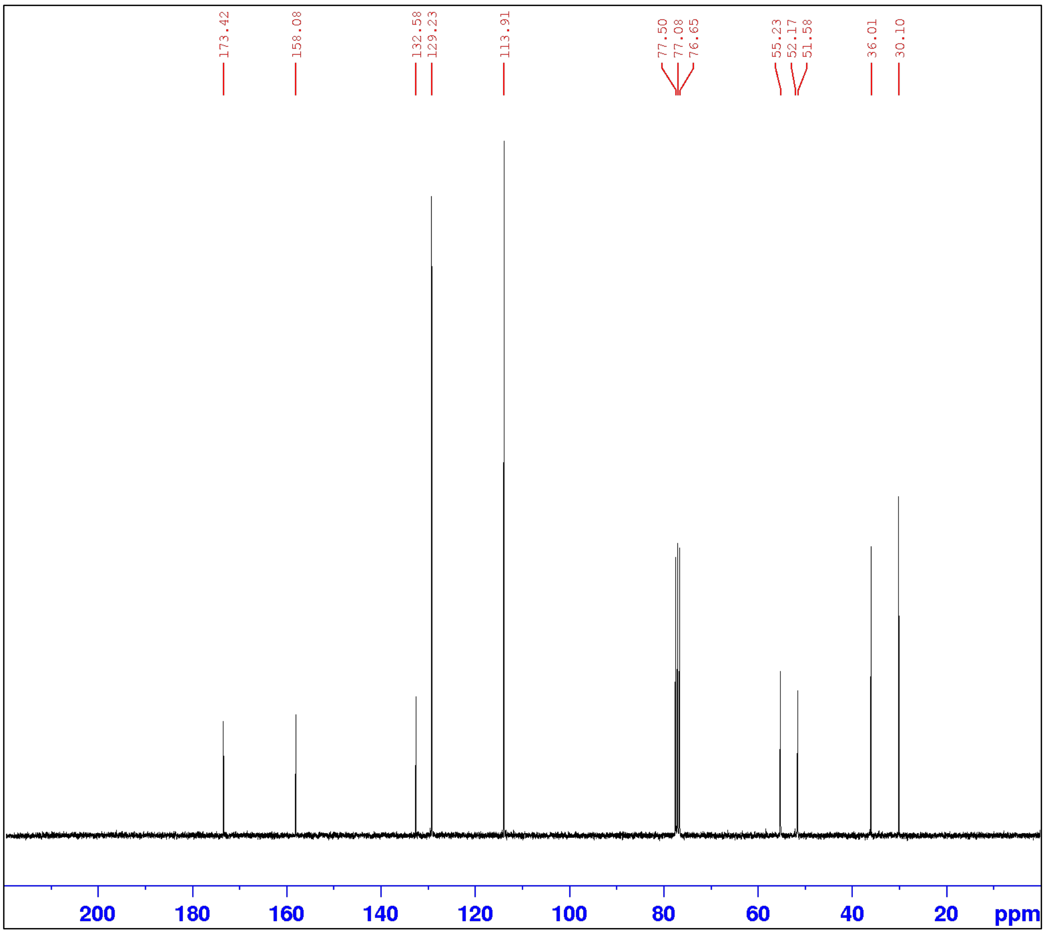


75 MHz, $CDCl_3$

300 MHz, $CDCl_3$

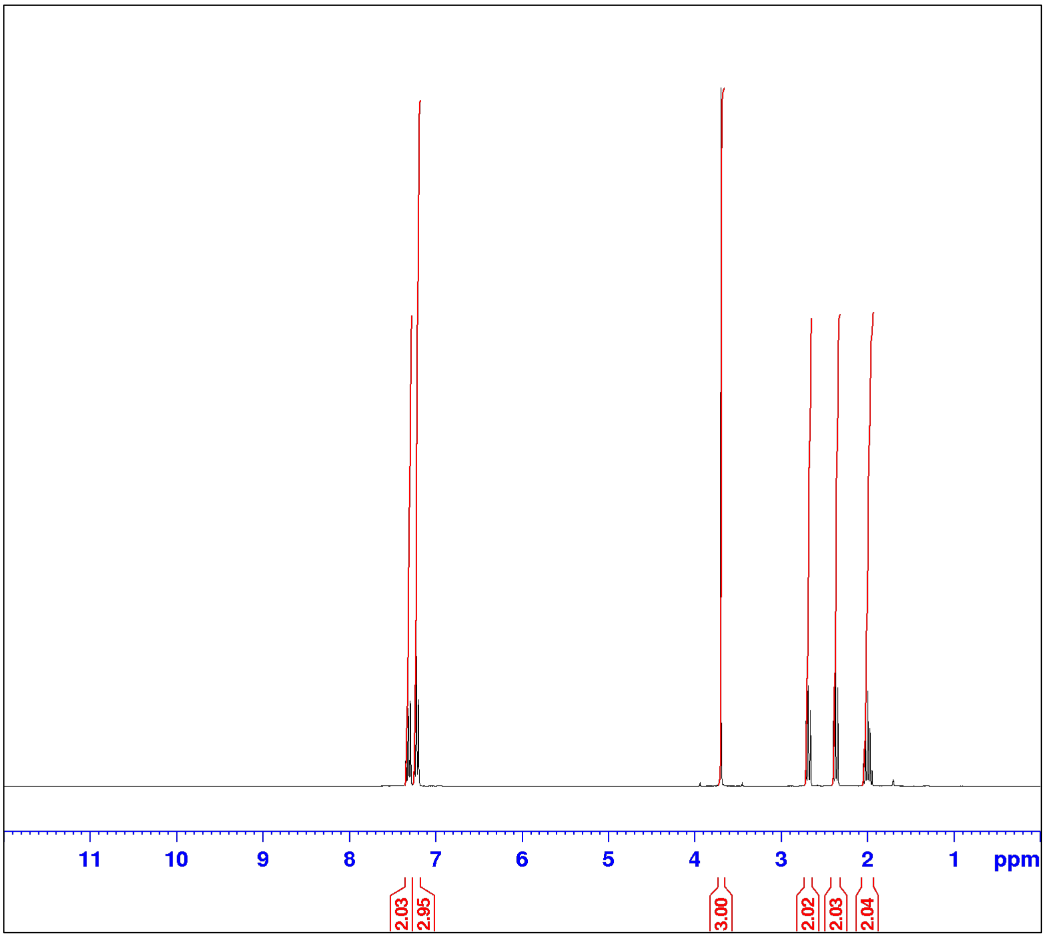


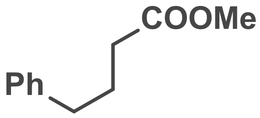


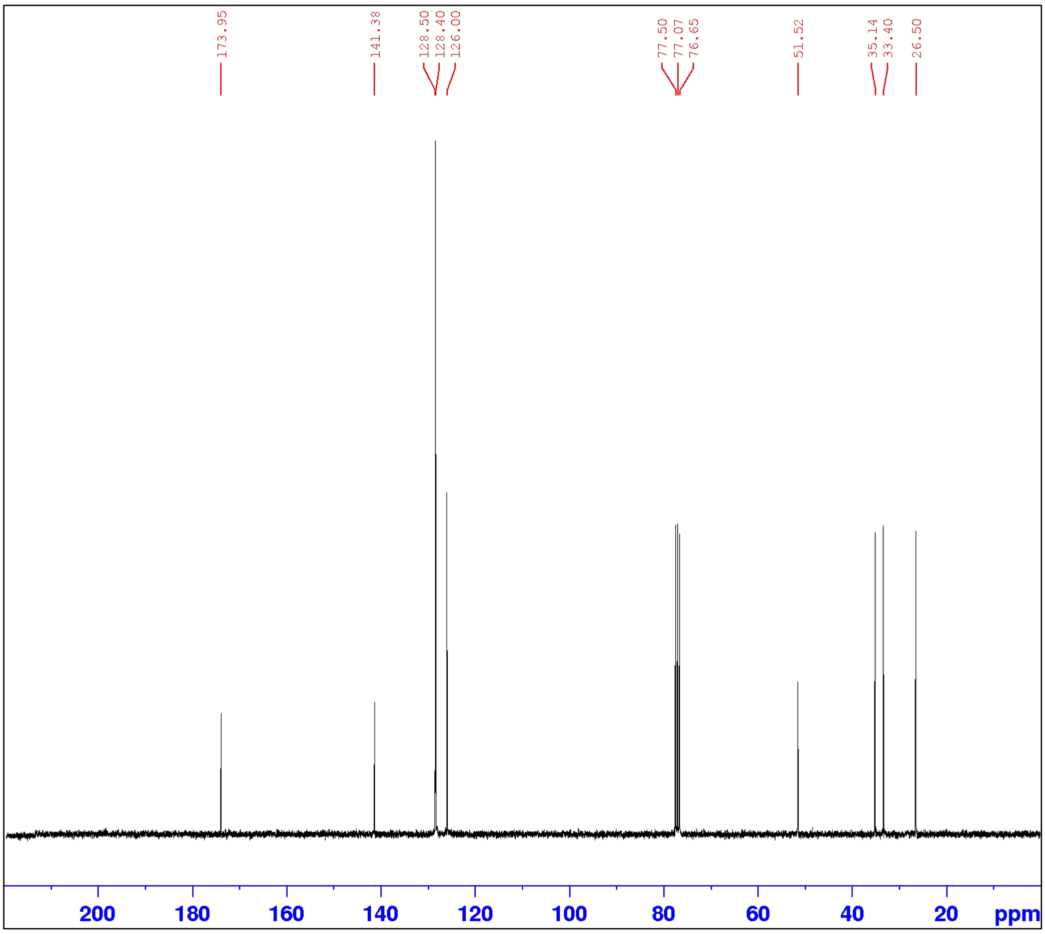


75 MHz, $CDCl_3$

300 MHz, $CDCl_3$

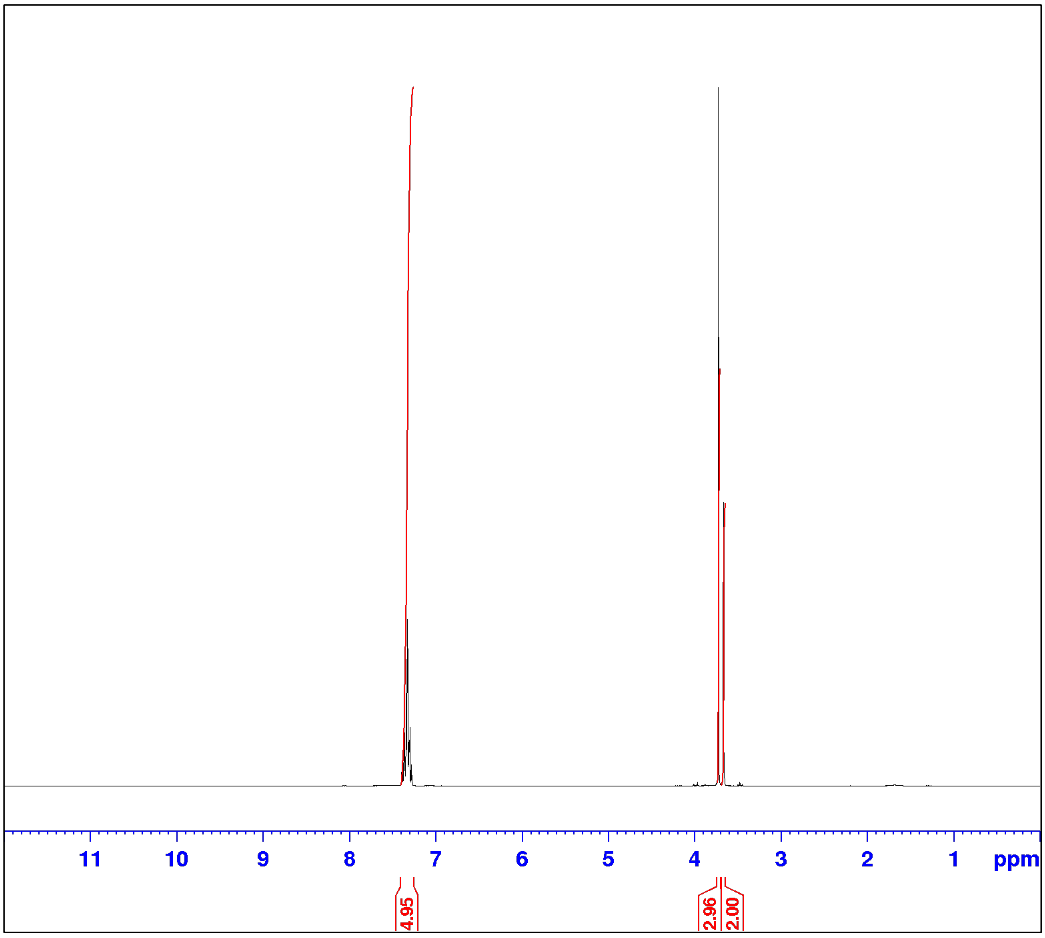


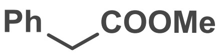


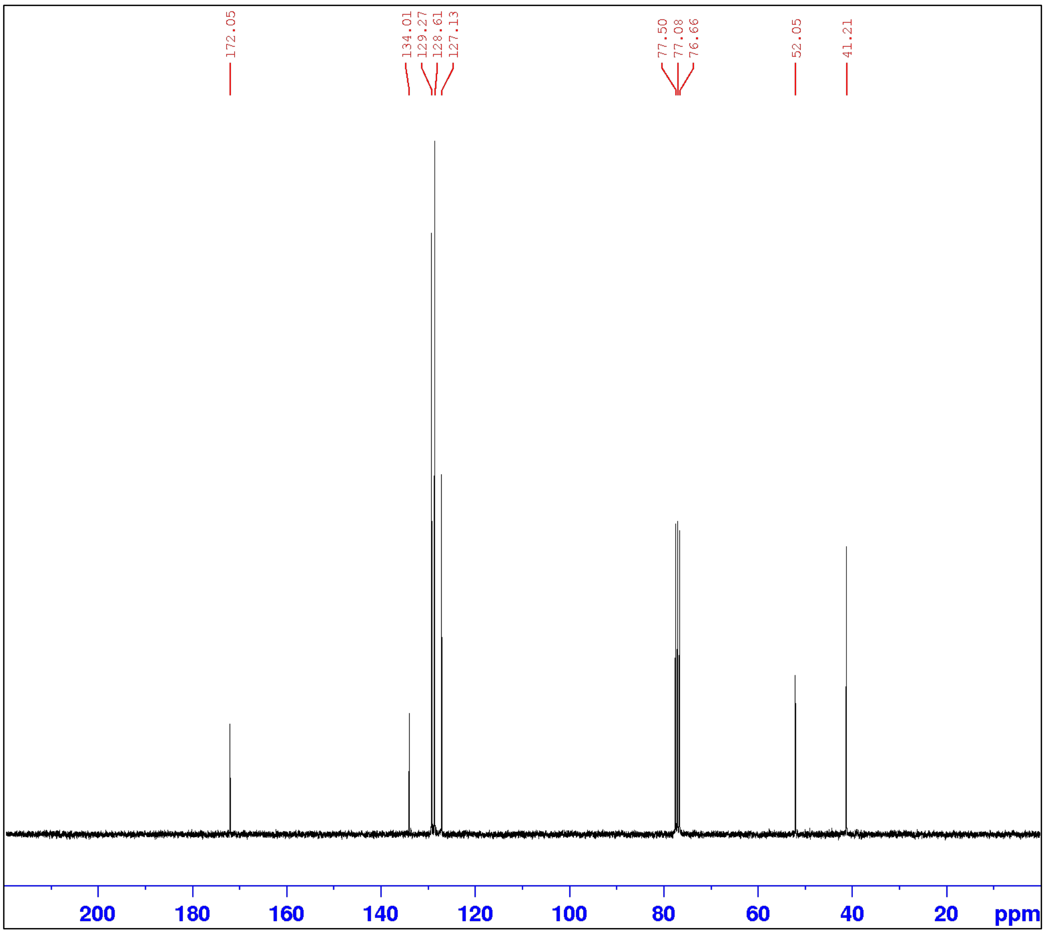


75 MHz, $CDCl_3$

300 MHz, $CDCl_3$

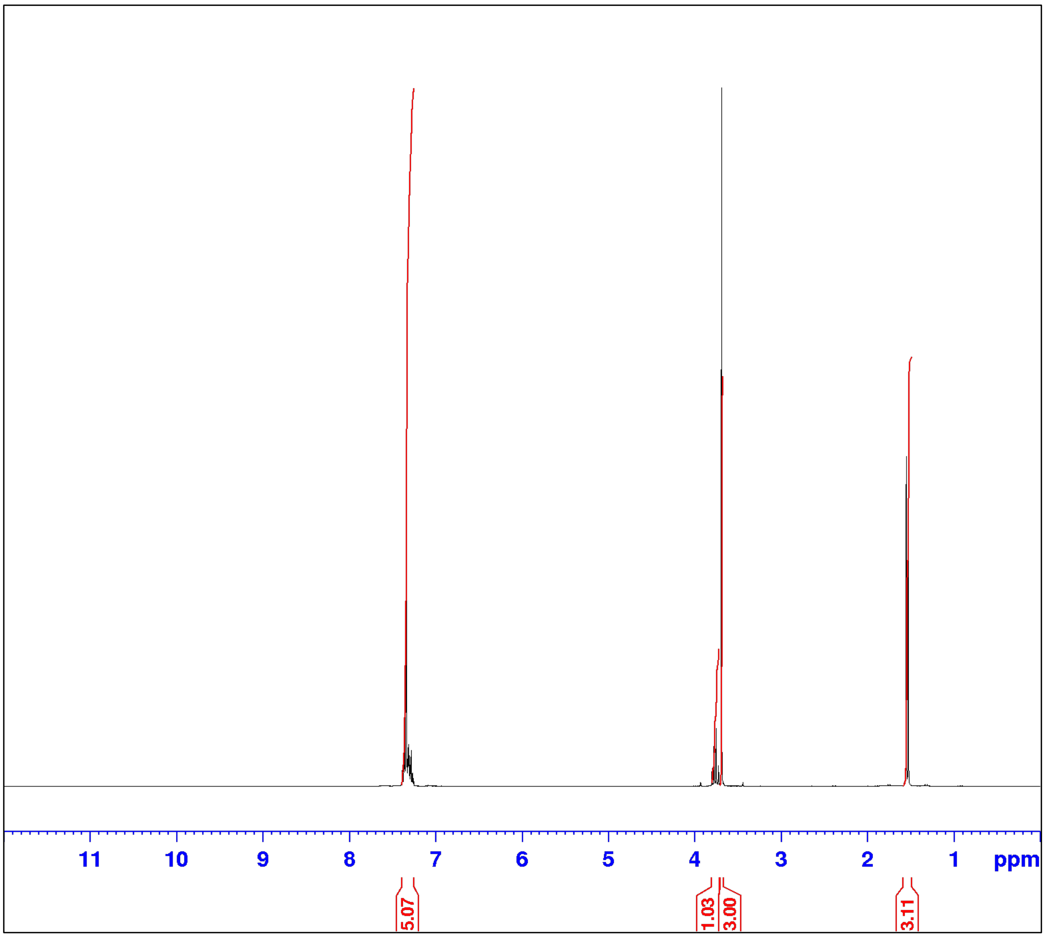


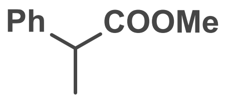


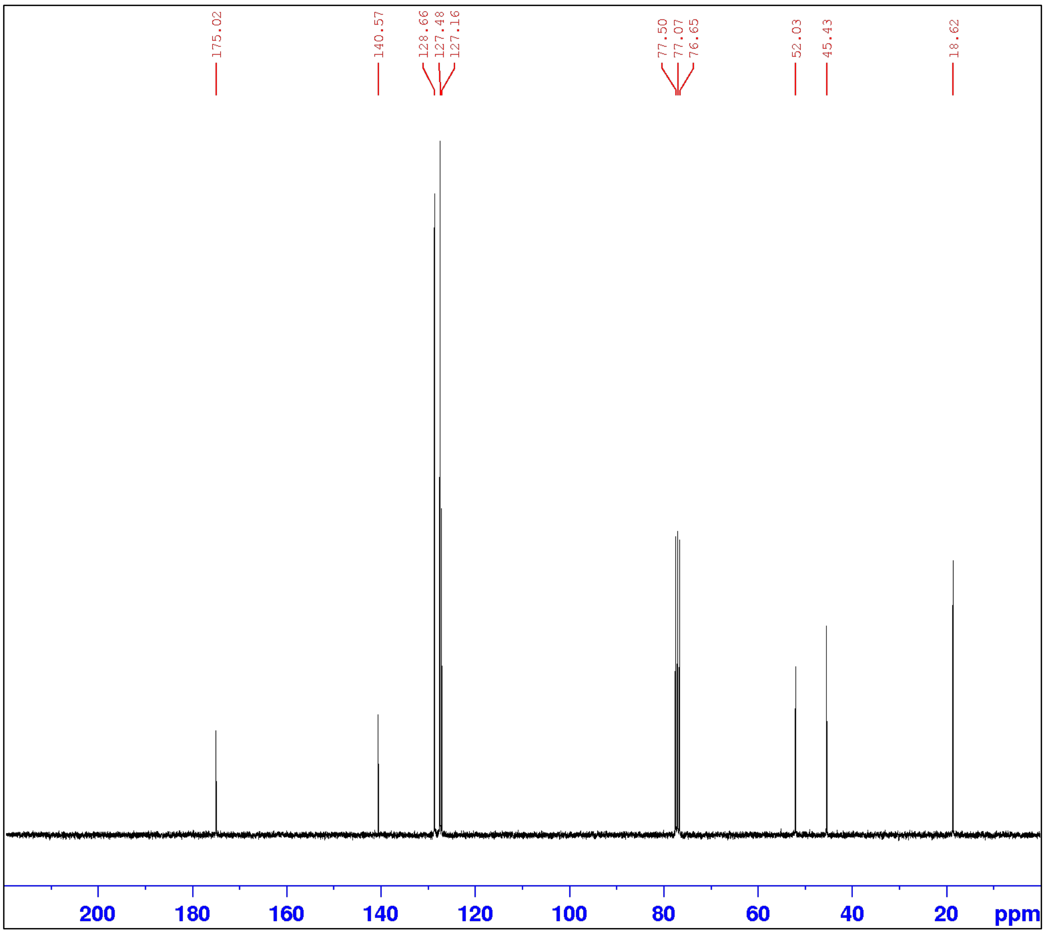


75 MHz, $CDCl_3$

300 MHz, $CDCl_3$

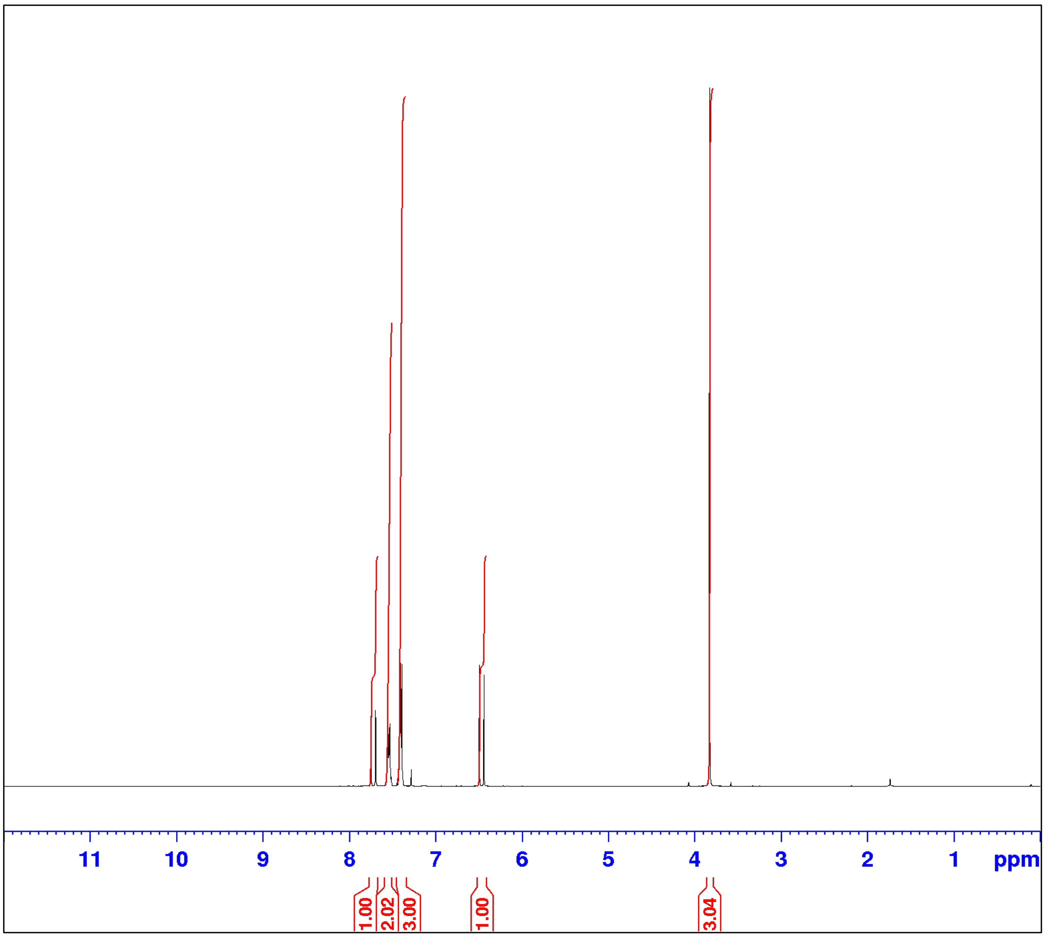


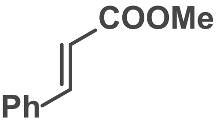


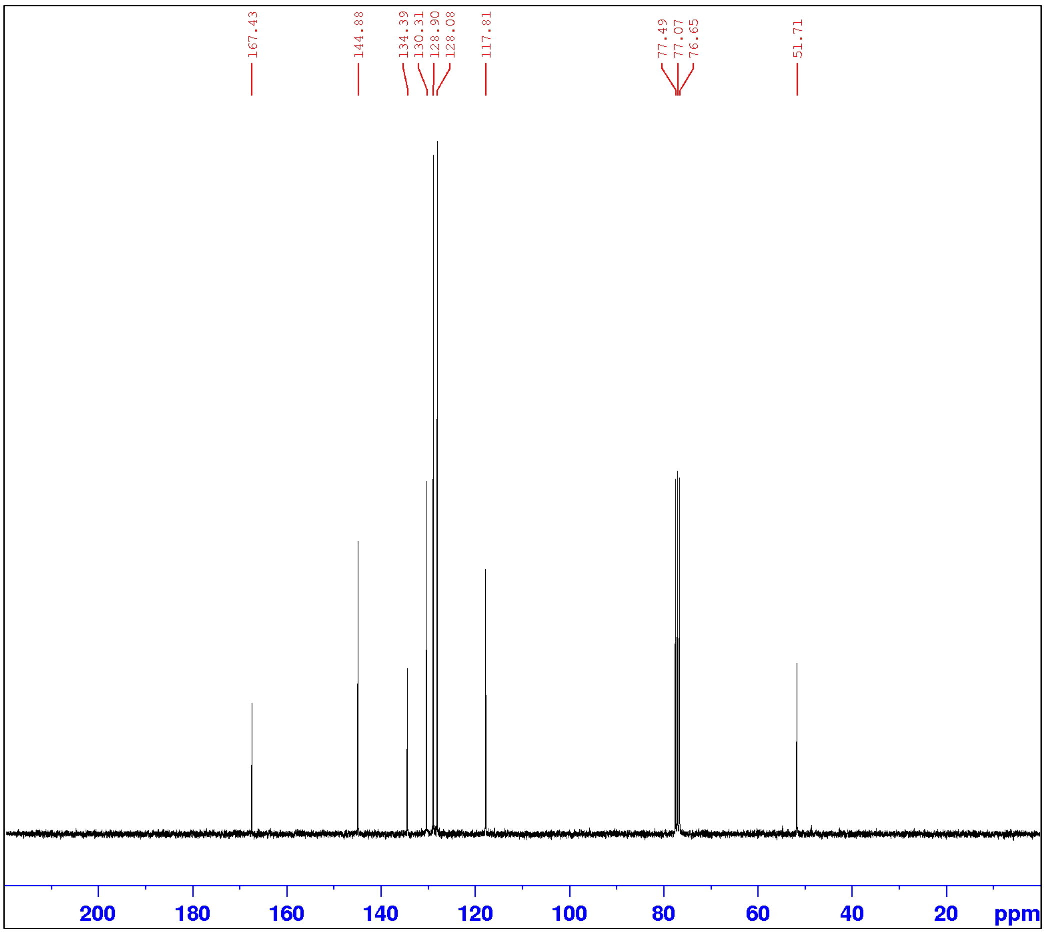


75 MHz, $CDCl_3$

300 MHz, $CDCl_3$

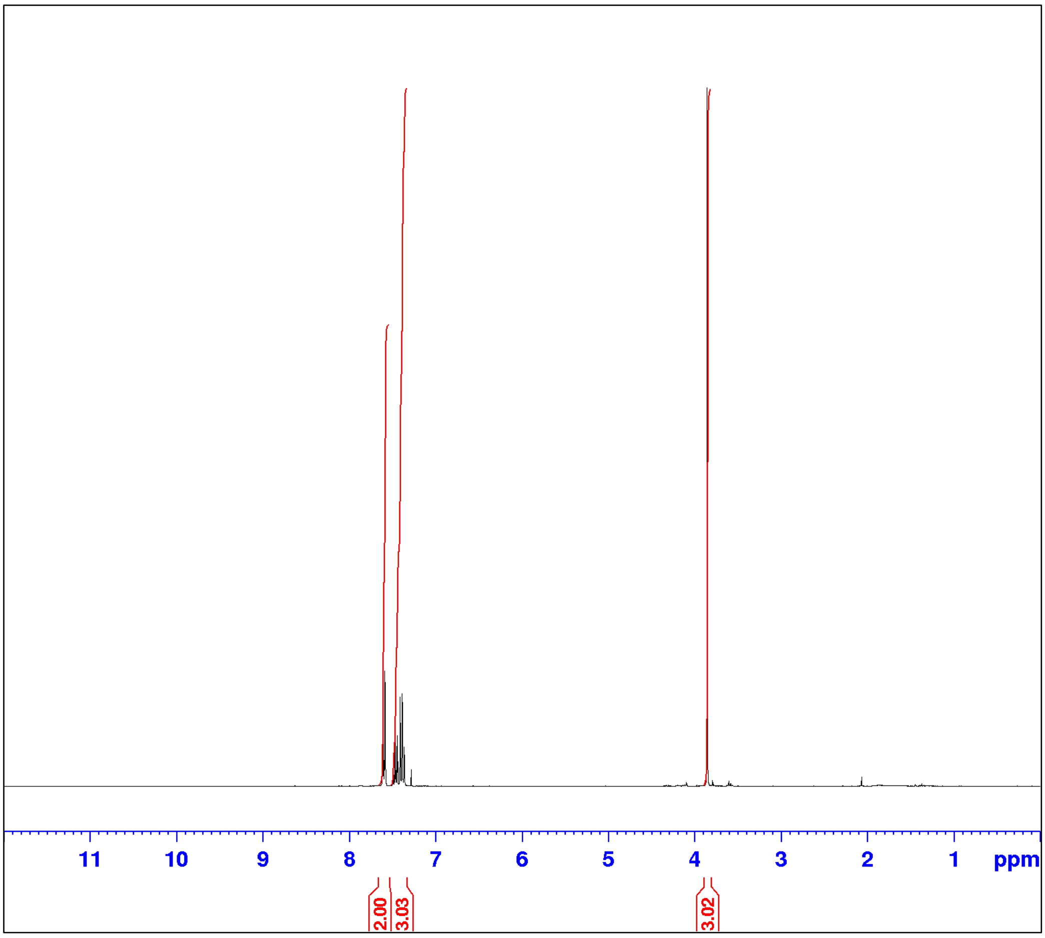


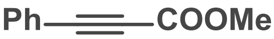


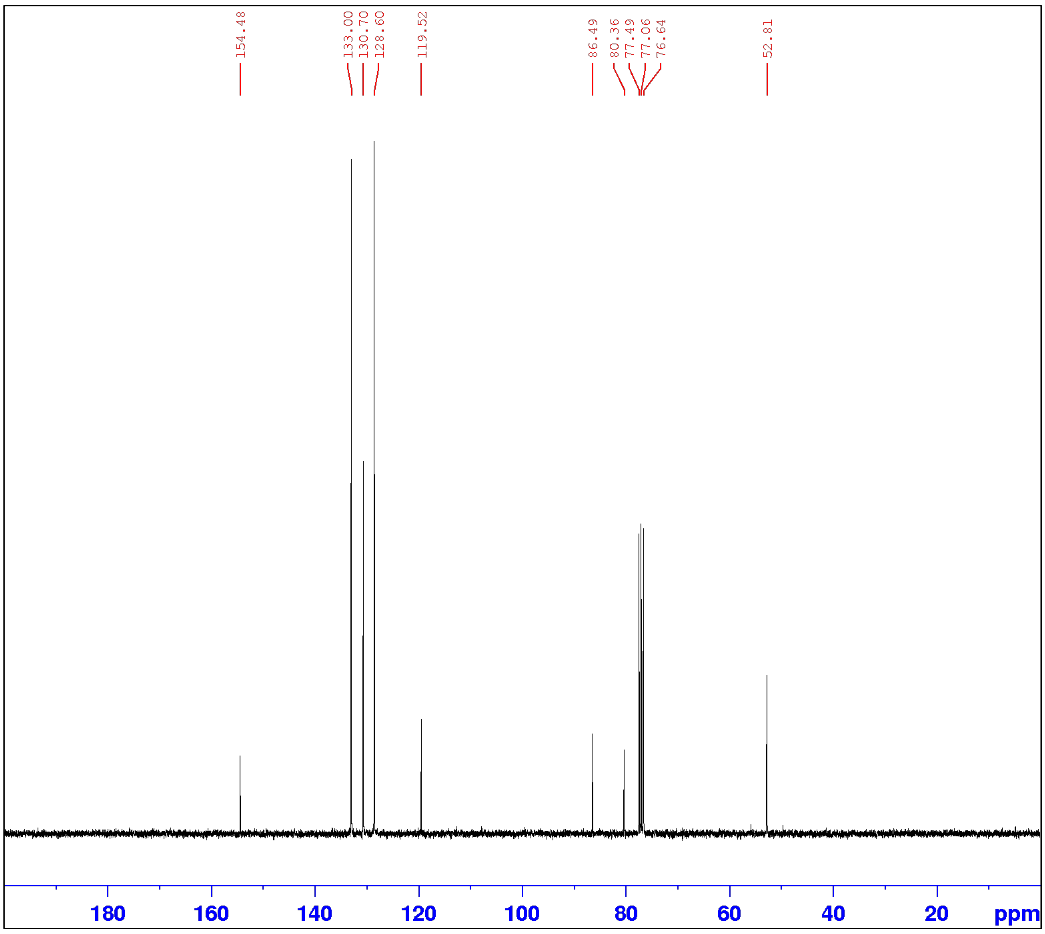


75 MHz, $CDCl_3$

300 MHz, $CDCl_3$

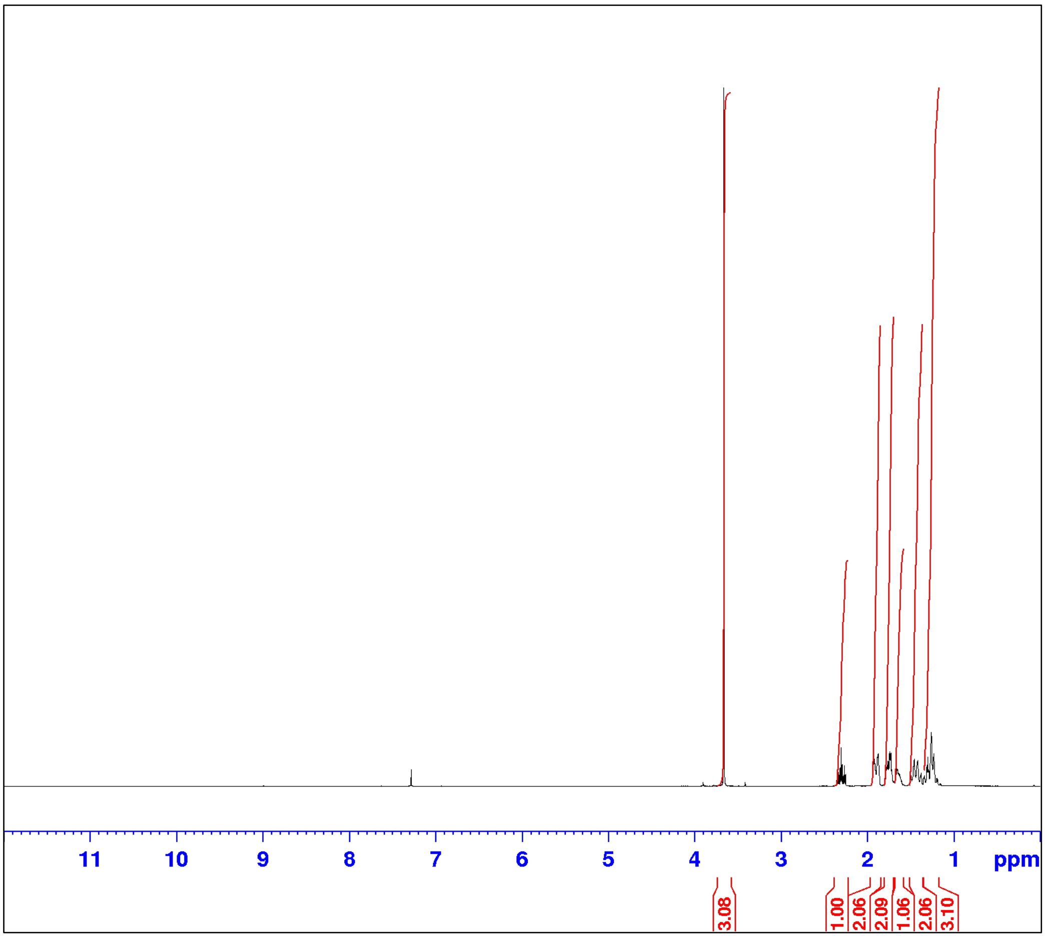


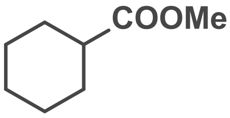


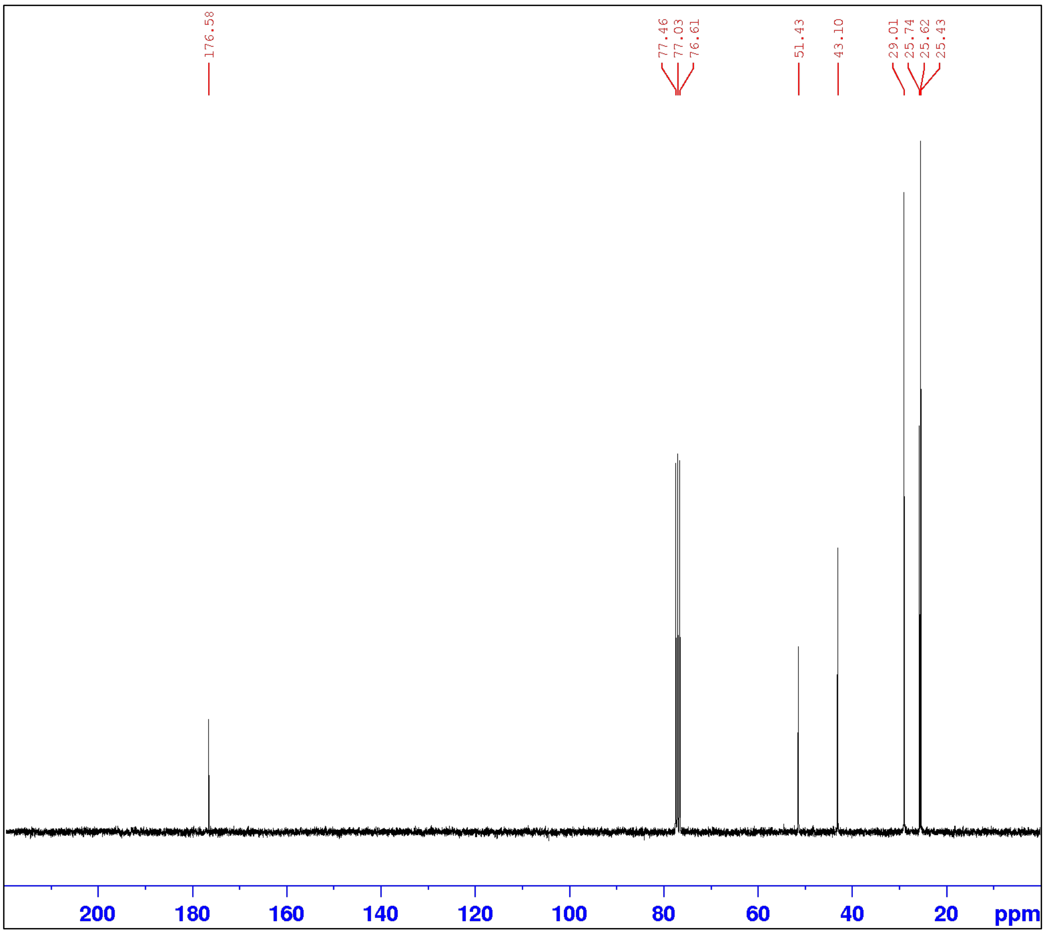


75 MHz, $CDCl_3$

300 MHz, $CDCl_3$

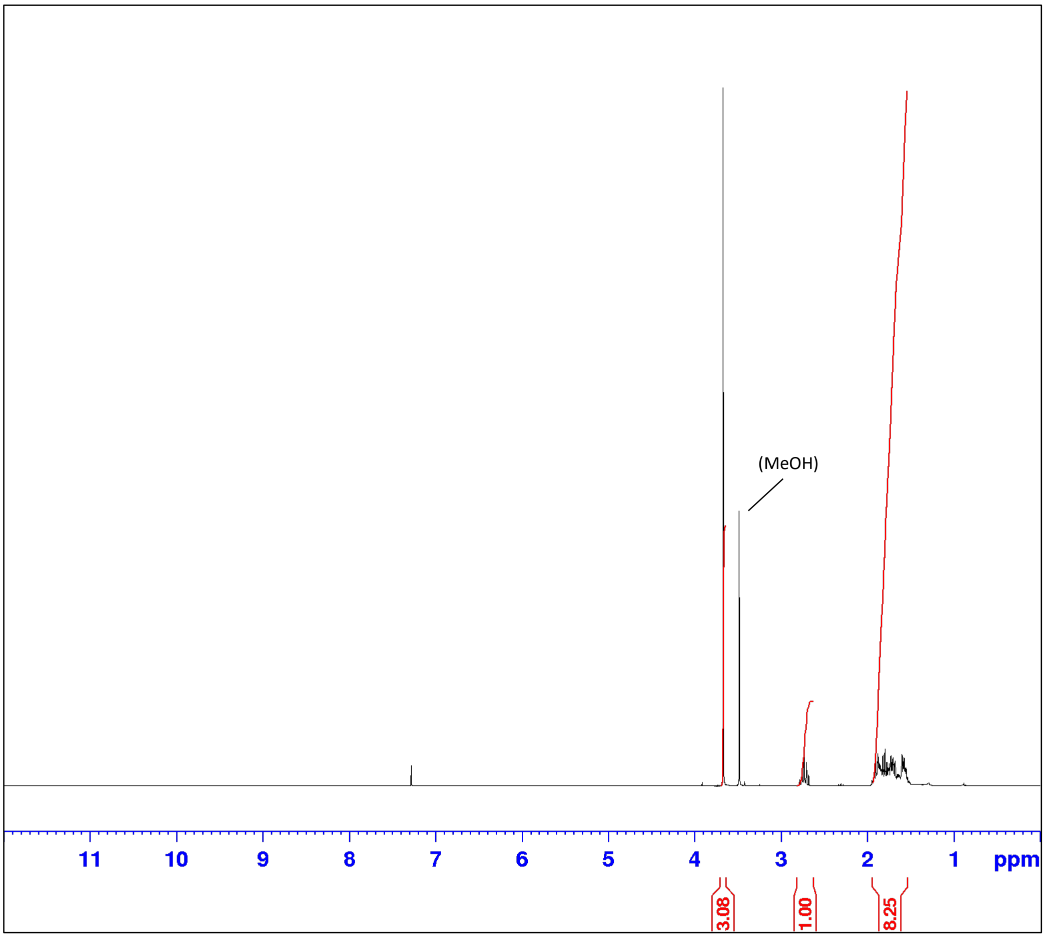


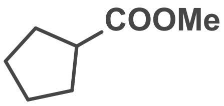


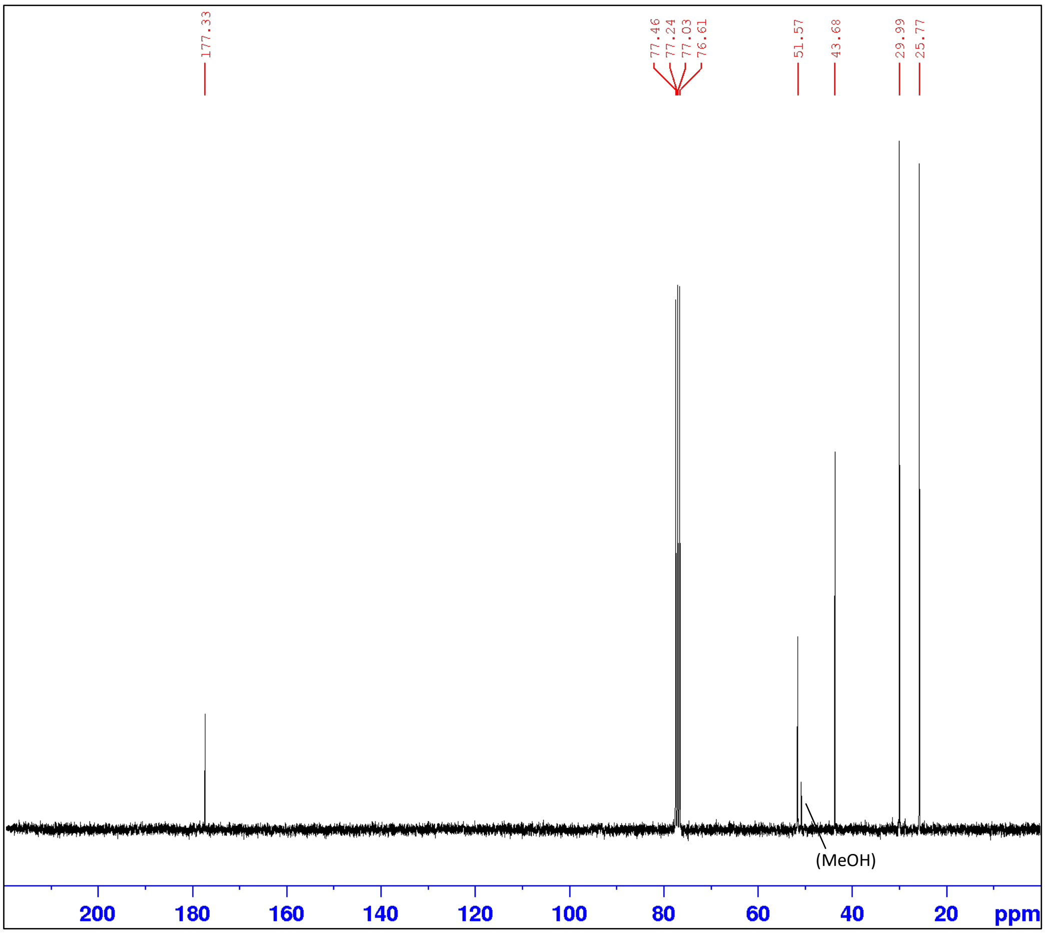


75 MHz, $CDCl_3$

300 MHz, $CDCl_3$

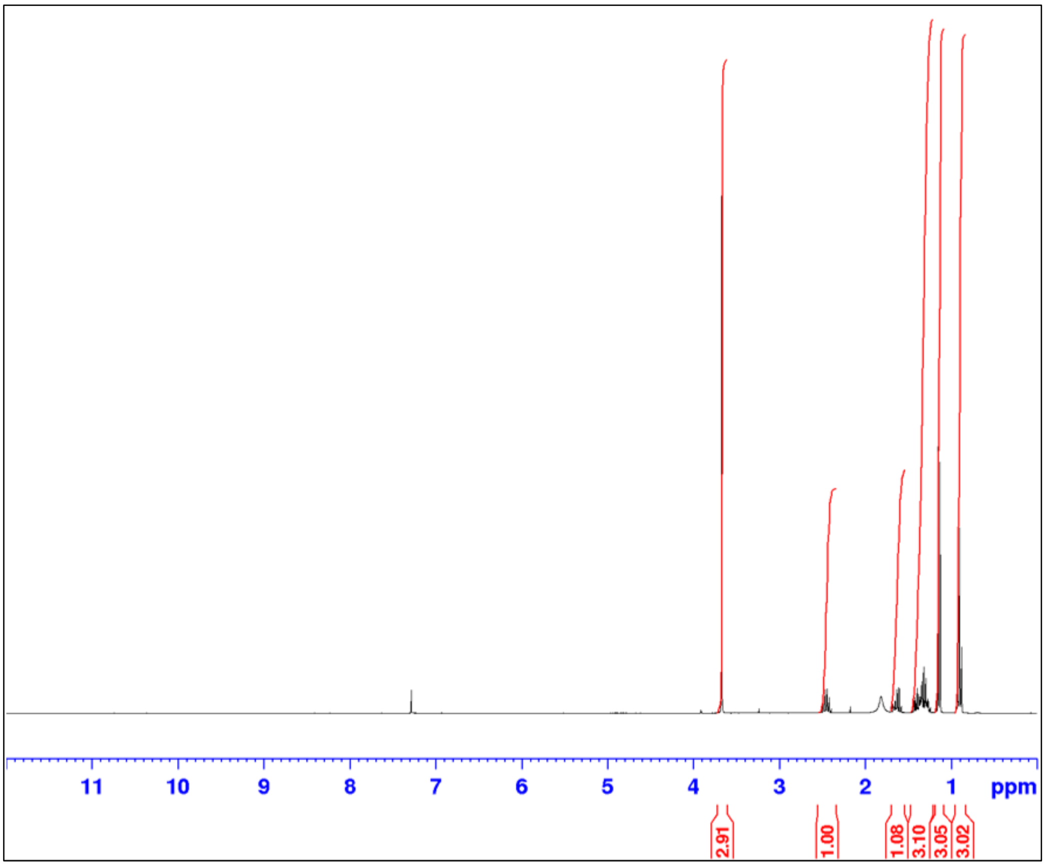


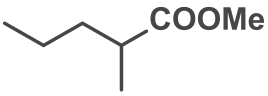


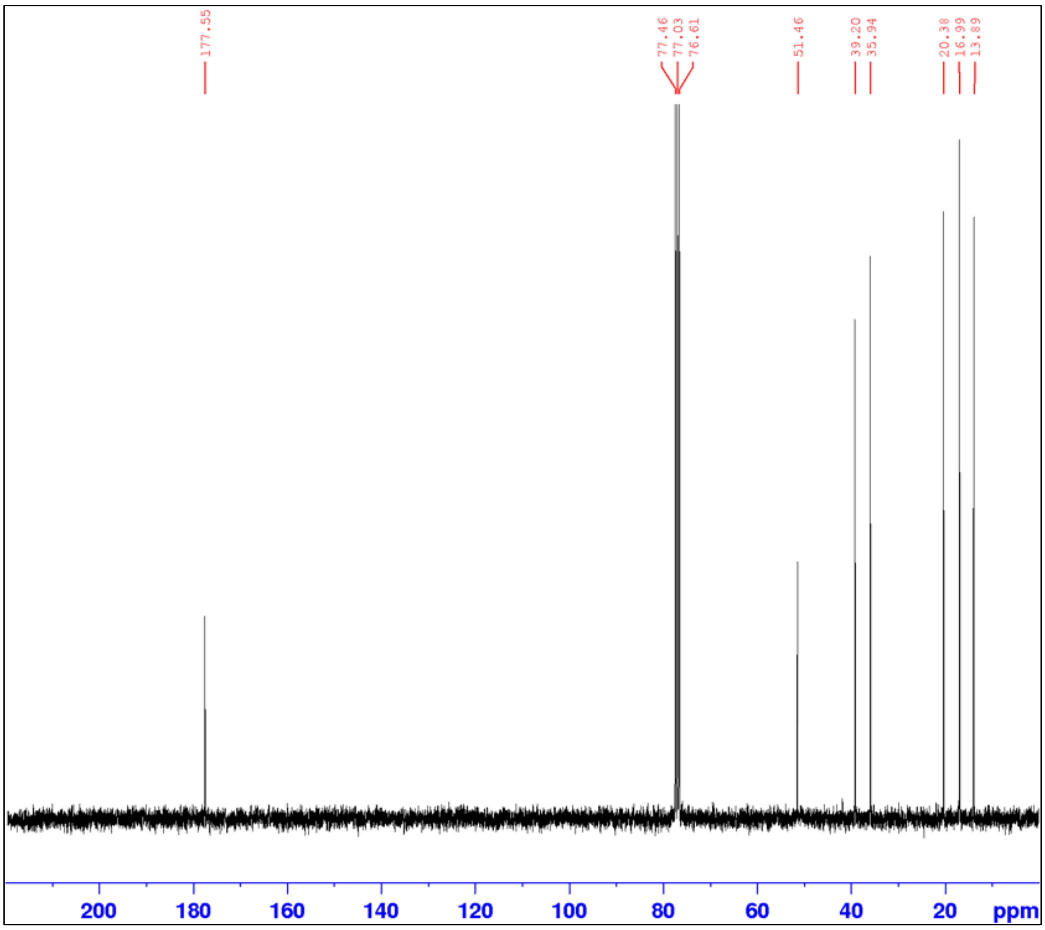


75 MHz, $CDCl_3$

300 MHz, $CDCl_3$

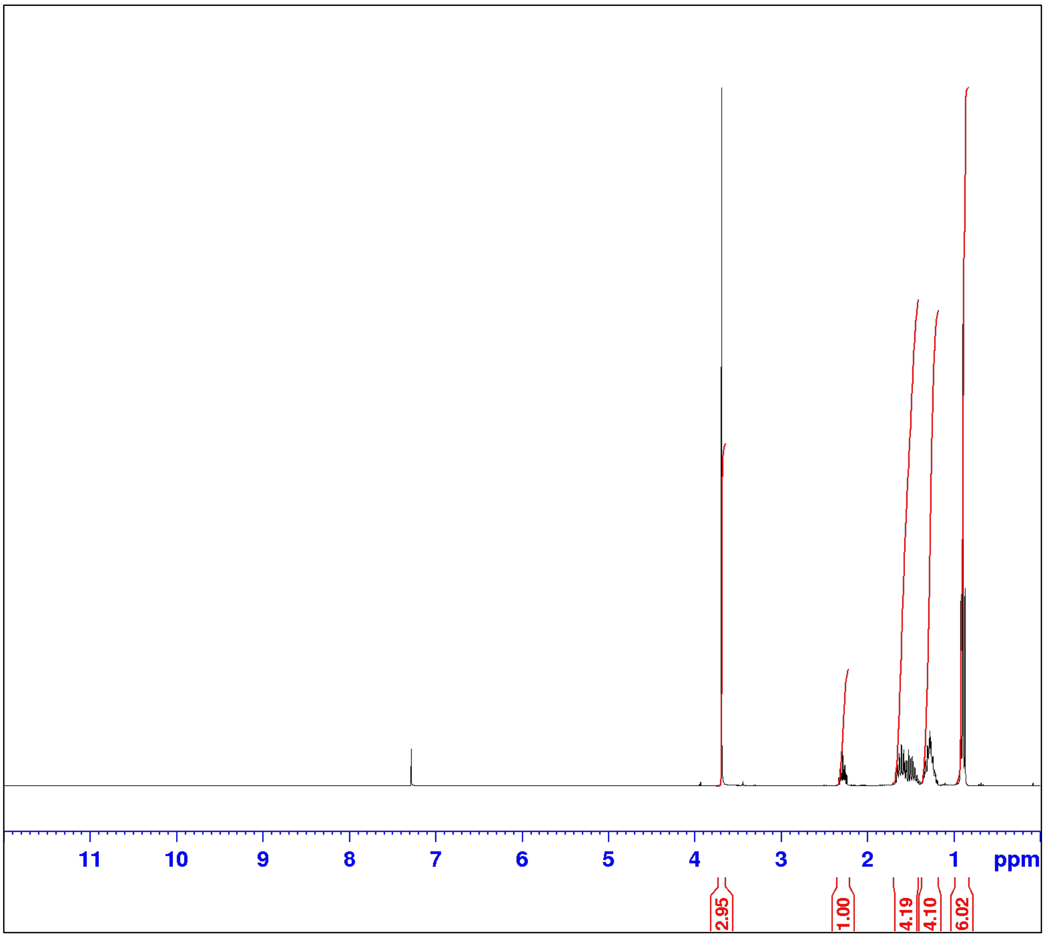


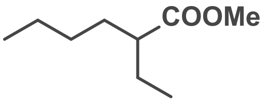


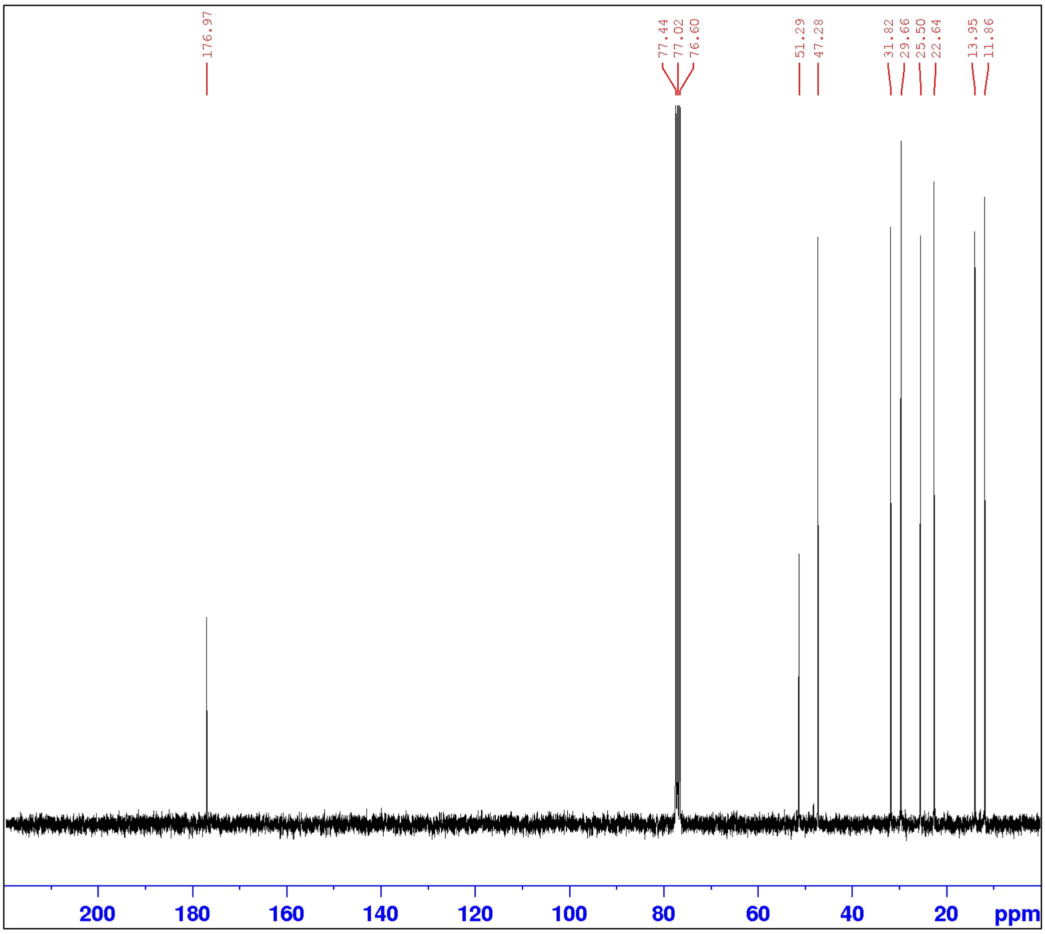


75 MHz, $CDCl_3$

300 MHz, $CDCl_3$

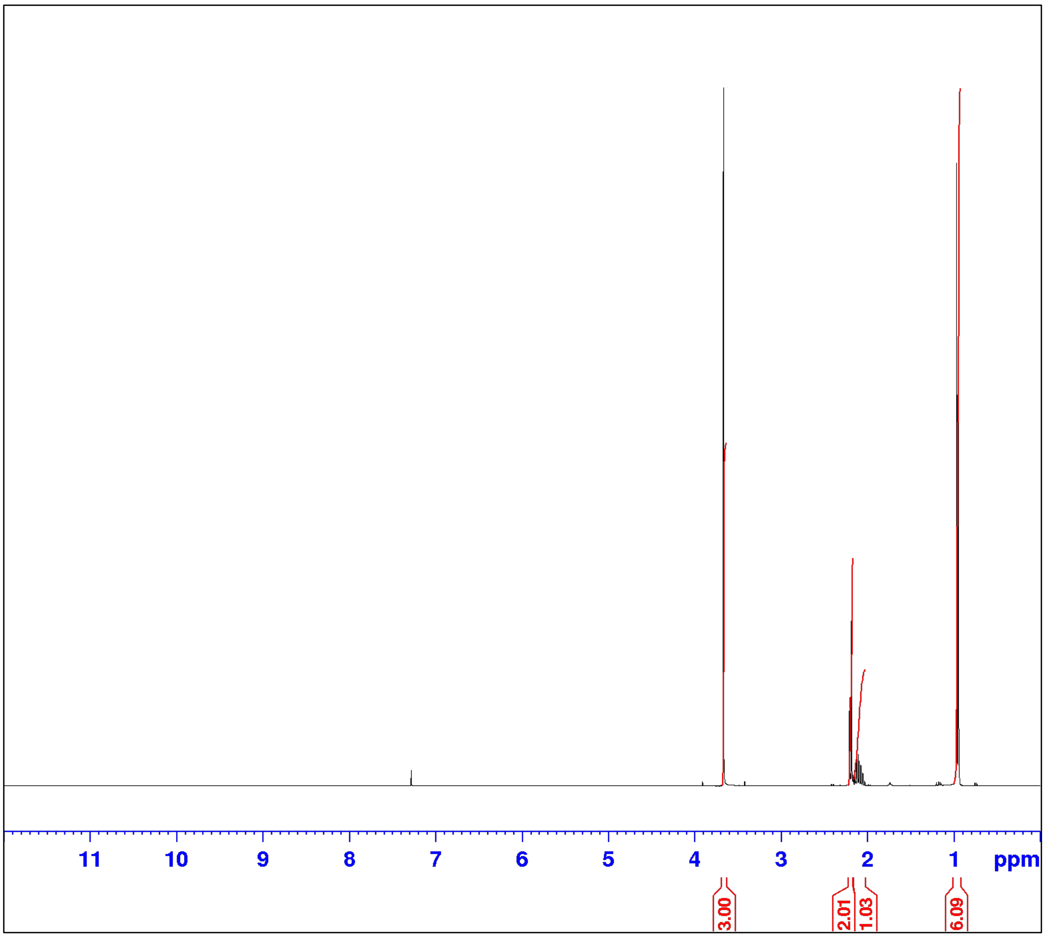


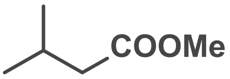


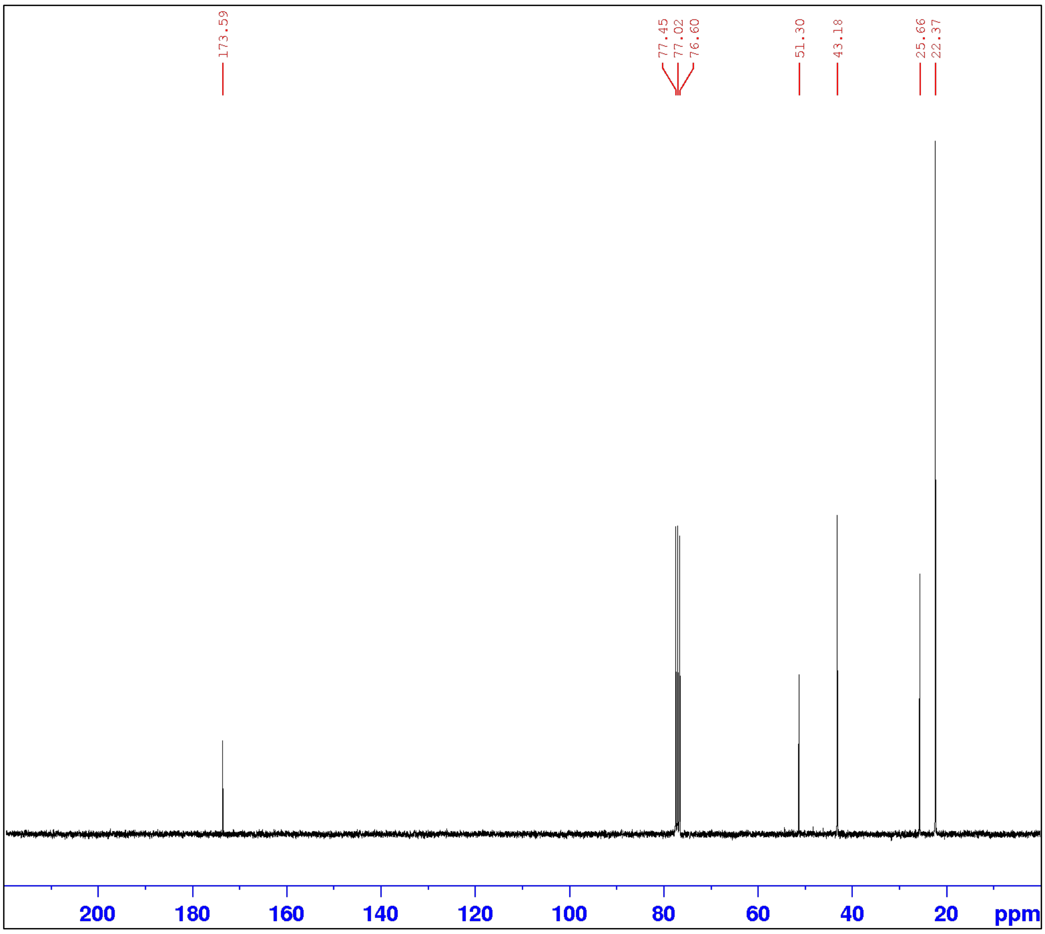


75 MHz, $CDCl_3$

300 MHz, $CDCl_3$

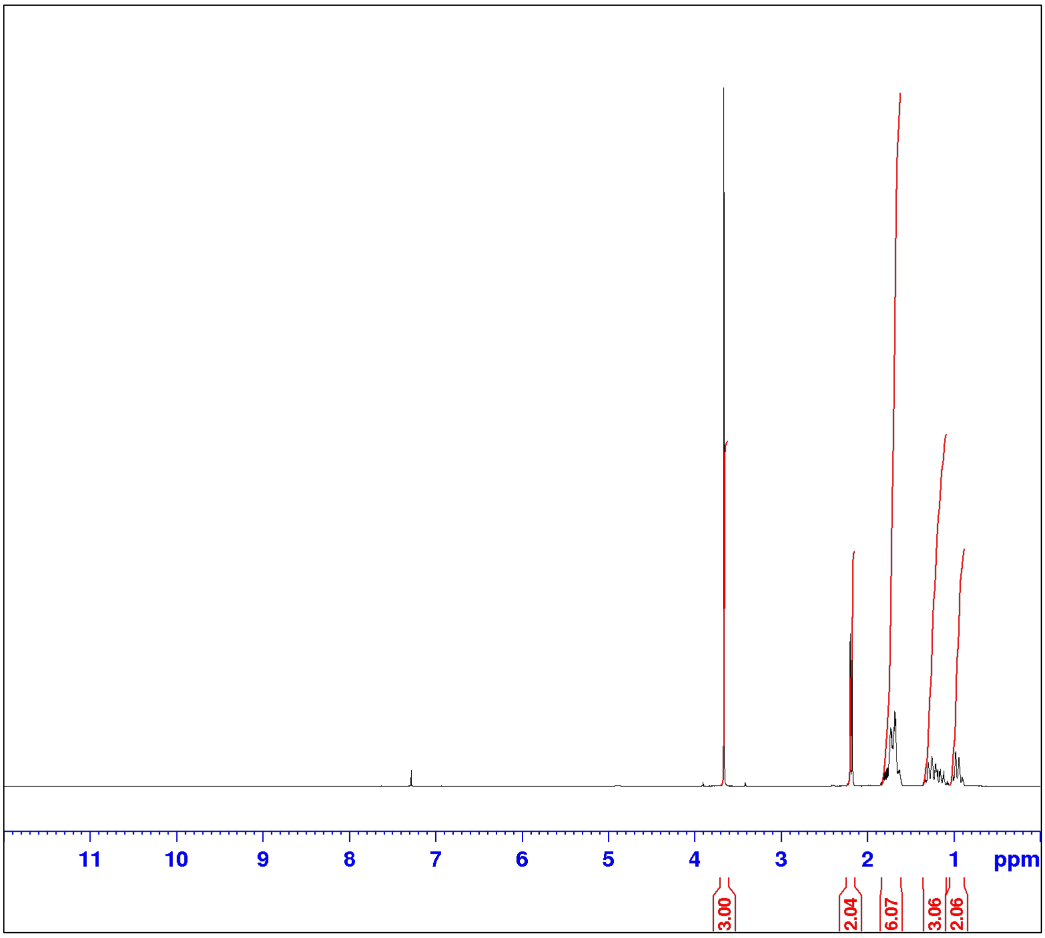


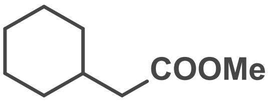


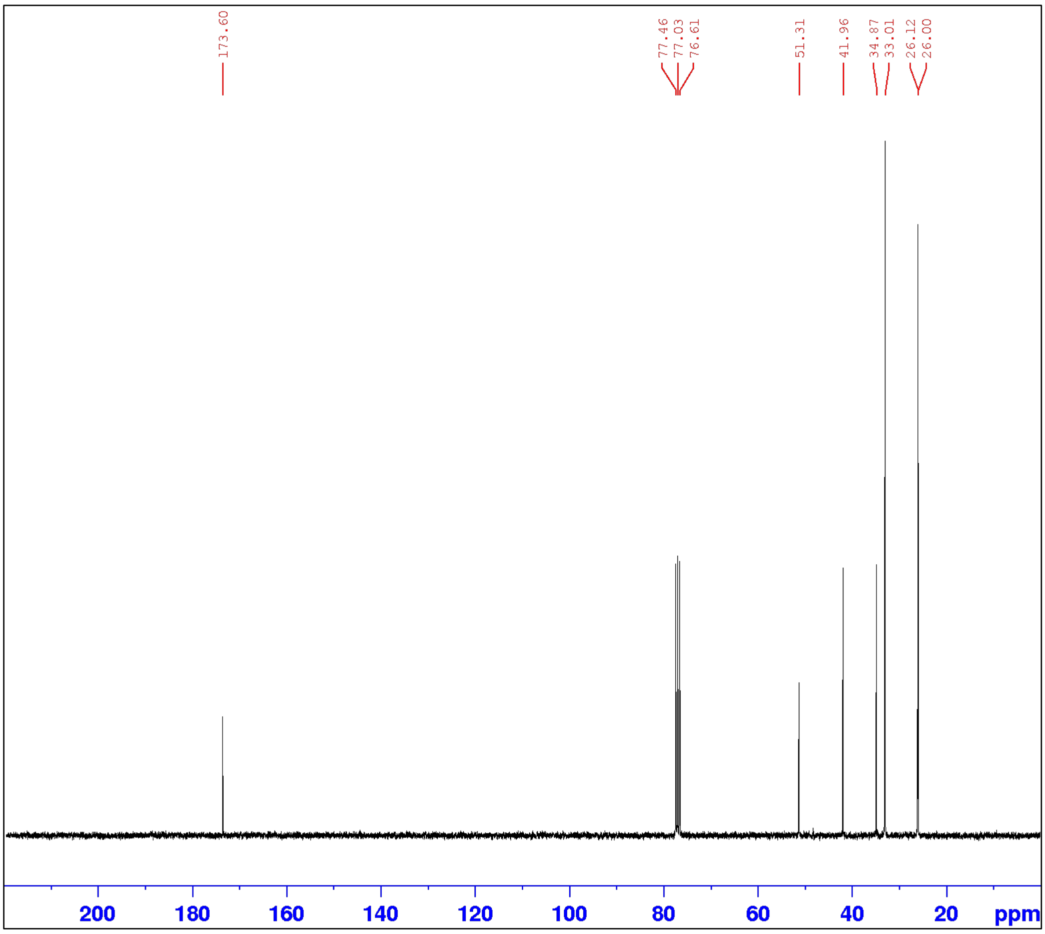


75 MHz, $CDCl_3$

300 MHz, $CDCl_3$

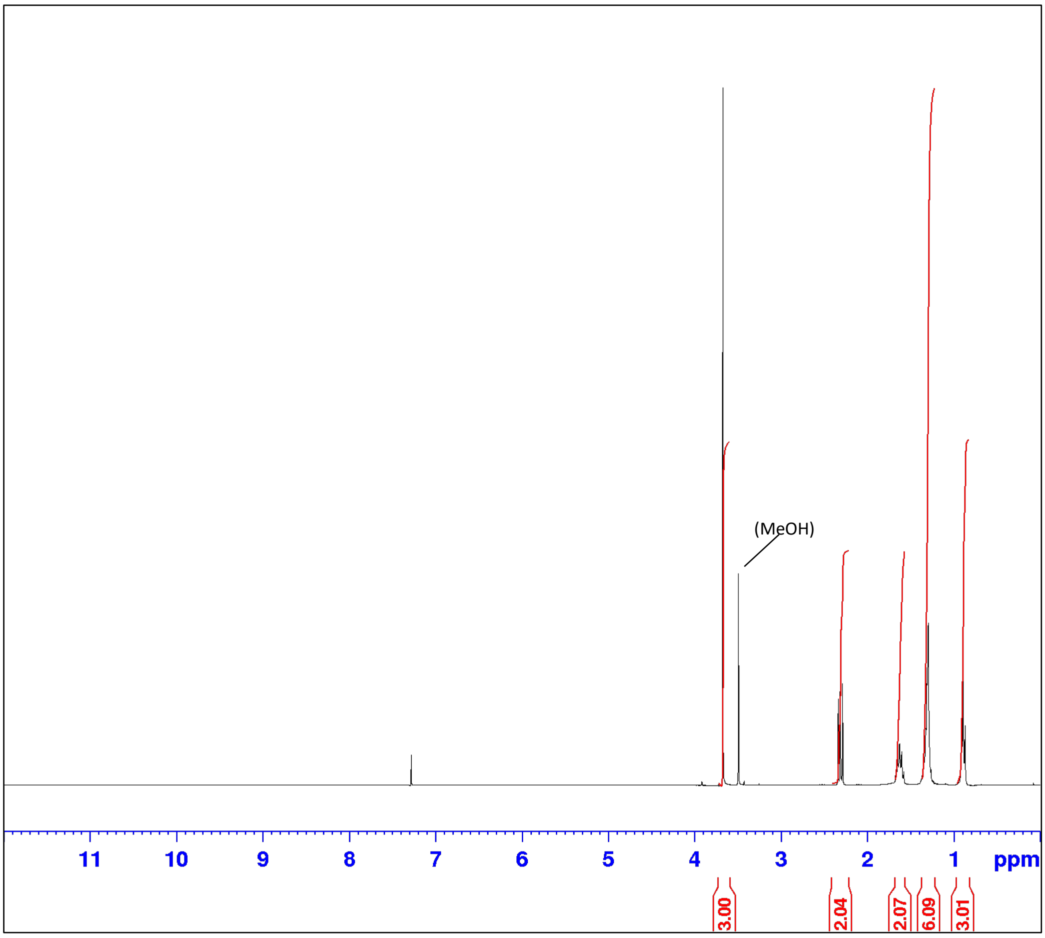


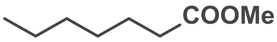


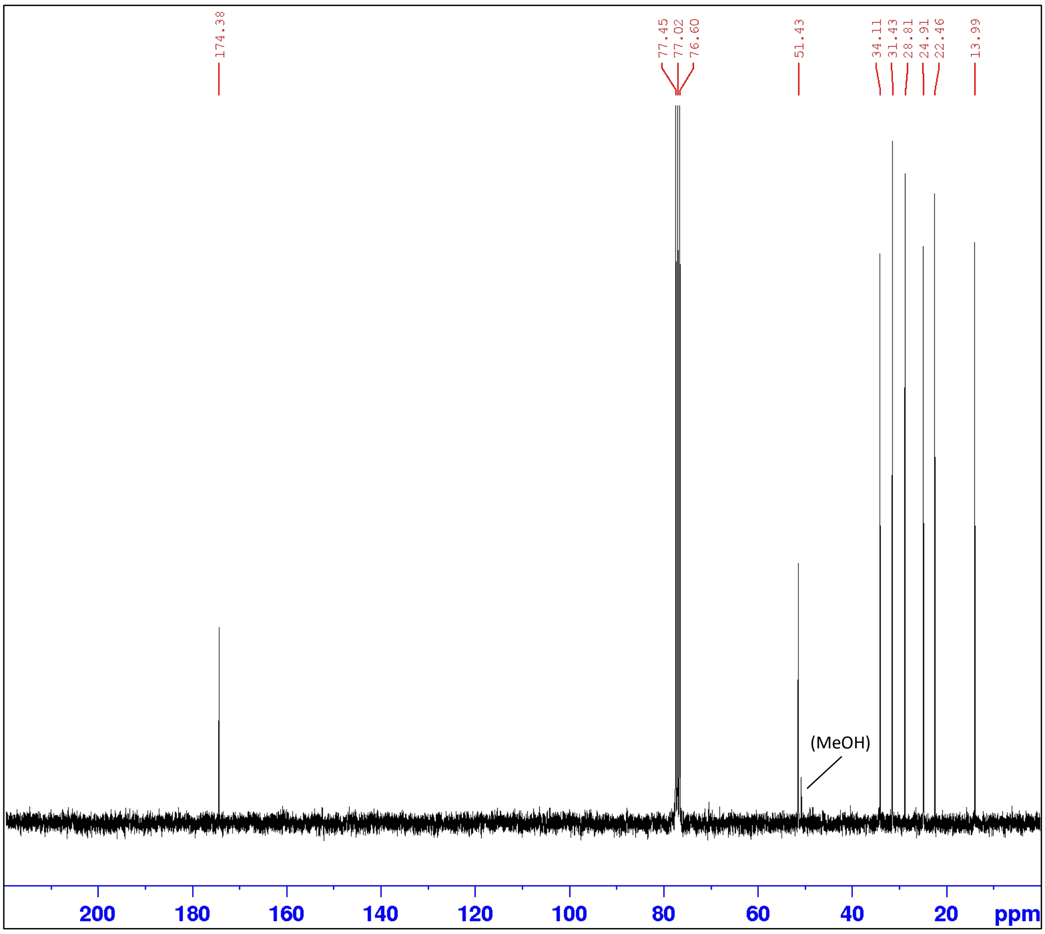


75 MHz, $CDCl_3$

300 MHz, $CDCl_3$

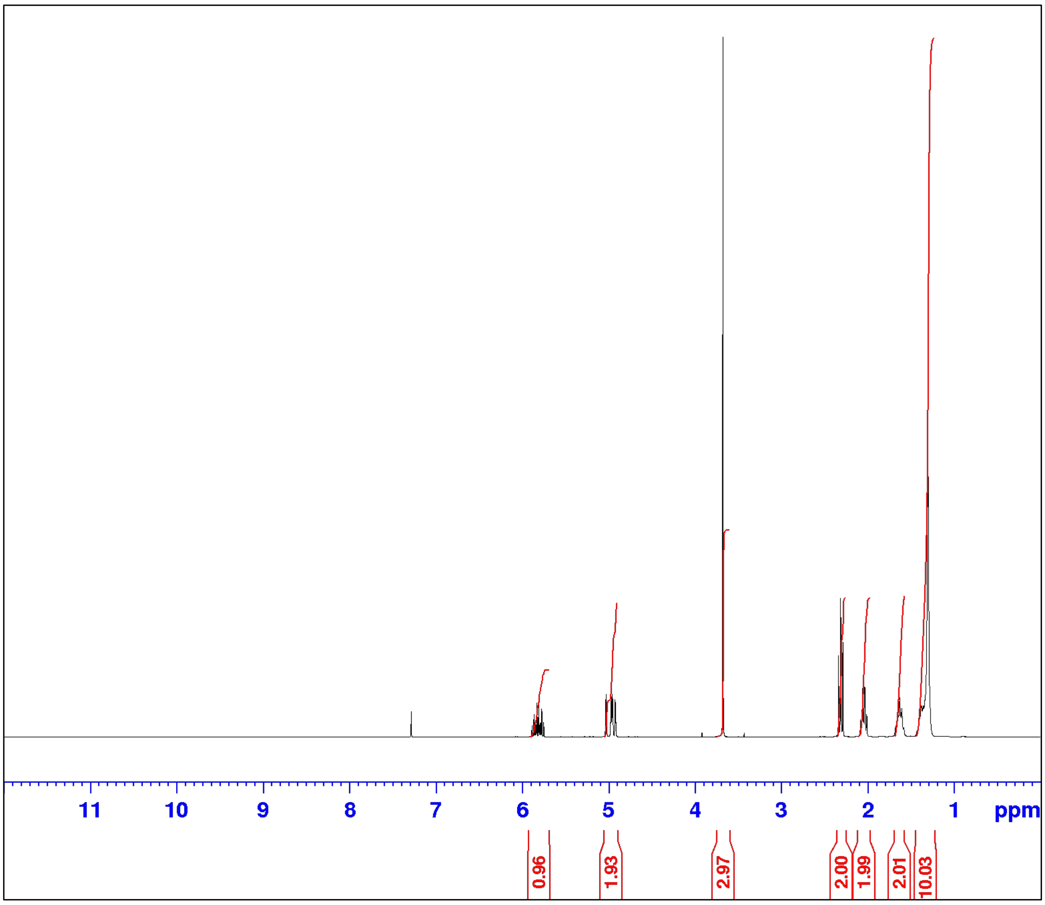


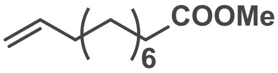


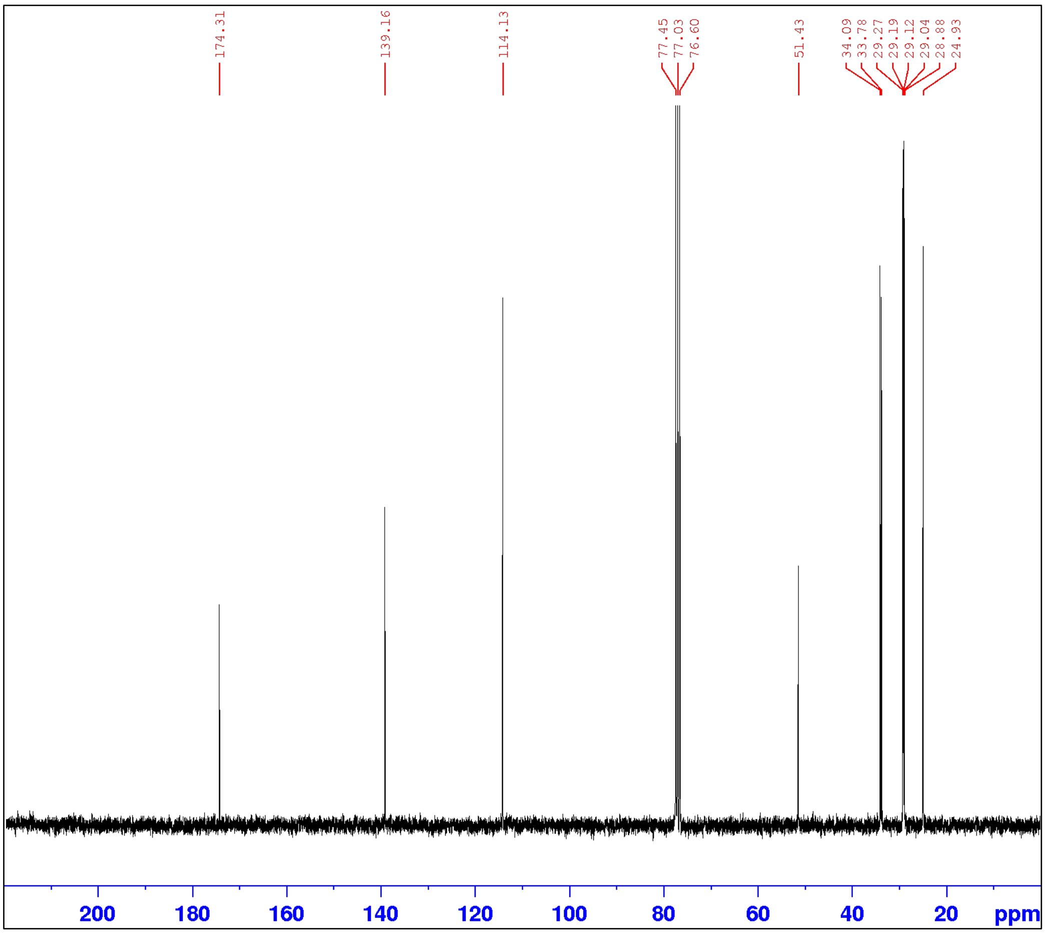


75 MHz, $CDCl_3$

300 MHz, $CDCl_3$

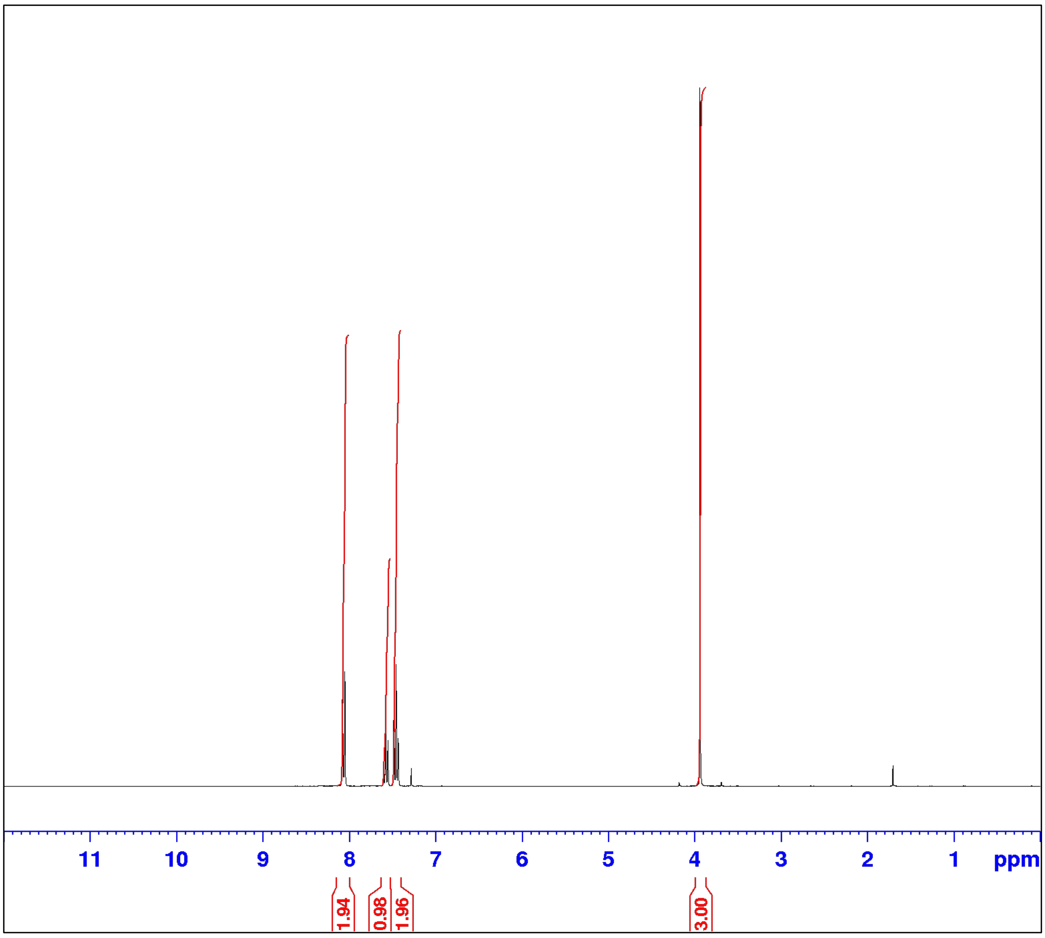


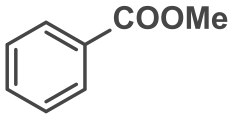


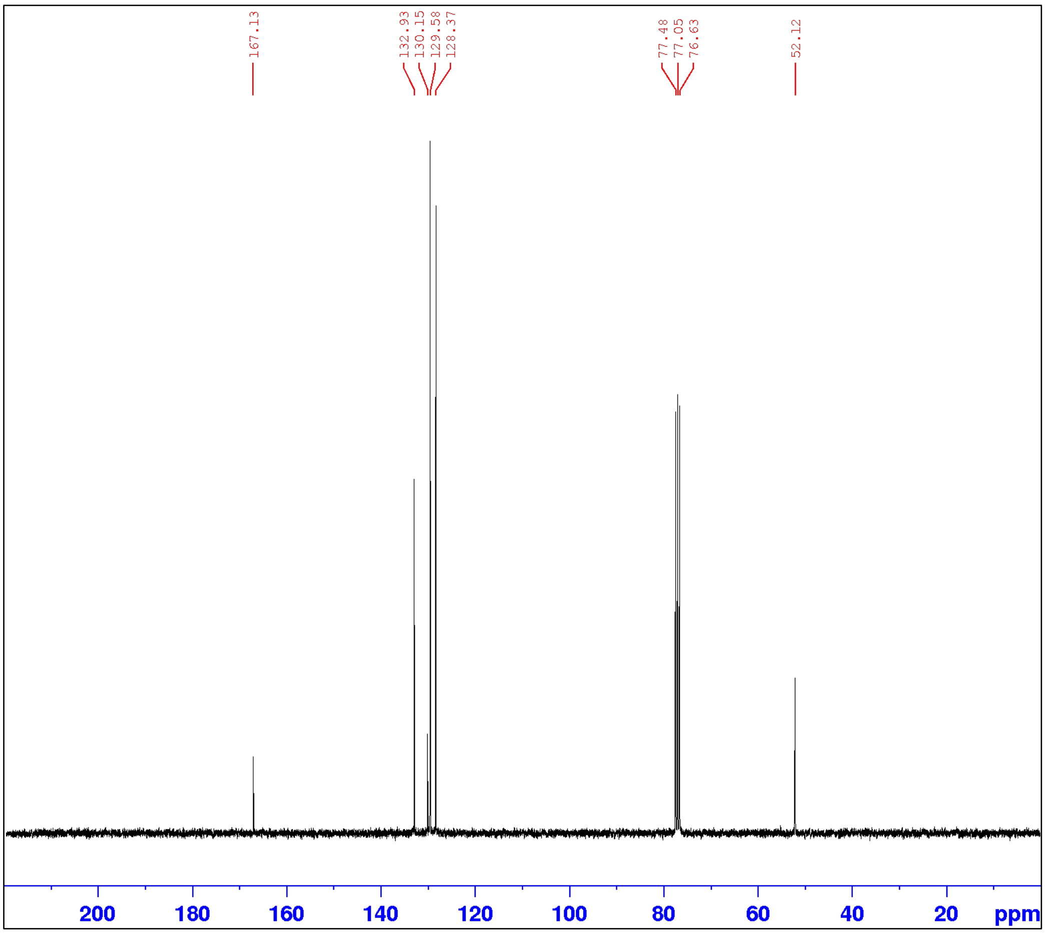


75 MHz, $CDCl_3$

300 MHz, $CDCl_3$

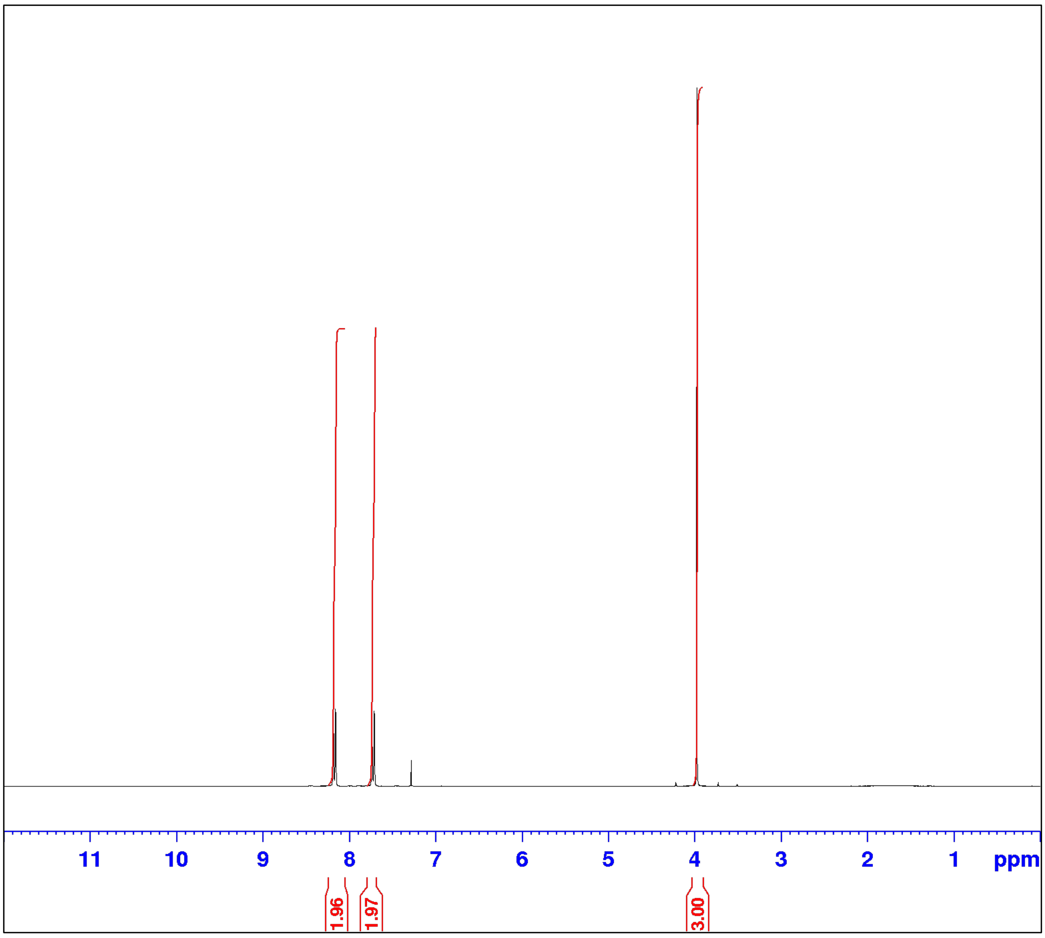


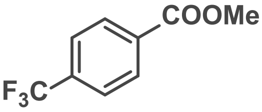


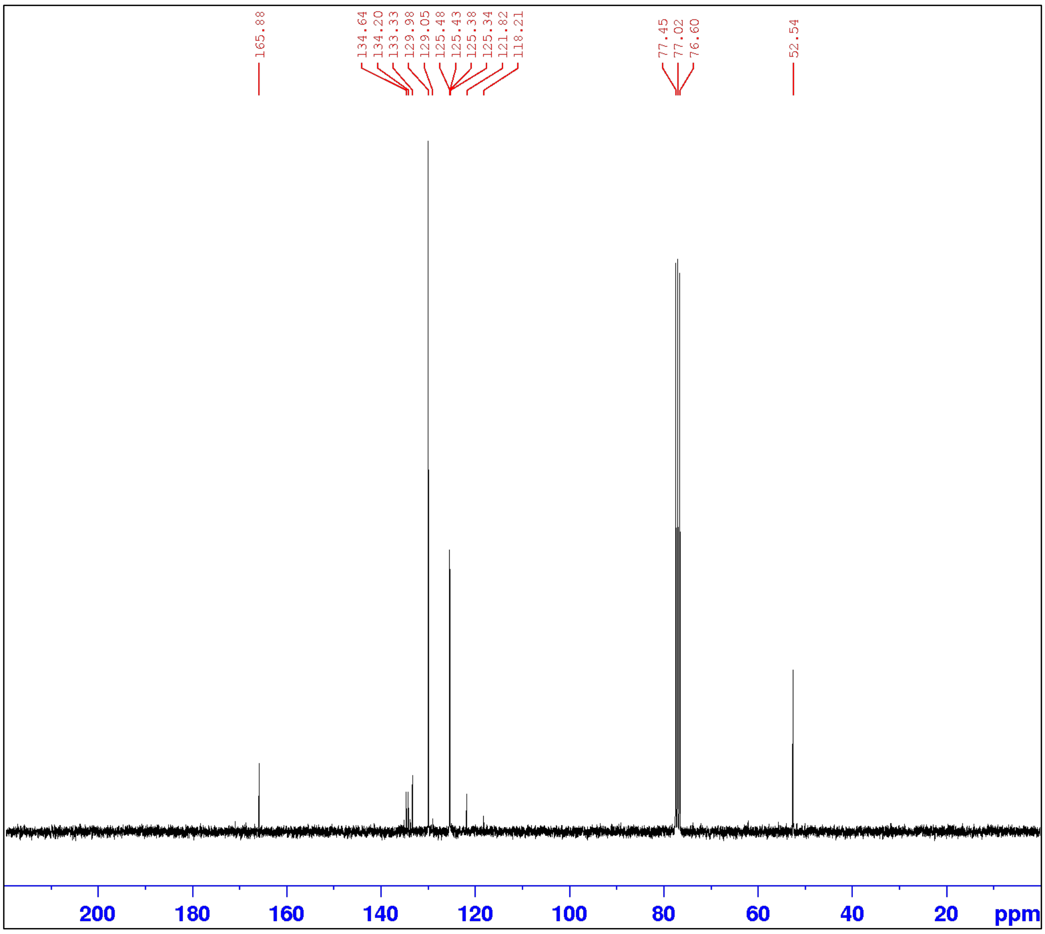


75 MHz, $CDCl_3$

300 MHz, CDCl$_3$

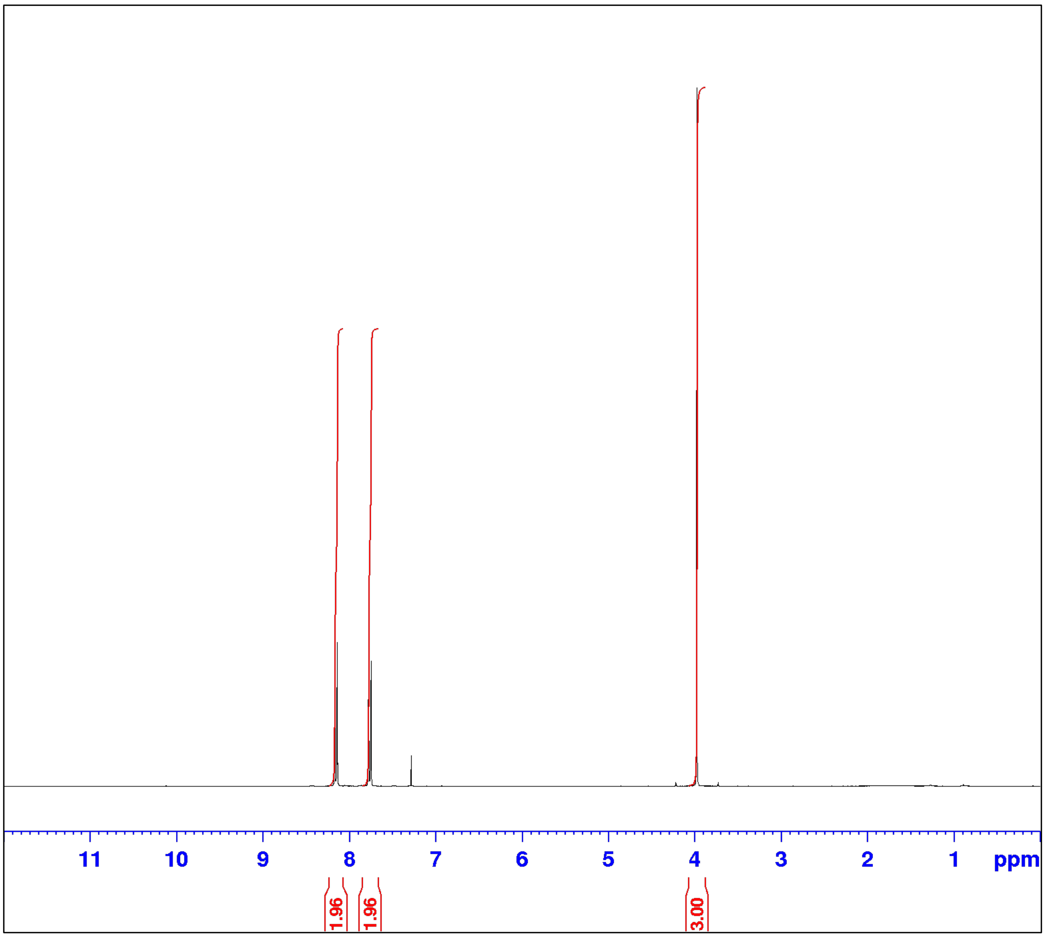


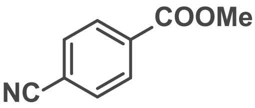


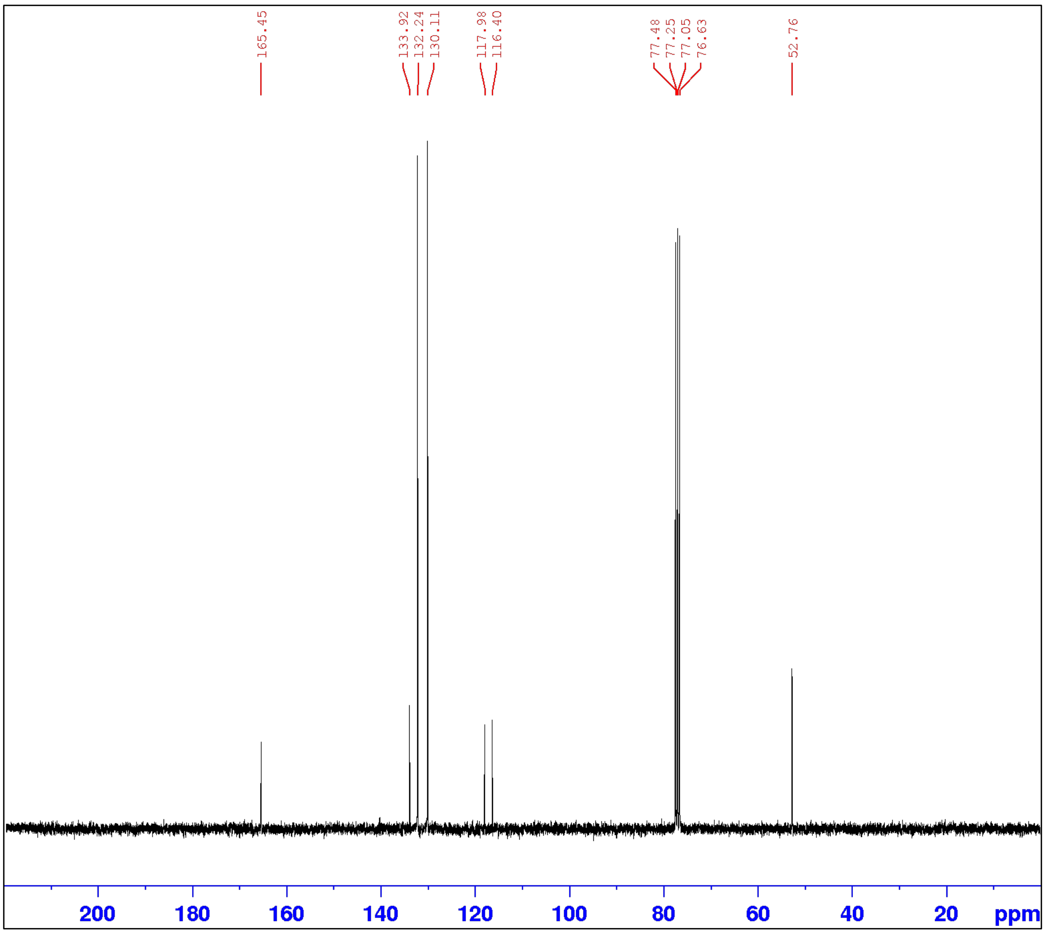


75 MHz, CDCl$_3$

300 MHz, $CDCl_3$

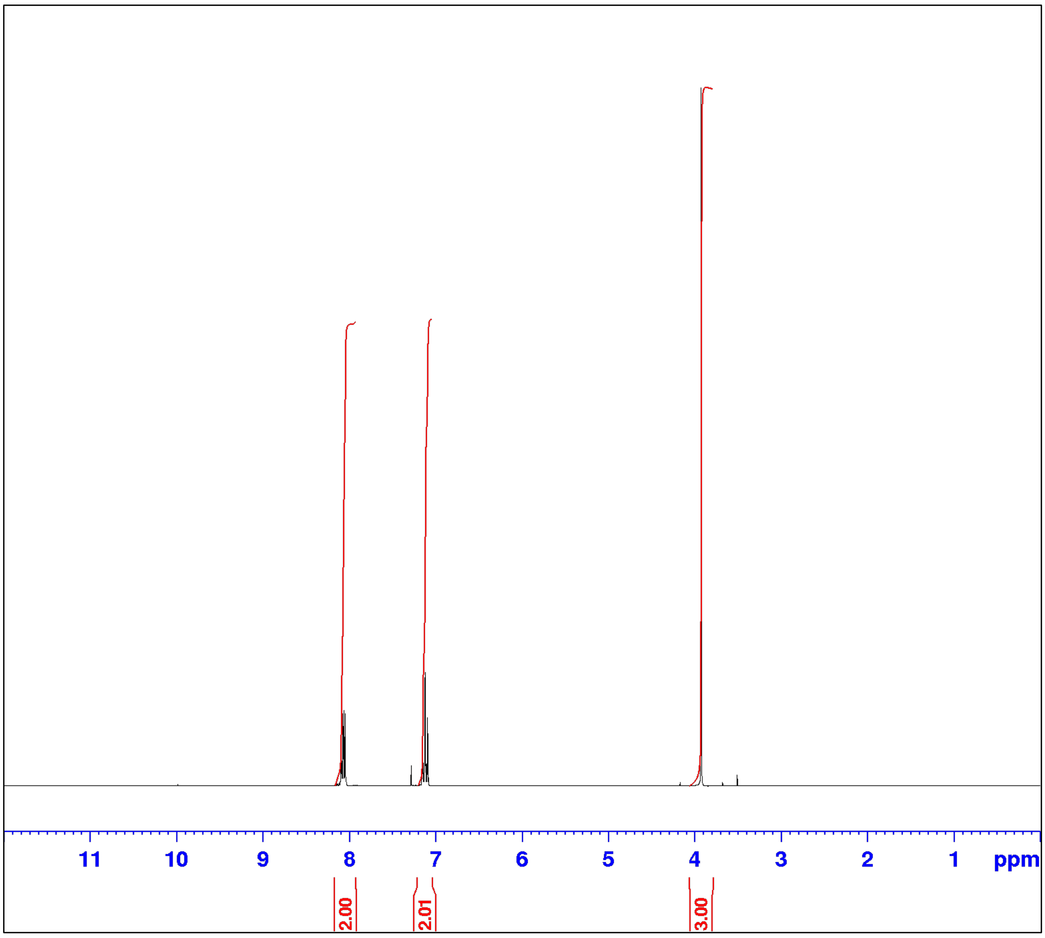


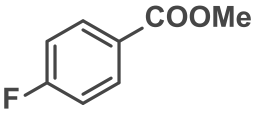


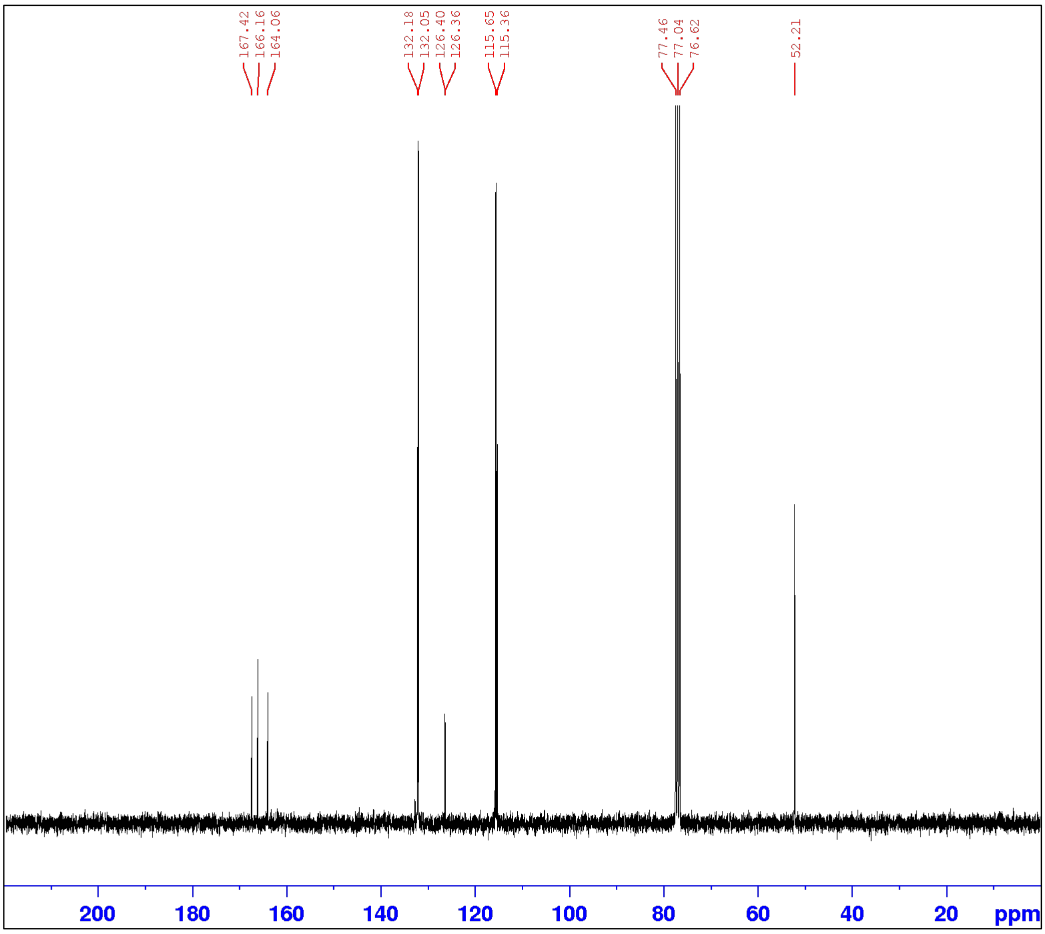


75 MHz, $CDCl_3$

300 MHz, $CDCl_3$

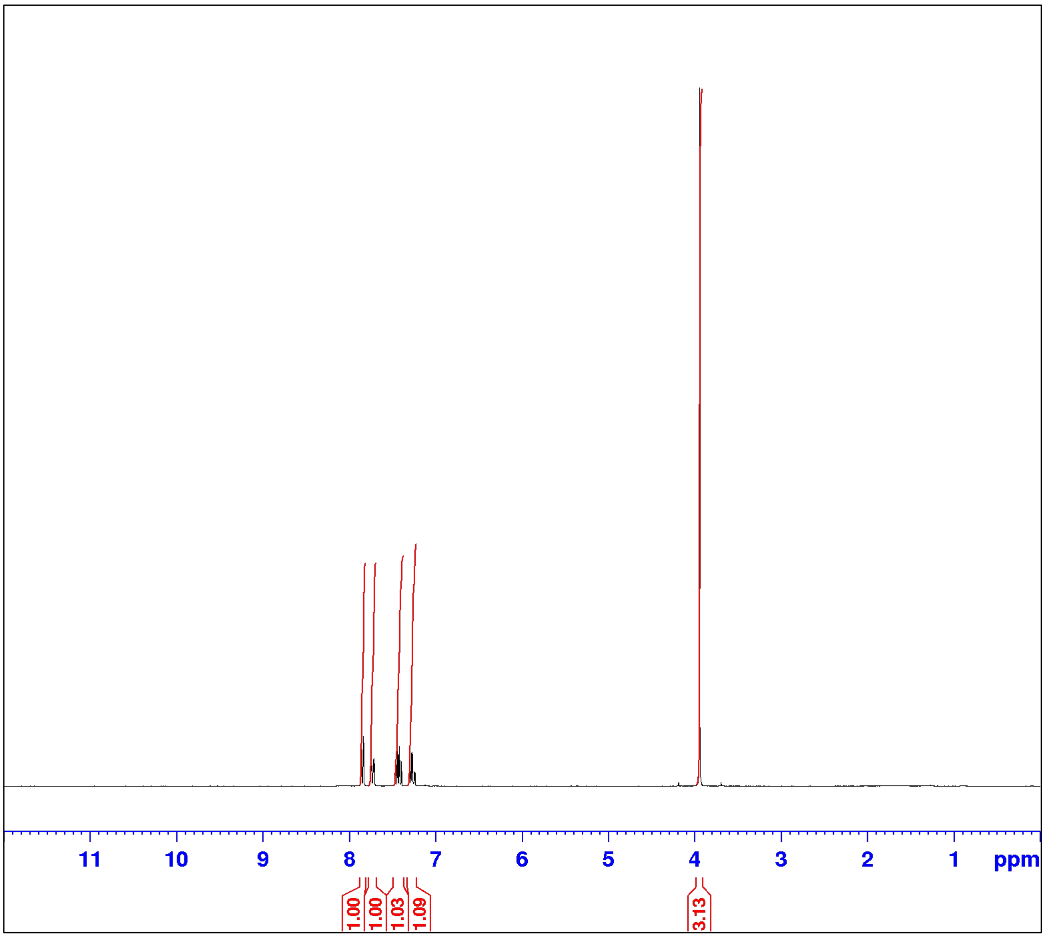


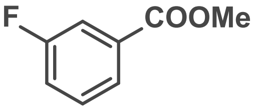


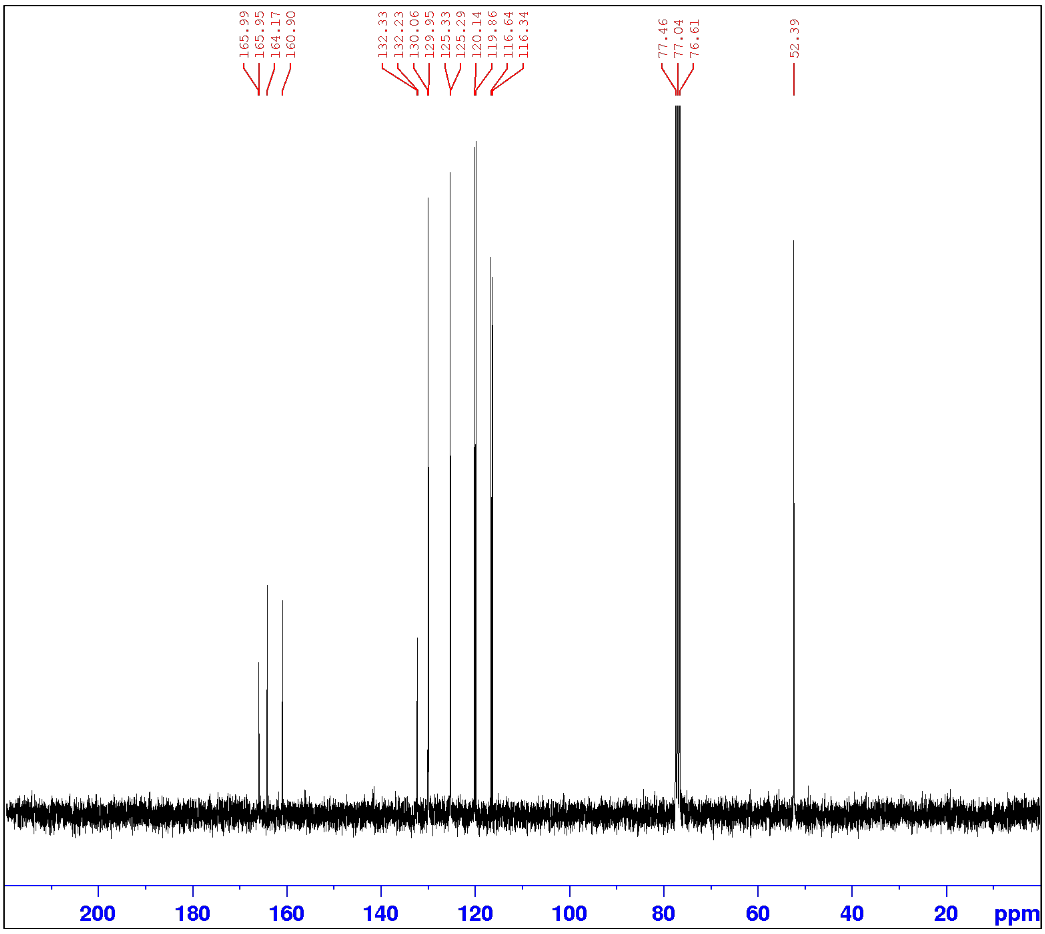


75 MHz, $CDCl_3$

300 MHz, $CDCl_3$

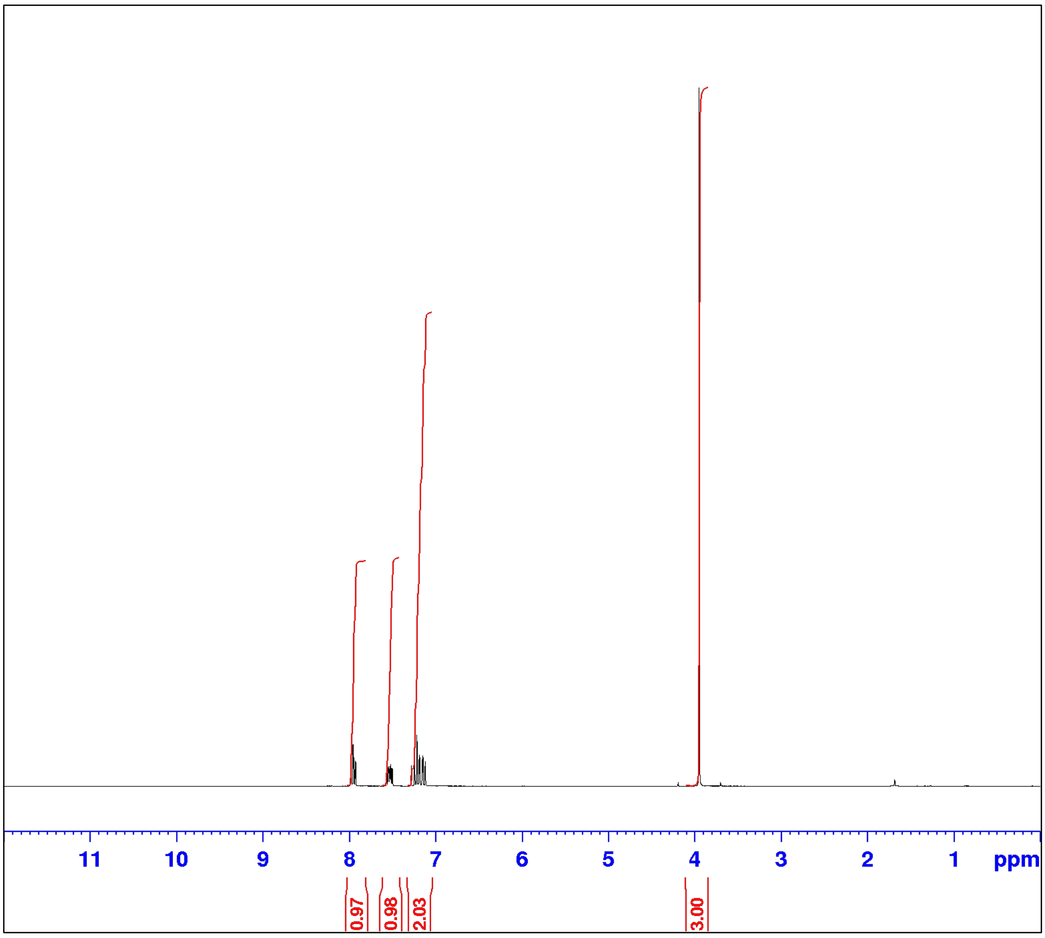


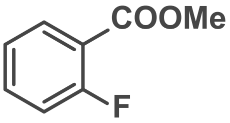


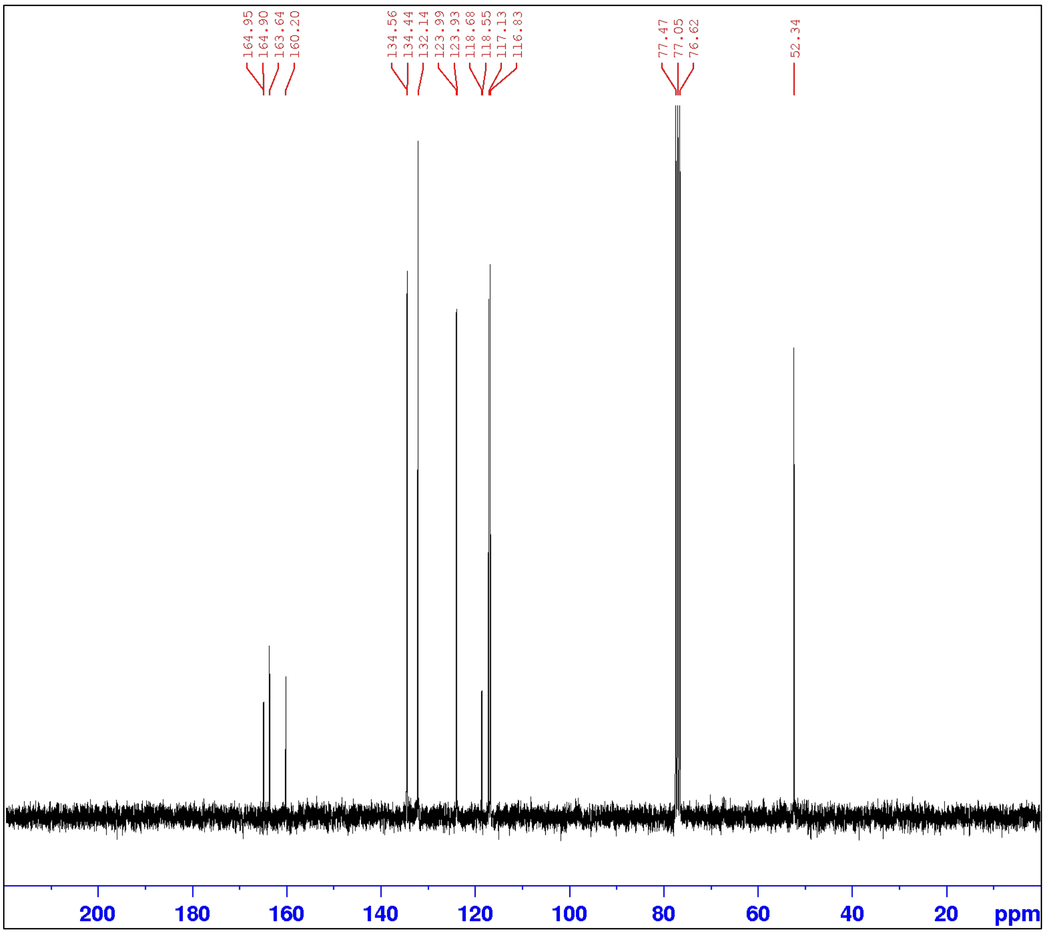


75 MHz, $CDCl_3$

300 MHz, CDCl3

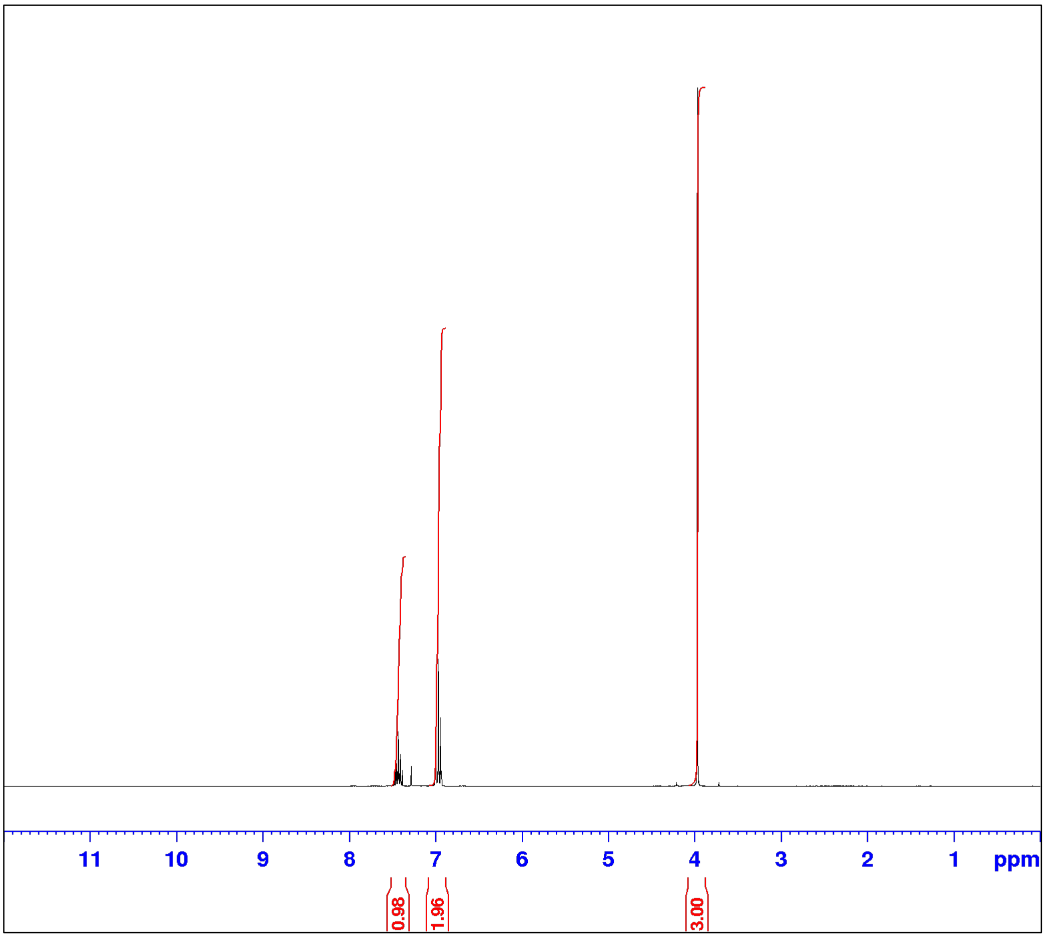


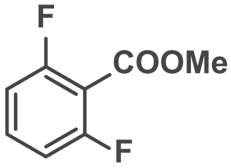


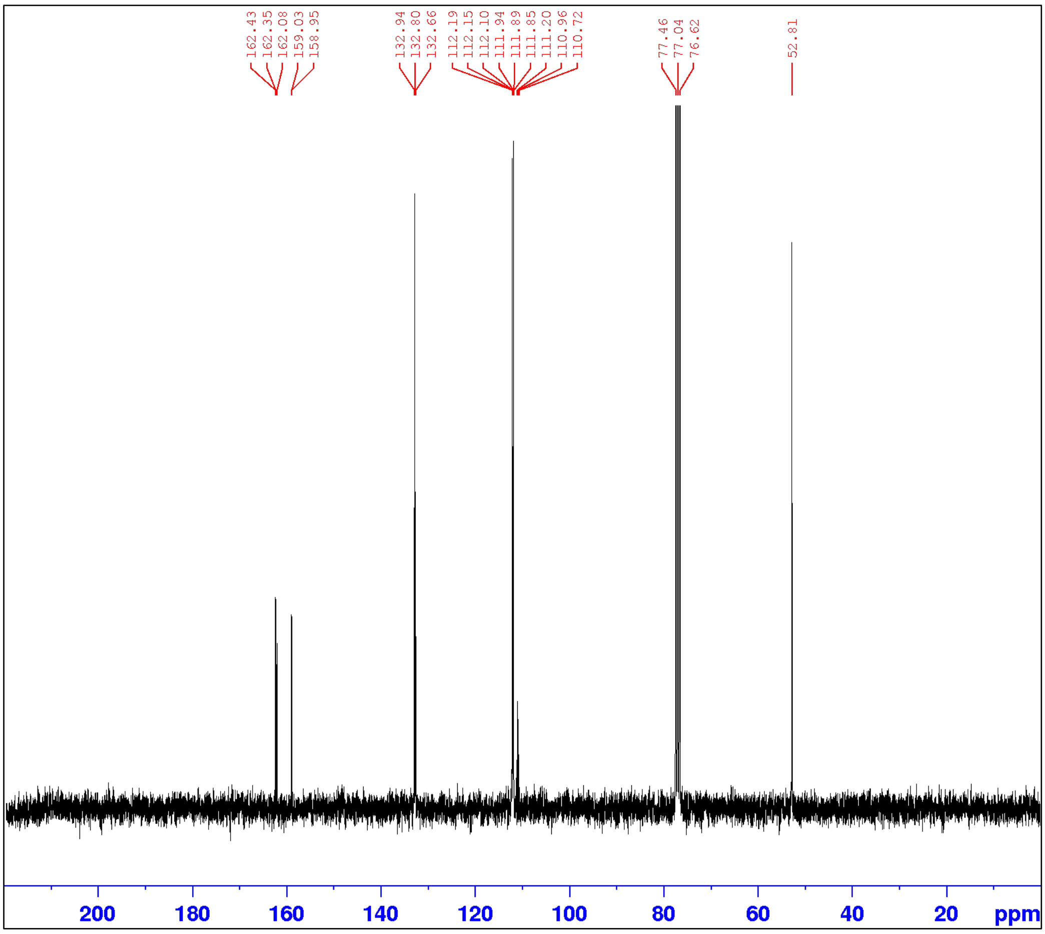


75 MHz, CDCl3

300 MHz, $CDCl_3$

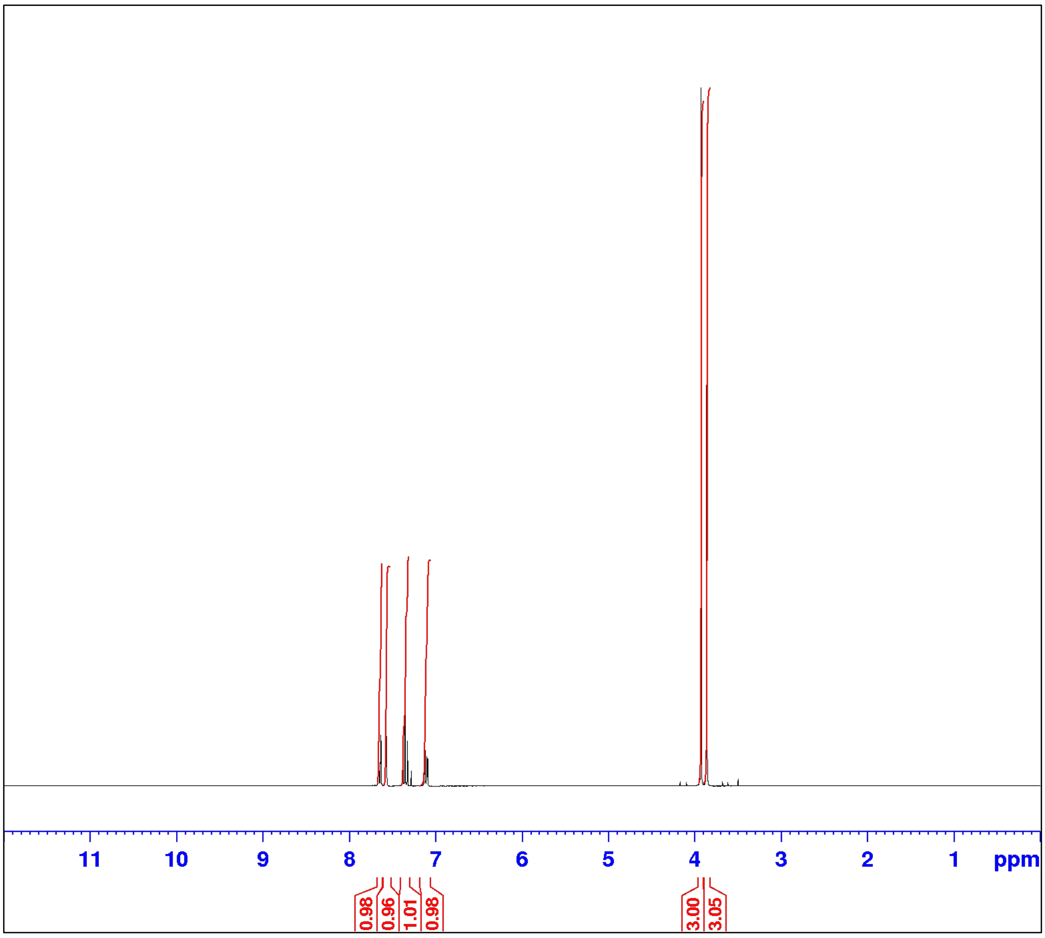


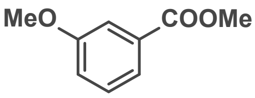


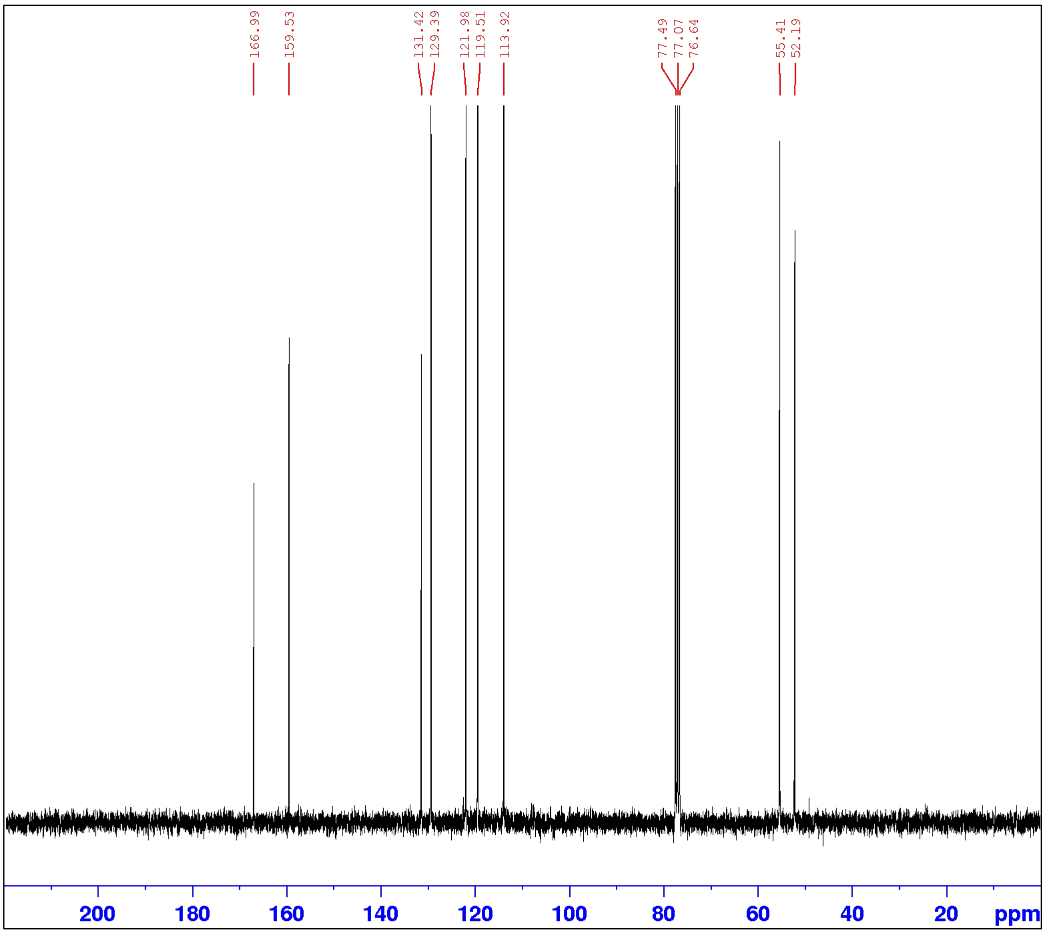


75 MHz, $CDCl_3$

300 MHz, $CDCl_3$

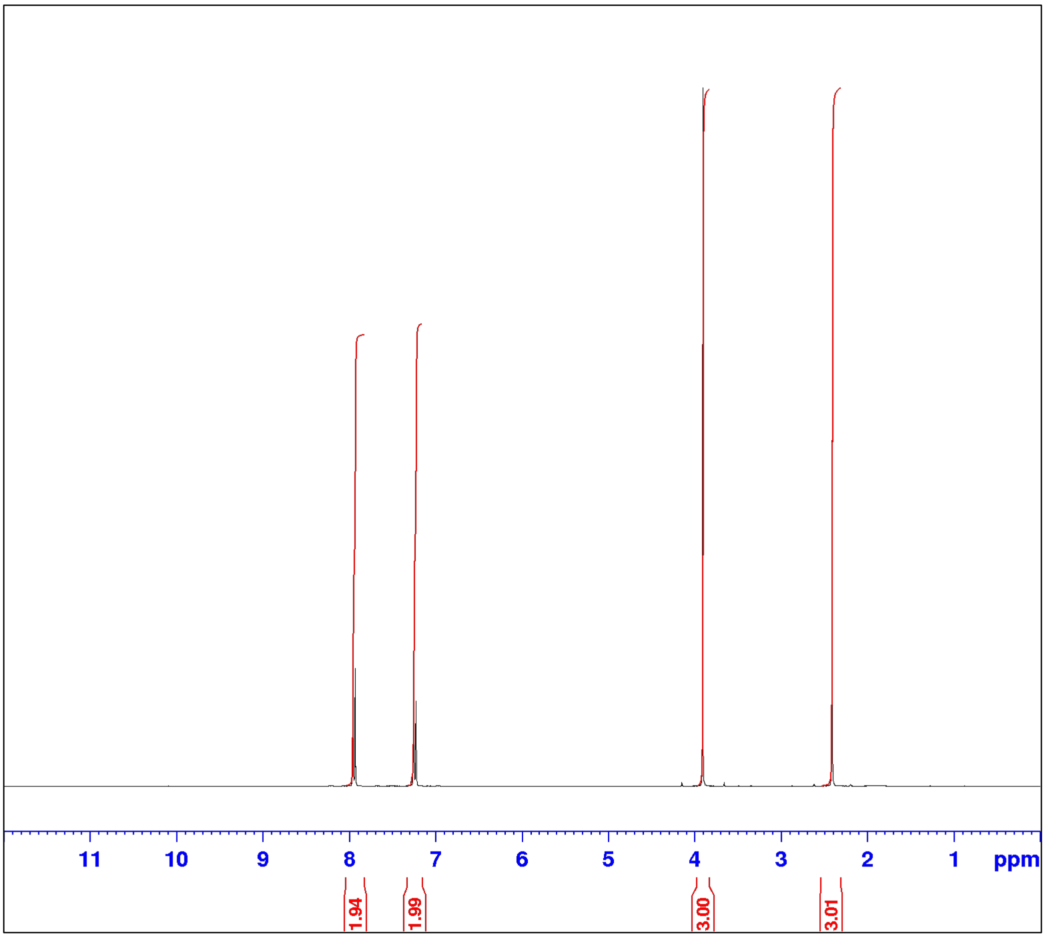


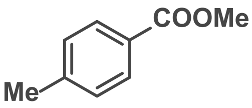


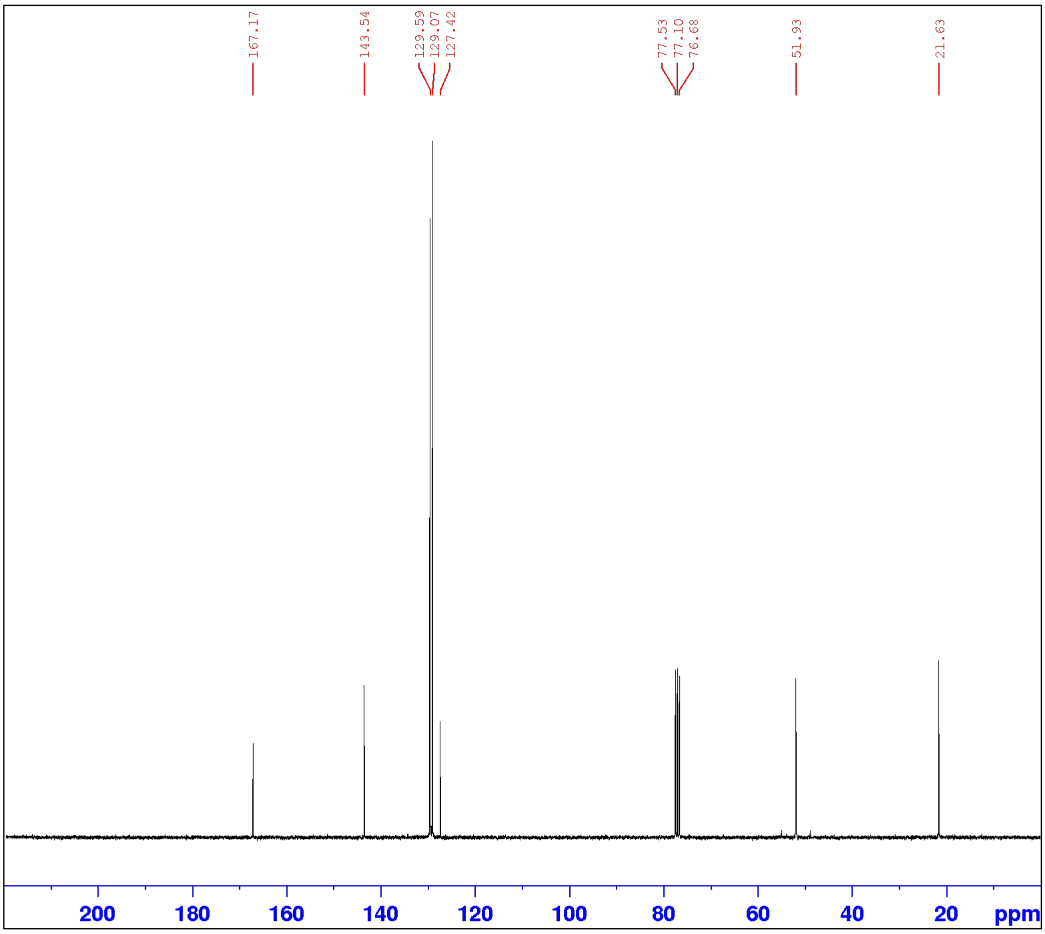


75 MHz, $CDCl_3$

300 MHz, $CDCl_3$

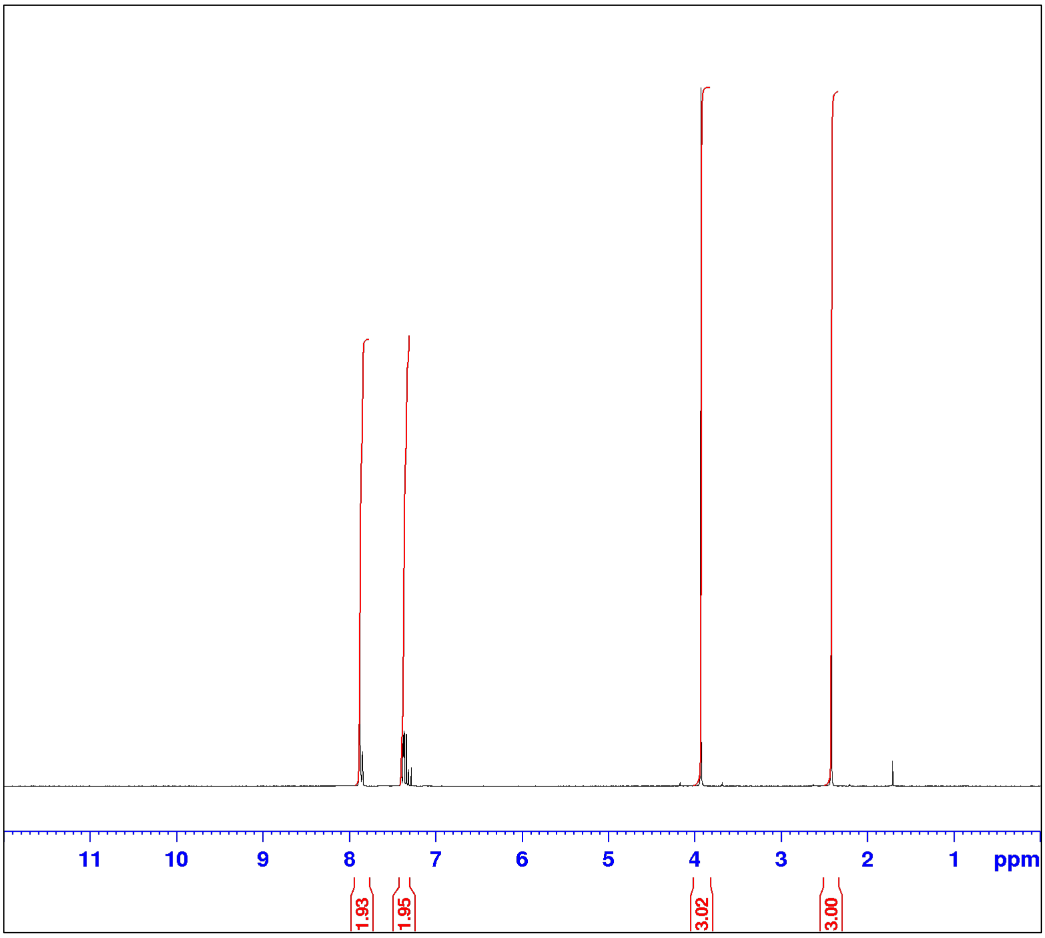


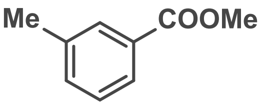


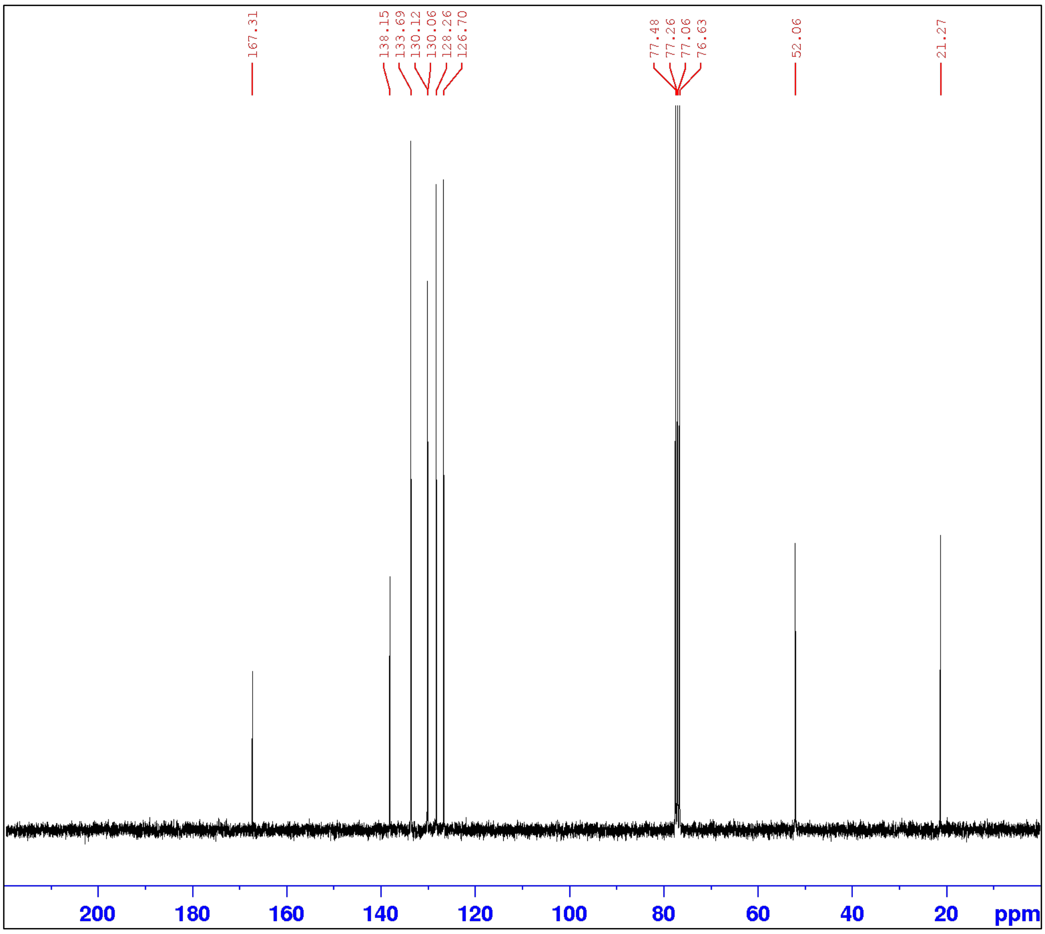


75 MHz, $CDCl_3$

300 MHz, $CDCl_3$

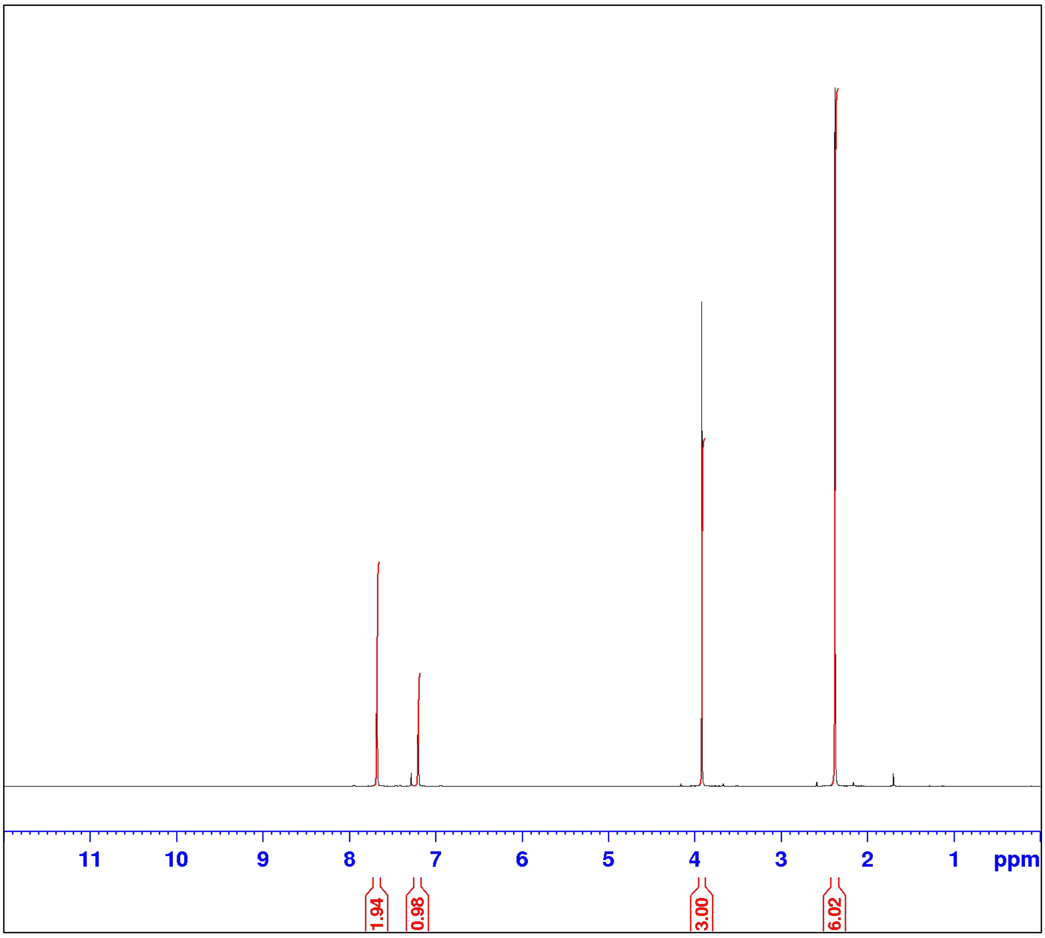


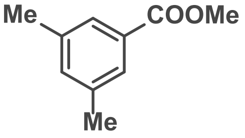


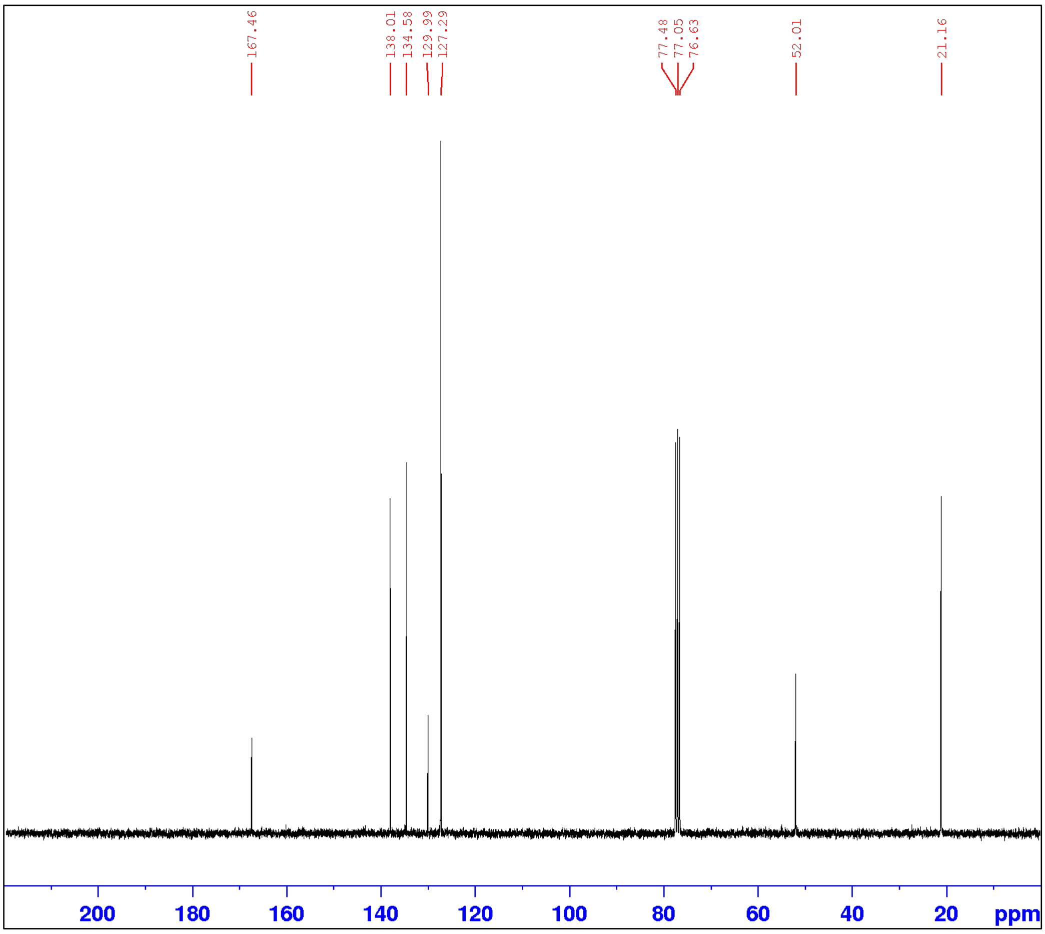


75 MHz, $CDCl_3$

300 MHz, $CDCl_3$

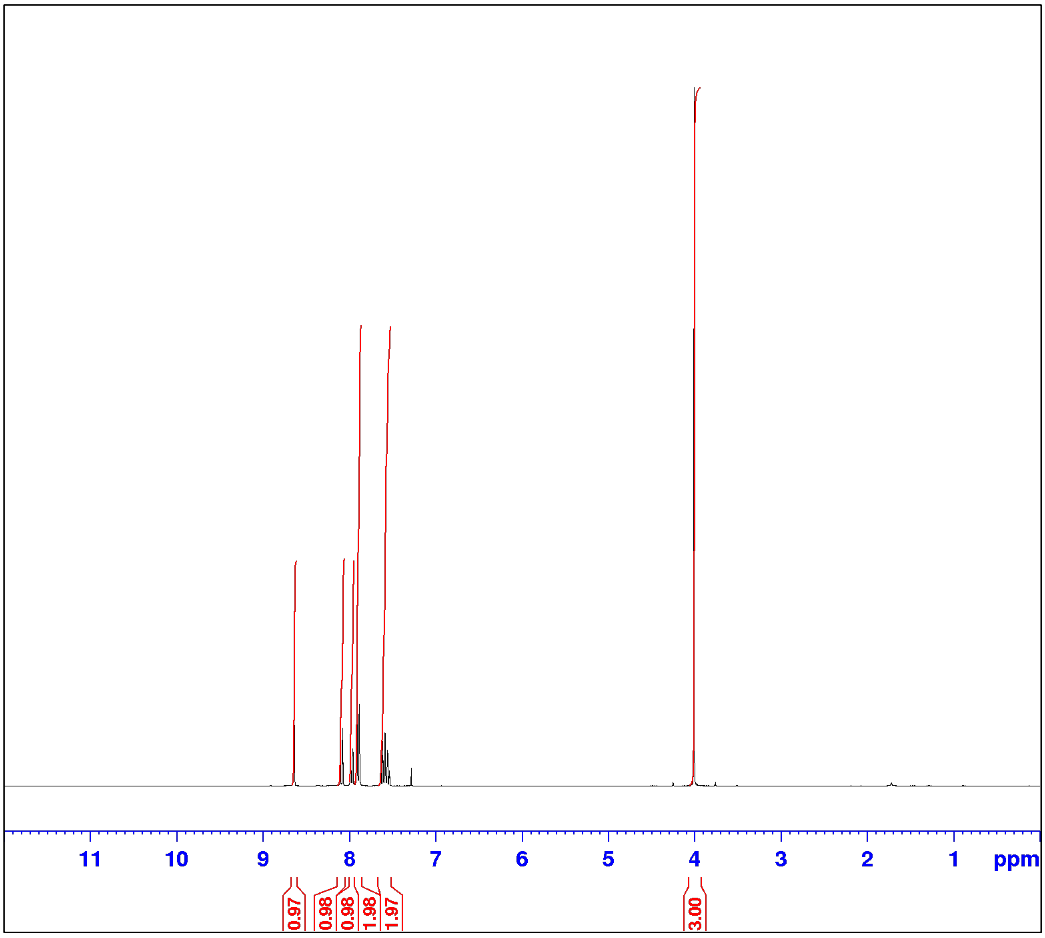


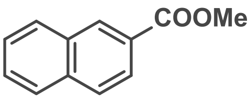


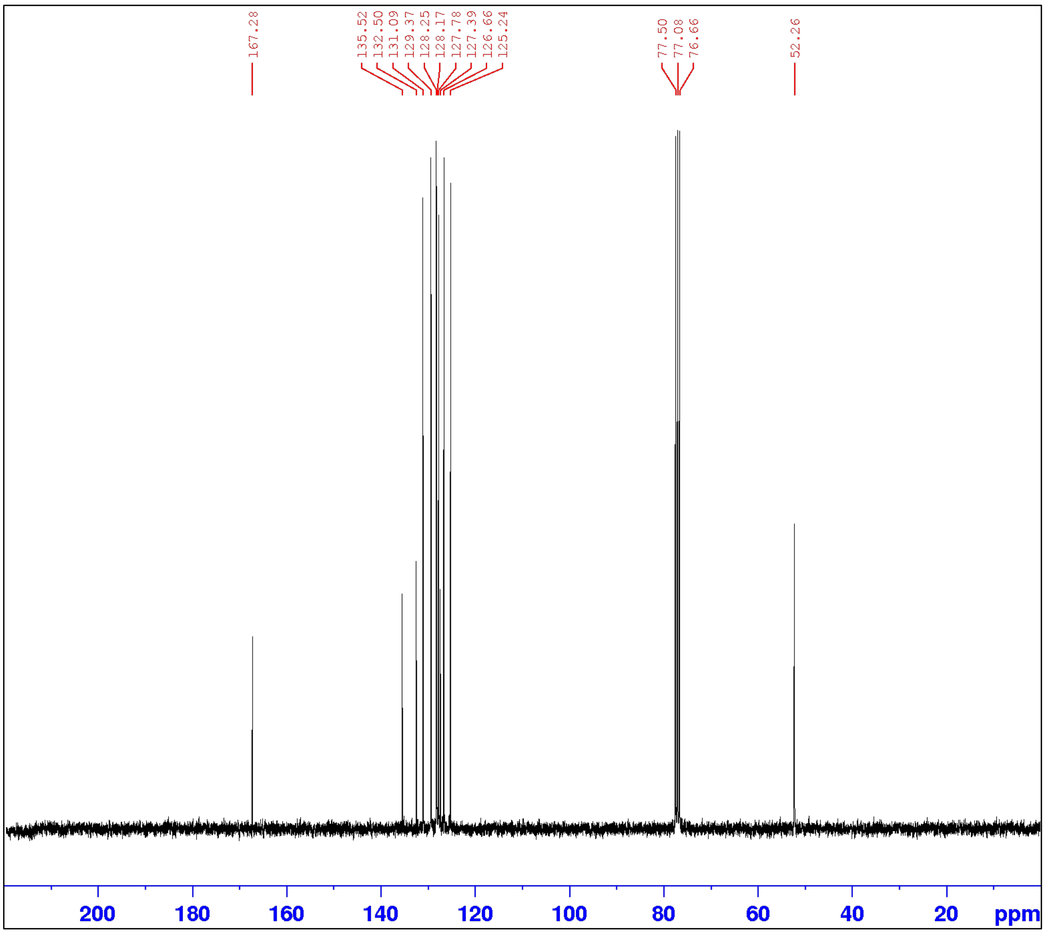


75 MHz, $CDCl_3$

300 MHz, $CDCl_3$

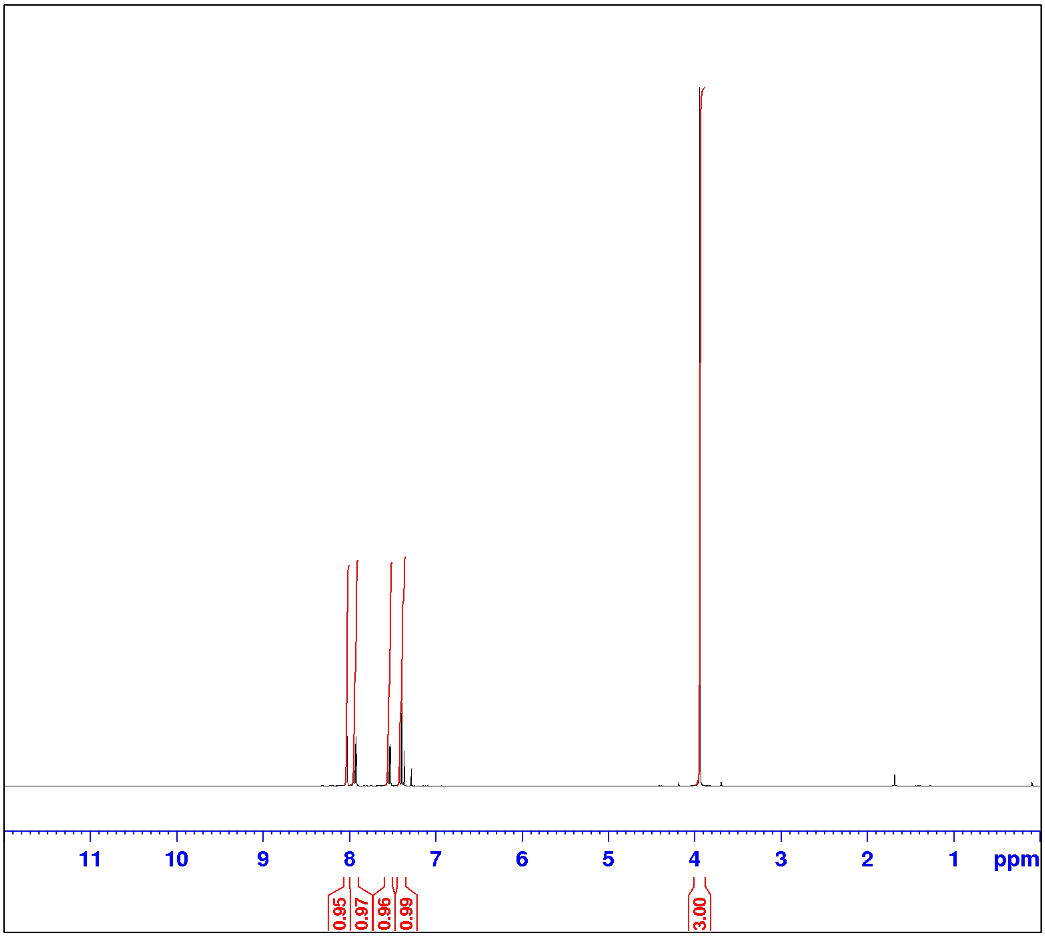


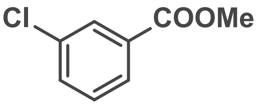


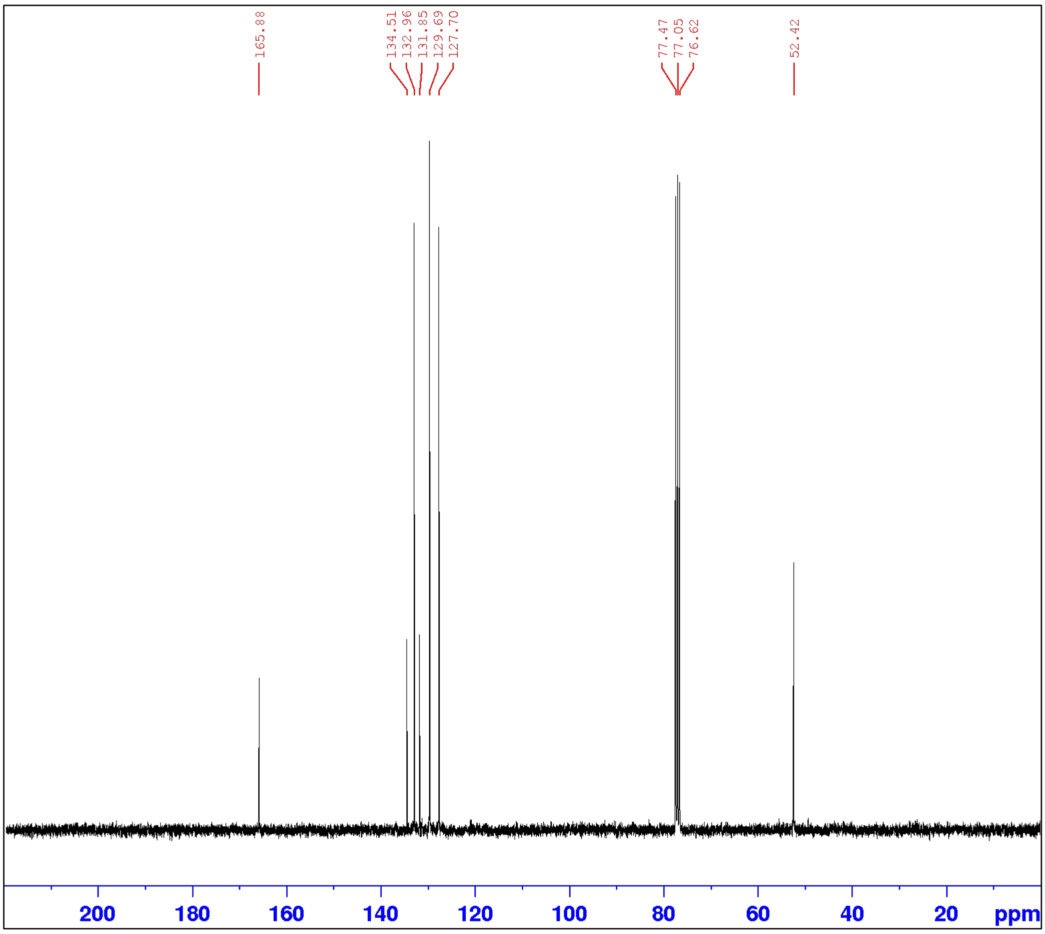


75 MHz, $CDCl_3$

300 MHz, $CDCl_3$

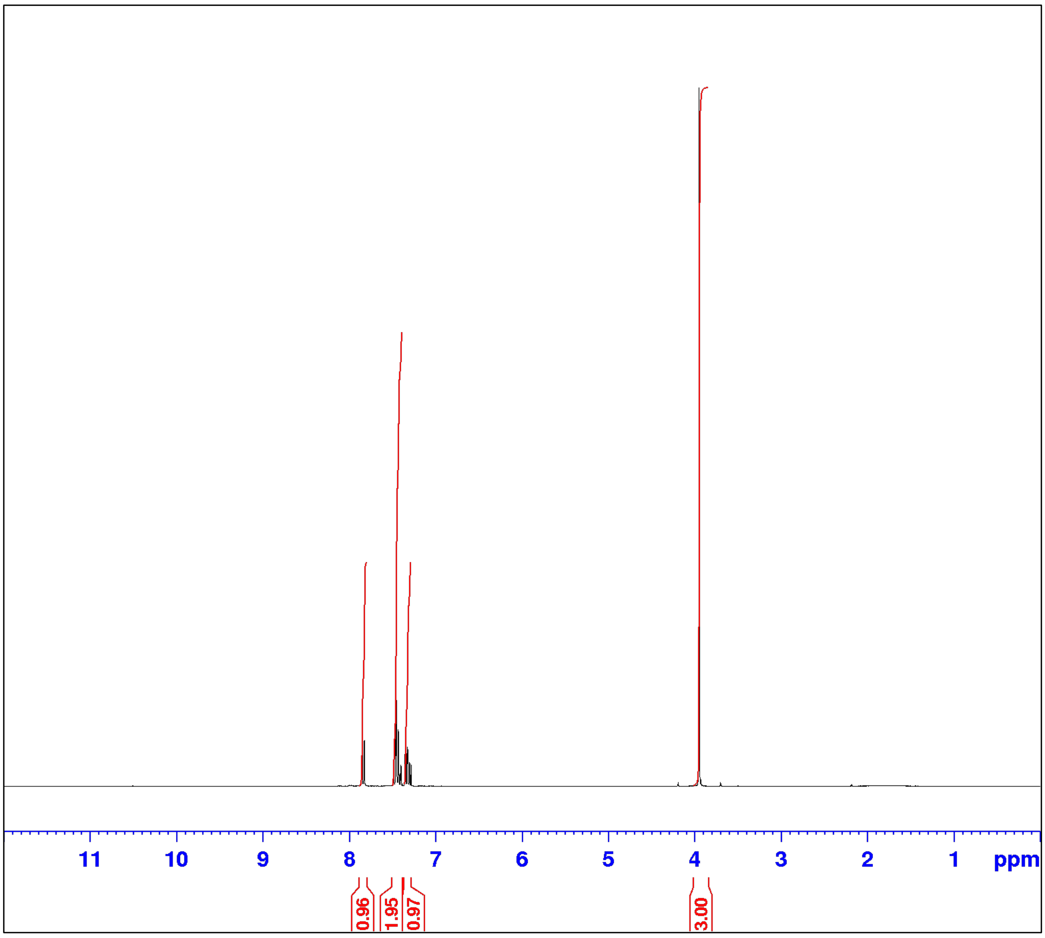


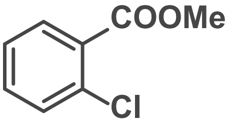


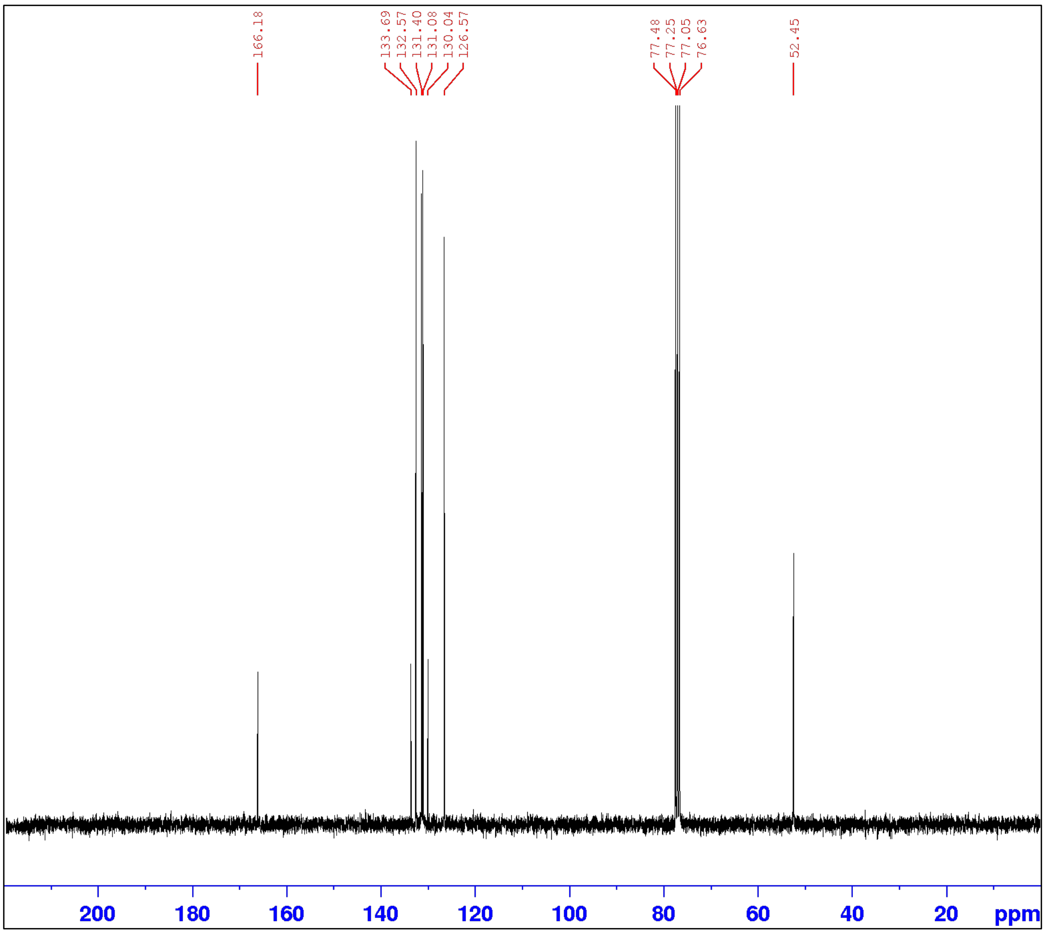


75 MHz, $CDCl_3$

300 MHz, $CDCl_3$

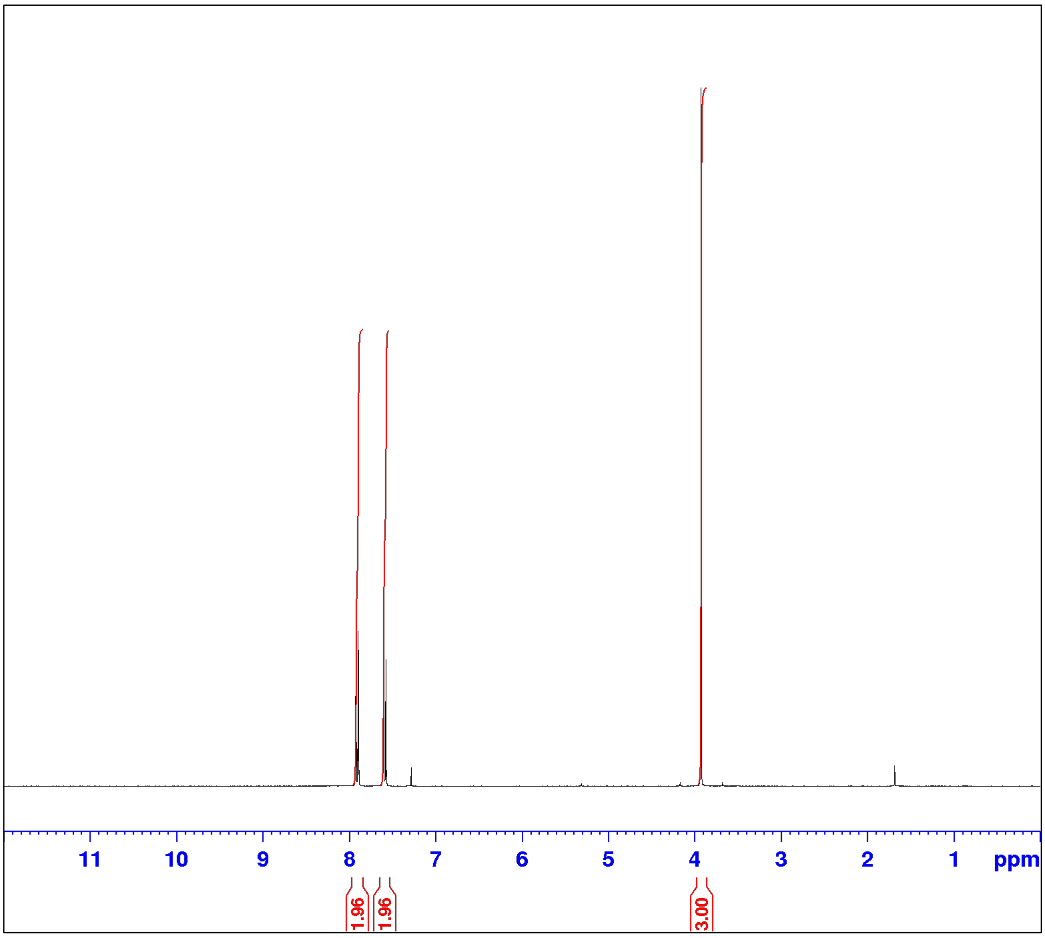


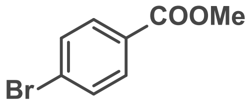


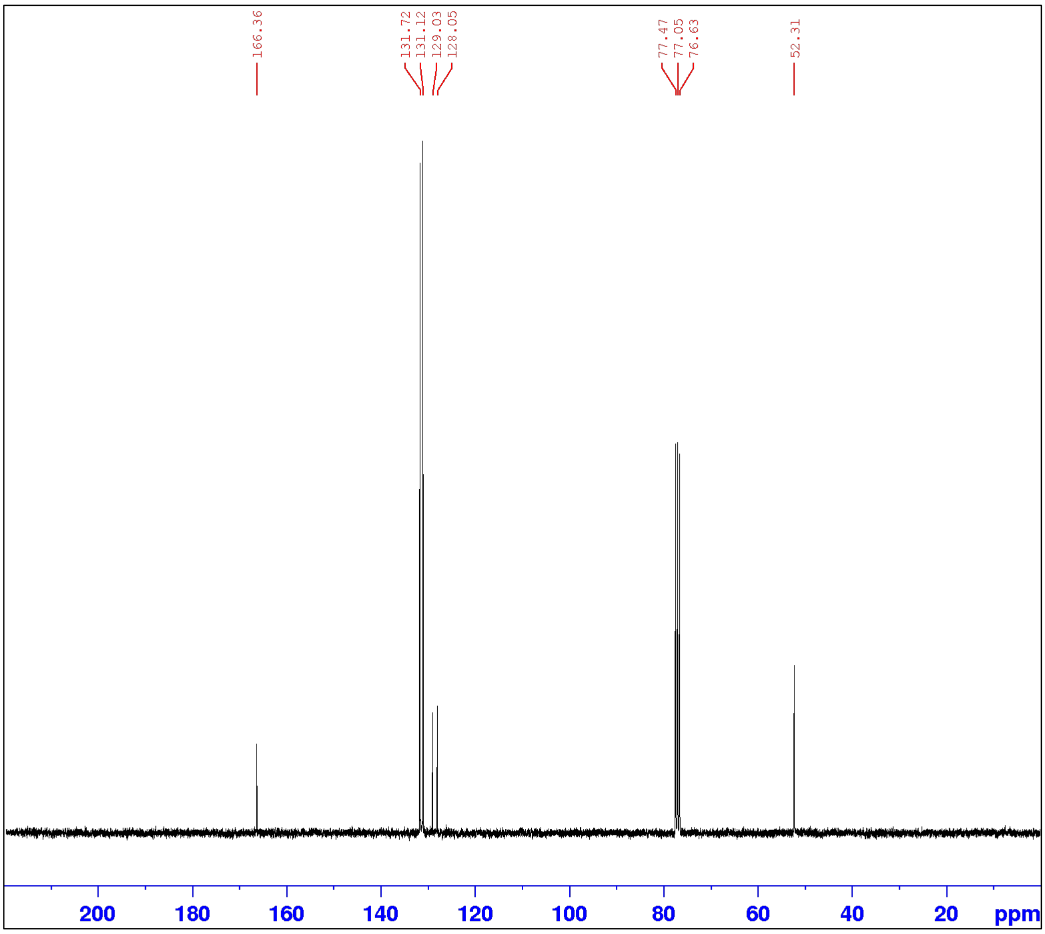


75 MHz, $CDCl_3$

300 MHz, $CDCl_3$

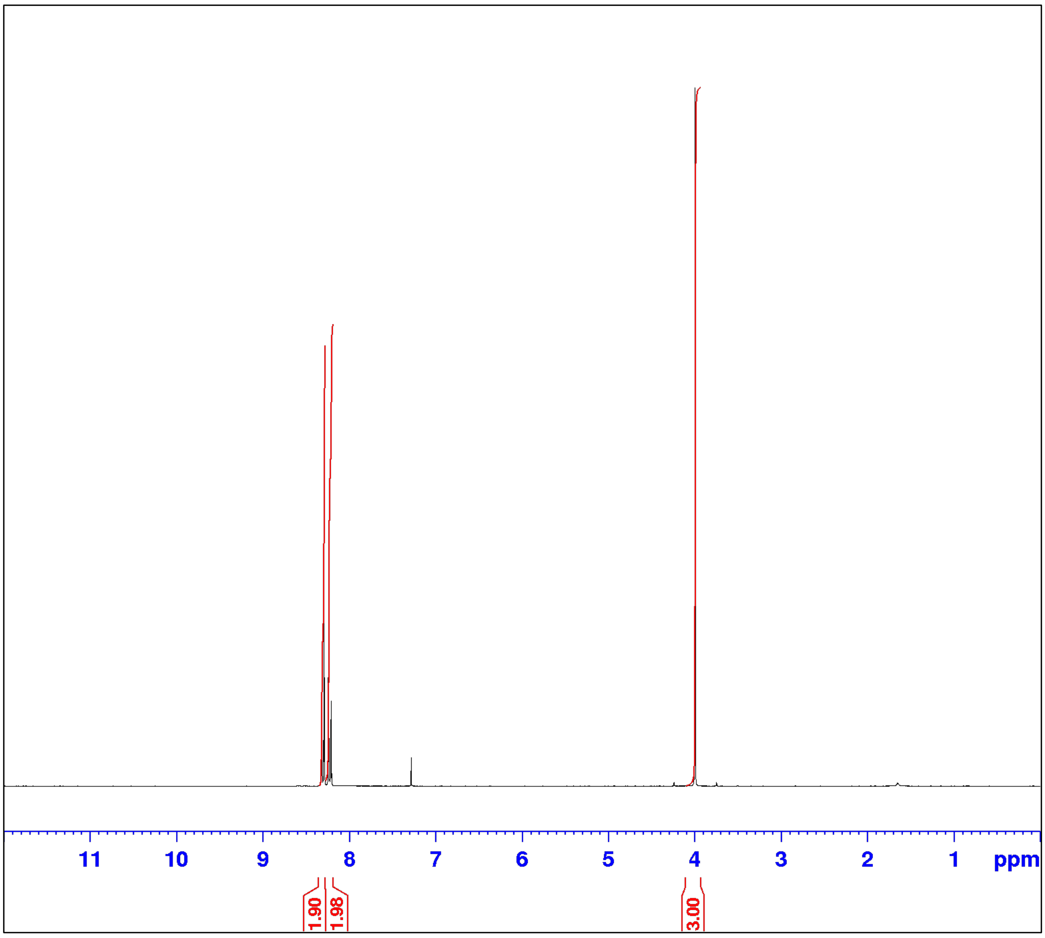


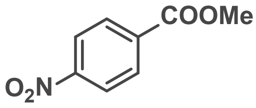


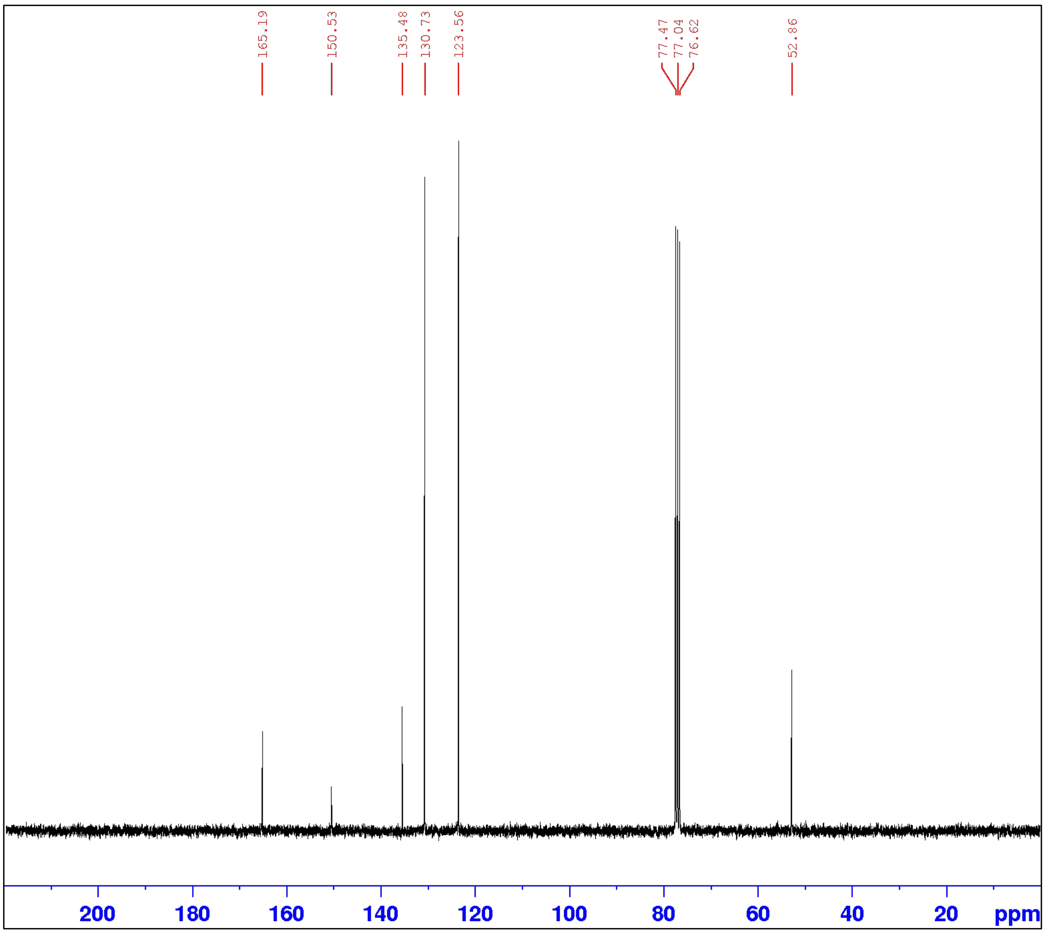


75 MHz, $CDCl_3$

300 MHz, $CDCl_3$

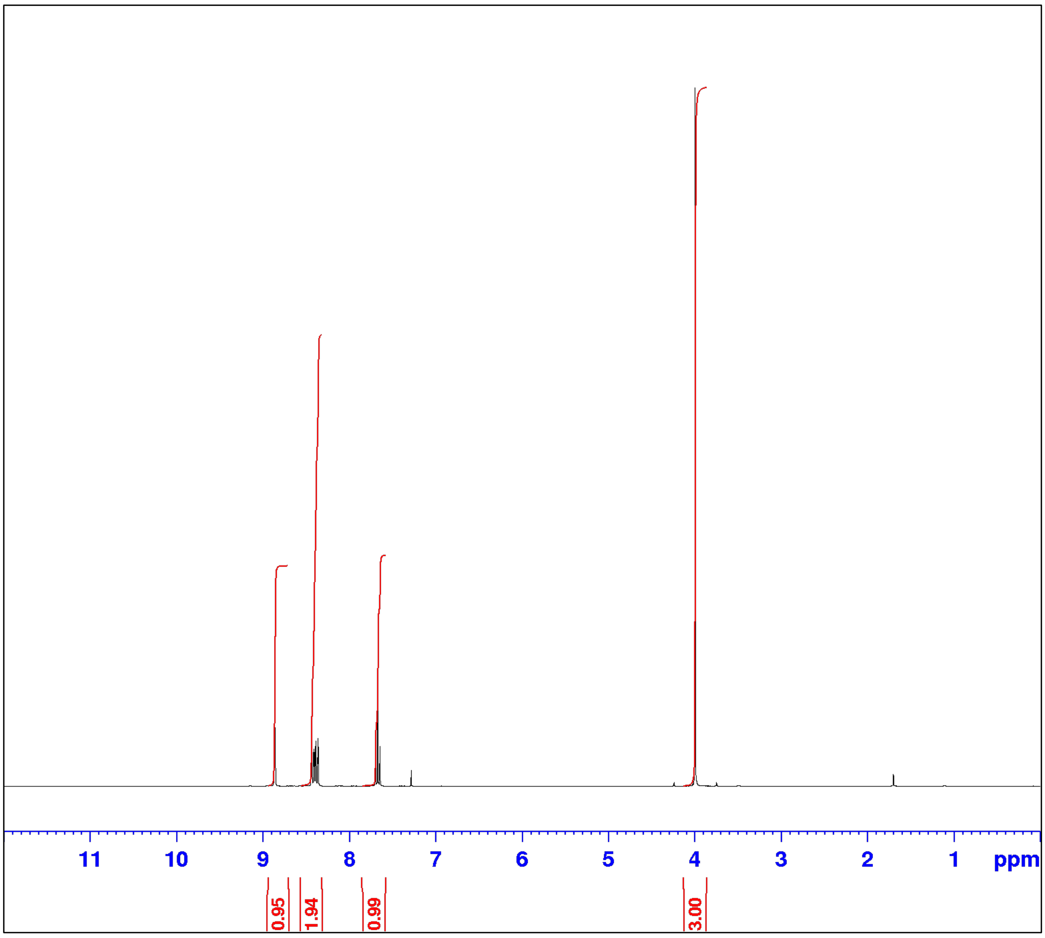


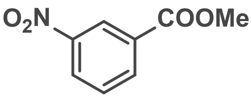


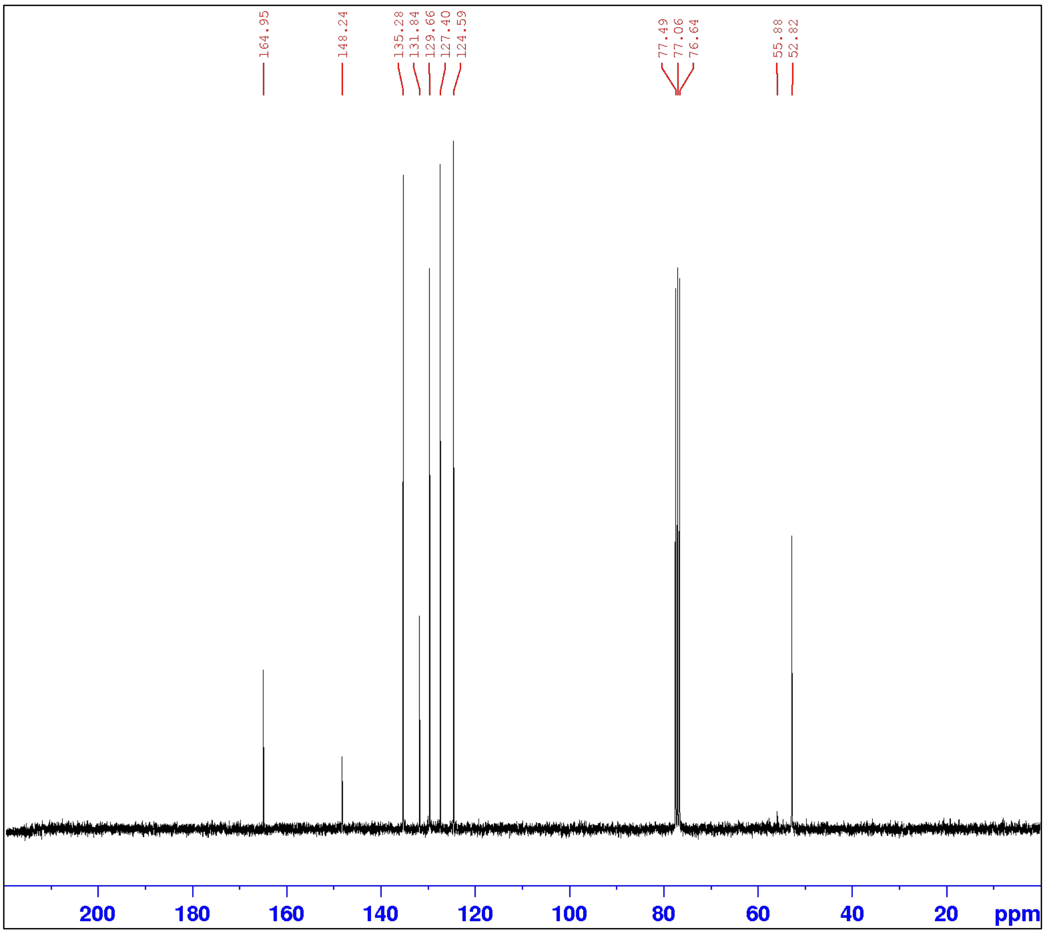


75 MHz, $CDCl_3$

300 MHz, $CDCl_3$

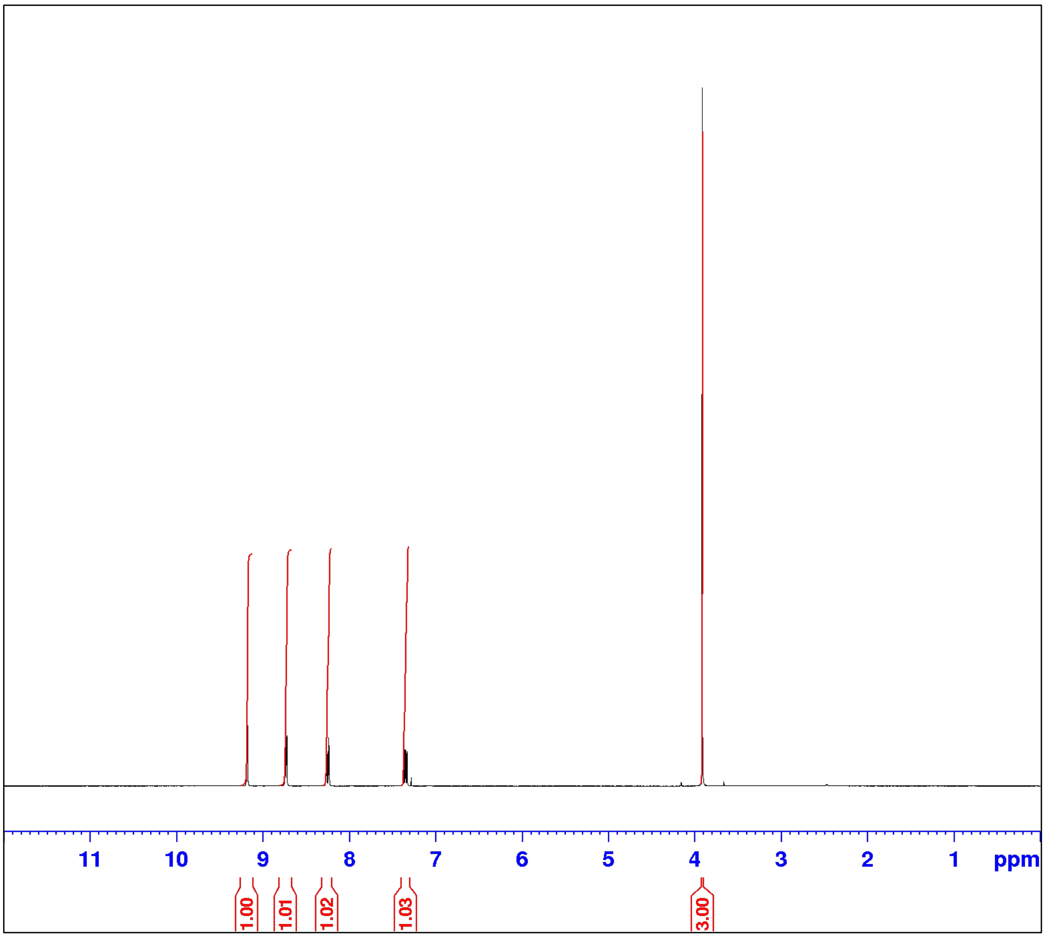


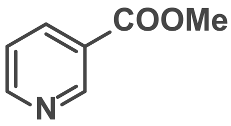


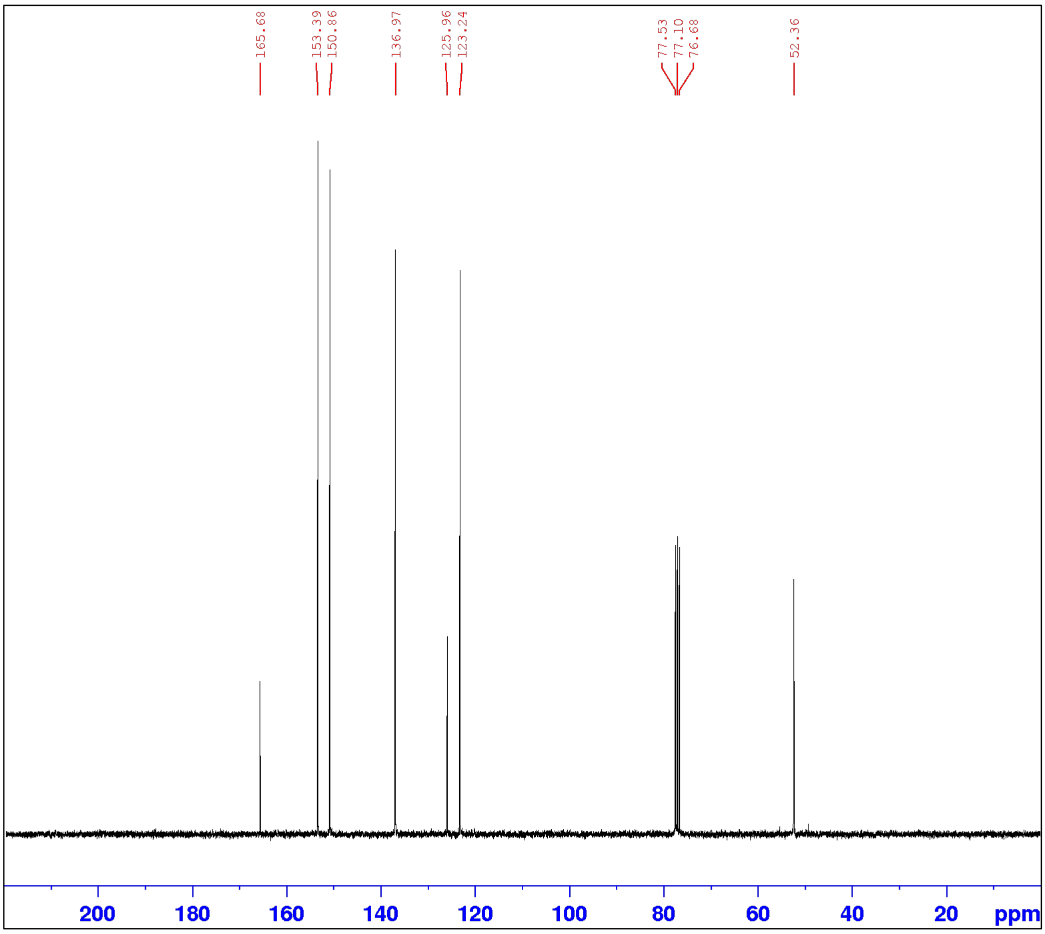


75 MHz, $CDCl_3$

300 MHz, $CDCl_3$

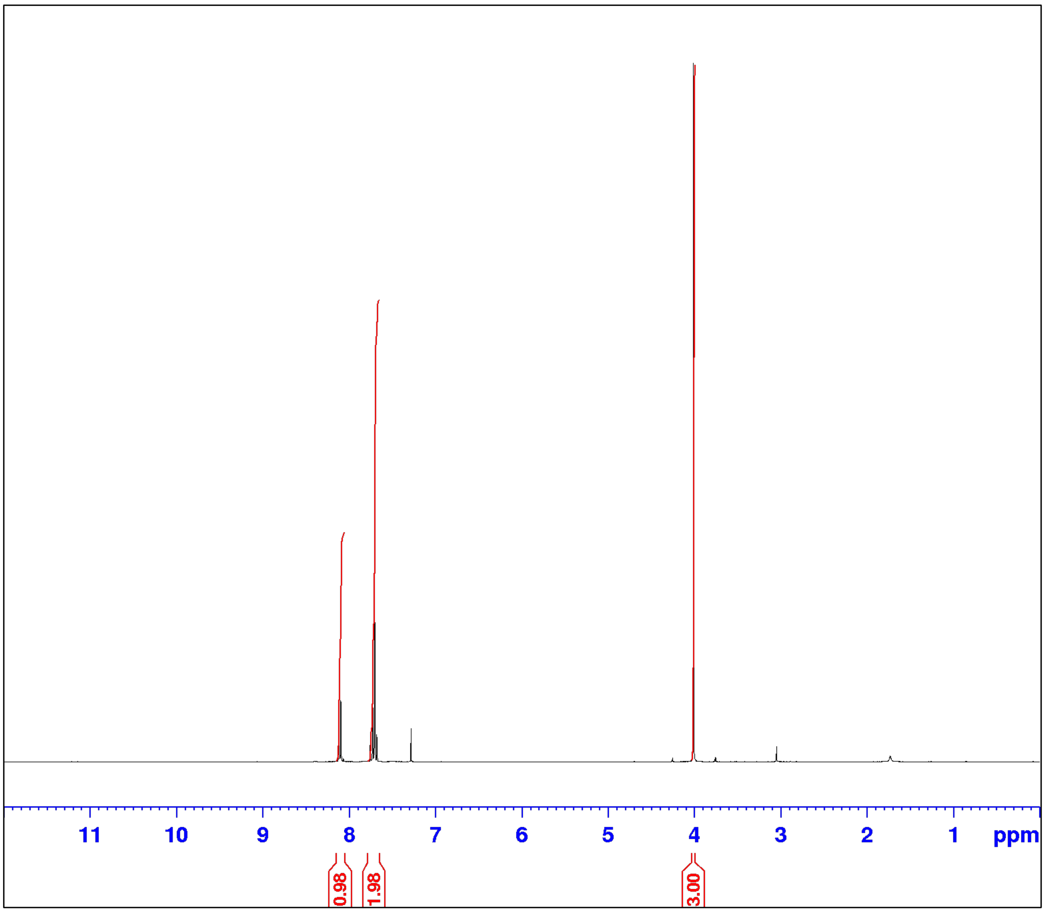


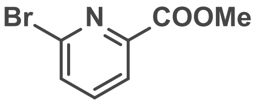


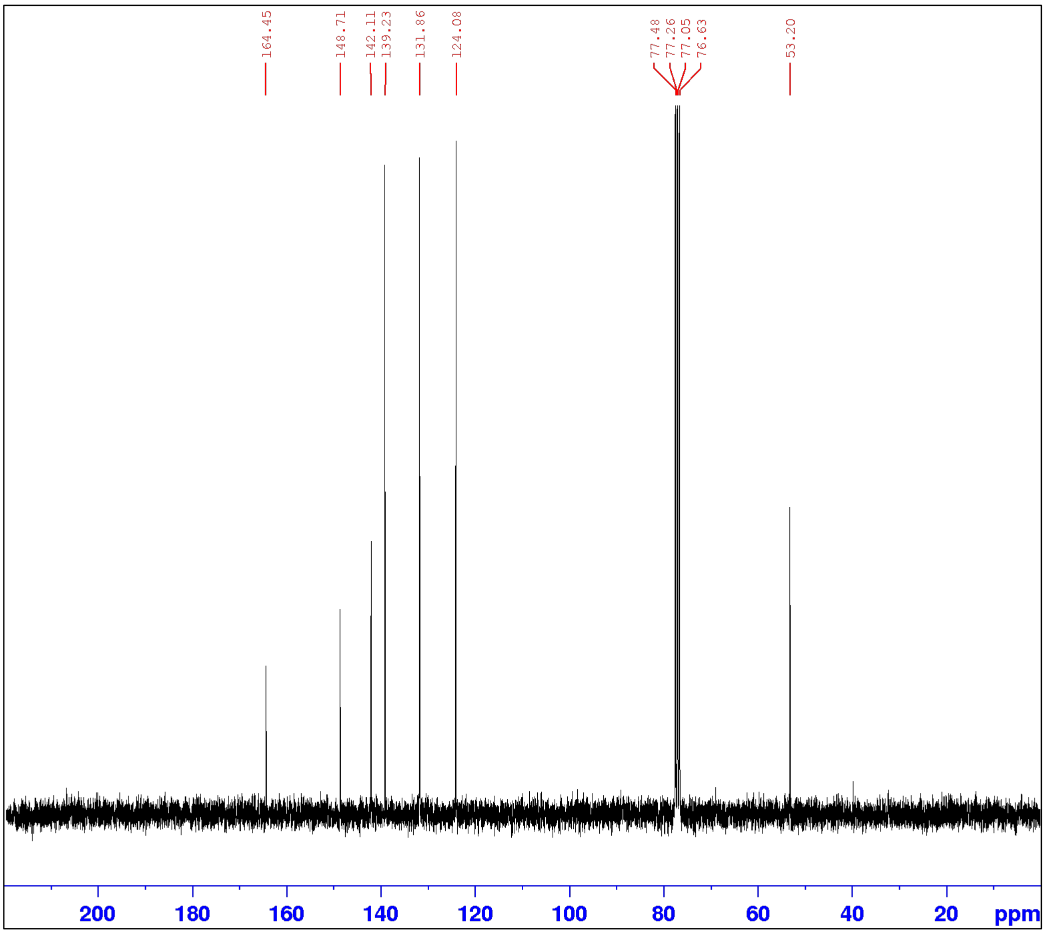


75 MHz, $CDCl_3$

300 MHz, $CDCl_3$

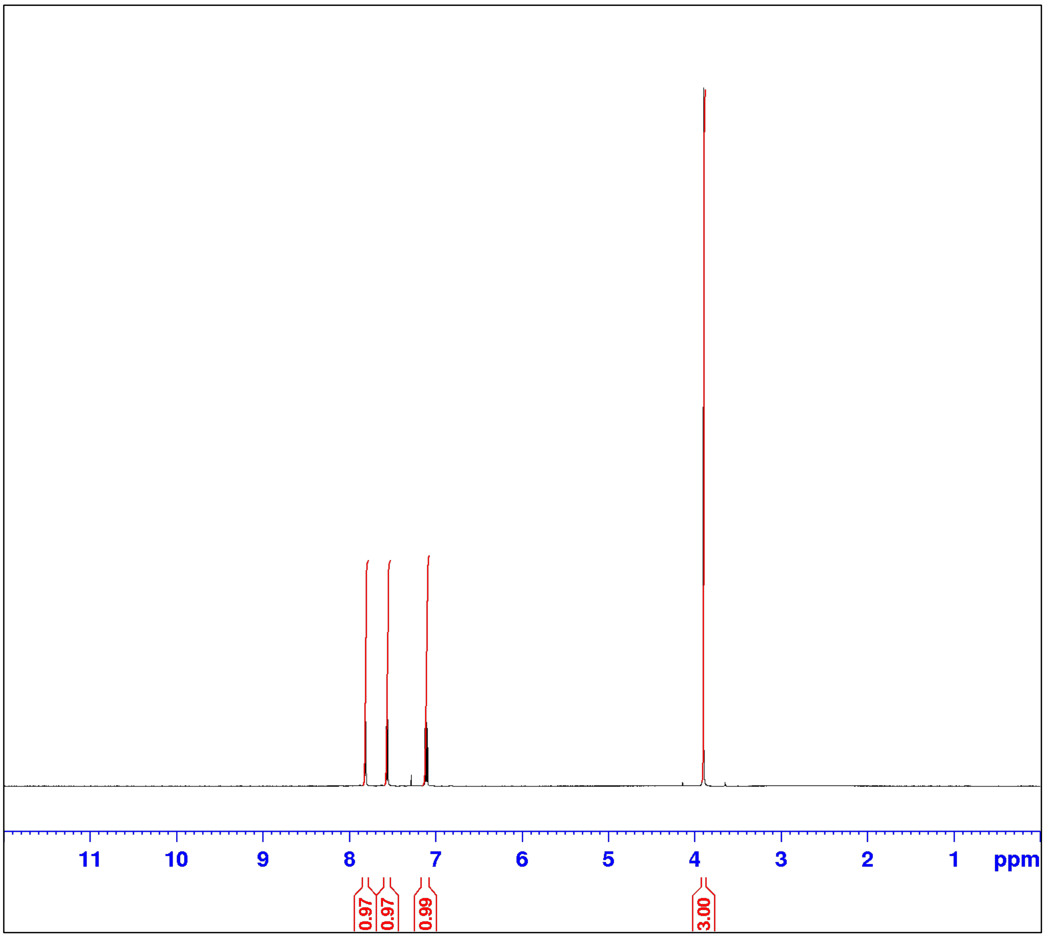


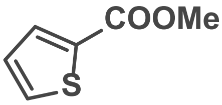


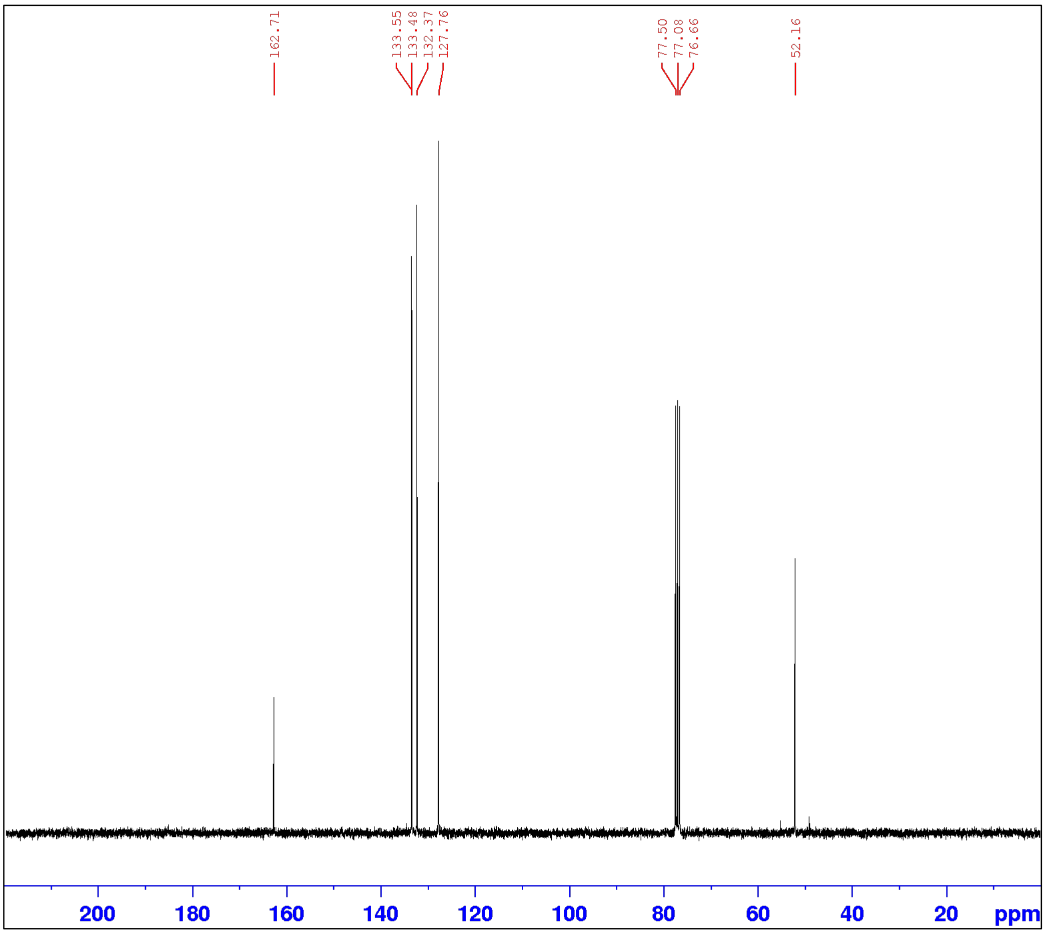


75 MHz, $CDCl_3$

300 MHz, $CDCl_3$

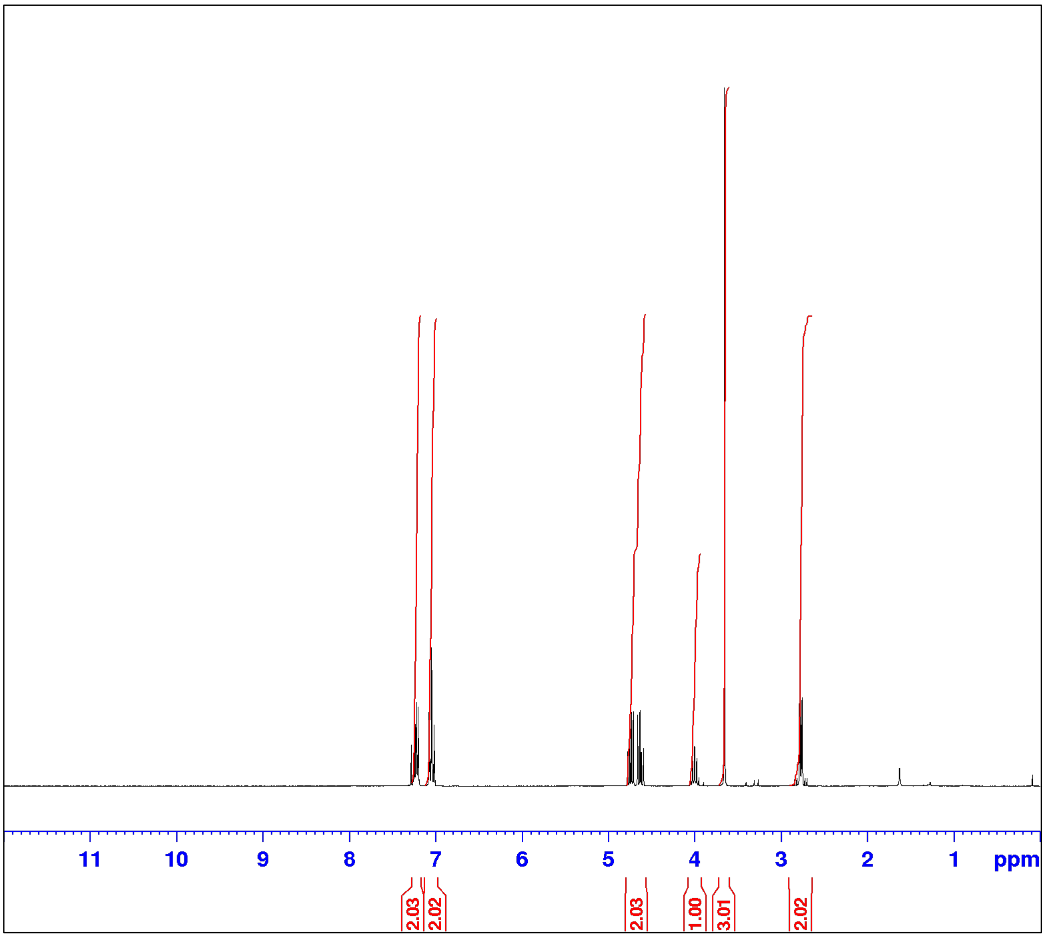


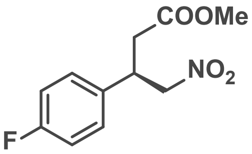


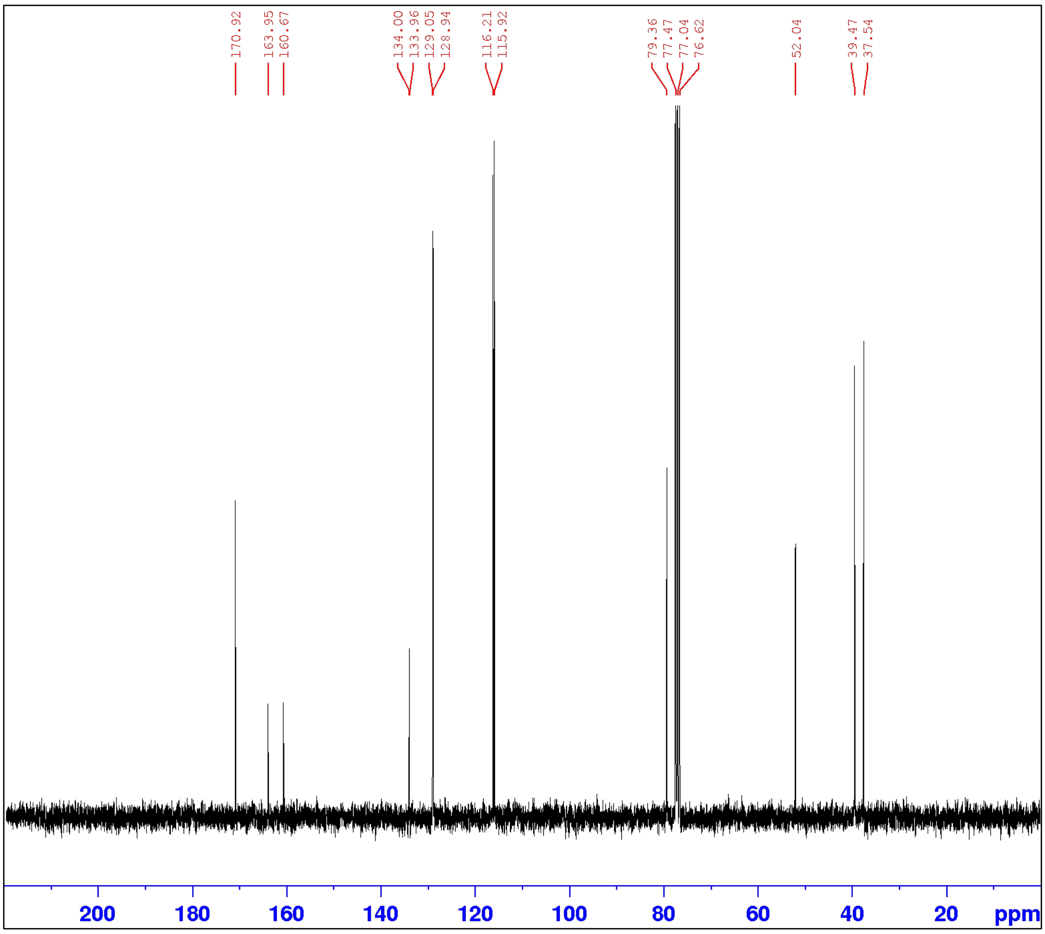


75 MHz, $CDCl_3$

## 6. References


(S1) C. R. McElroy, A. Constantinou, L. C. Jones, L. Summerton and J. H. Clark, *Green Chem.*, **2015**, *17*, 3111-3121.

(S2) B. S. Nagy, P. Llanes, M. A. Pericas, C. O. Kappe and S. B. Ötvös, *Org. Lett.*, **2022**, *24*, 1066-1071.

(S3) T. Shono, Y. Matsumura, K. Inoue and F. Iwasaki, *J. Chem. Soc., Perkin Trans. 1*, **1986**, 73-77.

(S4) Y.-C. Huang and B.-J. Uang, *Chem. Asian J.*, **2014**, *9*, 2444-2448.

(S5) S. S. Sohn and J. W. Bode, *Org. Lett.*, **2005**, *7*, 3873-3876.

(S6) T. O. Vieira, M. J. Green and H. Alper, *Org. Lett.*, **2006**, *8*, 6143-6145.

(S7) M. L. Kantam, R. Chakravarti, V. R. Chintareddy, B. Sreedhar and S. Bhargava, *Adv. Synth. Catal.*, **2008**, *350*, 2544-2550.

(S8) R. Lerebours and C. Wolf, *J. Am. Chem. Soc.*, **2006**, *128*, 13052-13053.

(S9) C. Peng, W. Zhang, G. Yan and J. Wang, *Org. Lett.*, **2009**, *11*, 1667-1670.

(S10) T. Yoshino, S. Imori and H. Togo, *Tetrahedron*, **2006**, *62*, 1309-1317.

(S11) J. Escudero, V. Bellosta and J. Cossy, *Angew. Chem. Int. Ed.*, **2017**, *57*, 574-578.

(S12) H. Li, K. Dong, H. Jiao, H. Neumann, R. Jackstell and M. Beller, *Nat. Chem.*, **2016**, *8*, 1159-1166.

(S13) W. Harnying, P. Sudkaow, A. Biswas and A. Berkessel, *Angew. Chem. Int. Ed.*, **2021**, *60*, 19631-19636.

(S14) M. T. Rahman and S. L. Saha, *J. Organomet. Chem.*, **1980**, *199*, 9-14.

(S15) Y. Wei, B. Rao, X. Cong and X. Zeng, *J. Am. Chem. Soc.*, **2015**, *137*, 9250-9253.

(S16) X.-L. Liu, S.-Y. Lin, S.-R. Sheng, M.-H. Wei and B. Gong, *J. Chin. Chem. Soc.*, **2007**, *54*, 1119-1122.

(S17) F.-L. Wu, B. P. Ross and R. P. McGeary, *Eur. J. Org. Chem.*, **2010**, 1989-1998.

(S18) C. Liu, J. Wang, L. Meng, Y. Deng, Y. Li and A. Lei, *Angew. Chem. Int. Ed.*, **2011**, *50*, 5144-5148.

(S19) A. B. Powell and S. S. Stahl, *Org. Lett.*, **2013**, *15*, 5072-5075.

(S20) J. Cheng, M. Zhu, C. Wang, J. Li, X. Jiang, Y. Wei, W. Tang, D. Xue and J. Xiao, *Chem. Sci.*, **2016**, *7*, 4428-4434.

(S21) J.-F. Li, Y.-F. Wang, Y.-Y. Wu, W.-J. Liu and J.-W. Wang, *Catal.*, **2020**, *150*, 874-880.

(S22) H. Y. Kuchelmeister and C. Schmuck, *Eur. J. Org. Chem.*, **2009**, 4480-4485.

(S23) S. De Sarkar, S. Grimme and A. Studer, *J. Am. Chem. Soc.*, **2010**, *132*, 1190-1191.

(S24) L. Kaganovsky, D. Gelman and K. Rueck-Braun, *J. Organomet. Chem.*, **2010**, *695*, 260-266.

(S25) P. Yin, W.-B. Ma, Y. Chen W.-C. Huang, Y. Deng and L. He, *Org. Lett.*, **2009**, *11*, 5482-5485.

(S26) Y. B. Kiran, R. Ikeda, N. Sakai and T. Konakahara, *Synthesis*, **2010**, *2*, 276-282.

(S27) S. B. Ötvös, P. Llanes, M. A. Pericas and C. O. Kappe, *Org. Lett.*, **2020**, *22*, 8122-8126.
